# Furanoterpene Diversity and Variability in the Marine Sponge *Spongia officinalis*, from Untargeted LC–MS/MS Metabolomic Profiling to Furanolactam Derivatives

**DOI:** 10.3390/metabo7020027

**Published:** 2017-06-13

**Authors:** Cléa Bauvais, Natacha Bonneau, Alain Blond, Thierry Pérez, Marie-Lise Bourguet-Kondracki, Séverine Zirah

**Affiliations:** 1 MCAM UMR 7245, Muséum National d’Histoire Naturelle, Centre National de la Recherche Scientifique (CNRS), Sorbonne Universités, Paris 75005, France; clea.bauvais@gmail.com (C.B.); natacha.bonneau@mnhn.fr (N.B.); blond@mnhn.fr (A.B.); 2 Université Pierre et Marie Curie, Sorbonne Universités, Paris 75005, France; 3 Institut Méditerranéen de Biodiversité et d’Ecologie Marine et Continentale, CNRS UMR 7263, IRD 237, Aix-Marseille Université, Avignon Université, Marseille 13397, France; thierry.perez@imbe.fr

**Keywords:** natural products, furanoterpene, glycinyl lactam, LC MS/MS, NMR spectroscopy, metabolomics, molecular network, marine sponge, *Spongia officinalis*

## Abstract

The Mediterranean marine sponge *Spongia officinalis* has been reported as a rich source of secondary metabolites and also as a bioindicator of water quality given its capacity to concentrate trace metals. In this study, we evaluated the chemical diversity within 30 *S. officinalis* samples collected over three years at two sites differentially impacted by anthropogenic pollutants located near Marseille (South of France). Untargeted liquid chromatography—mass spectrometry (LC–MS) metabolomic profiling (C18 LC, ESI-Q-TOF MS) combined with XCMS Online data processing and multivariate statistical analysis revealed 297 peaks assigned to at least 86 compounds. The spatio-temporal metabolite variability was mainly attributed to variations in relative content of furanoterpene derivatives. This family was further characterized through LC–MS/MS analyses in positive and negative ion modes combined with molecular networking, together with a comprehensive NMR study of isolated representatives such as demethylfurospongin-4 and furospongin-1. The MS/MS and NMR spectroscopic data led to the identification of a new furanosesterterpene, furofficin (**2**), as well as two derivatives with a glycinyl lactam moiety, spongialactam A (**12a**) and B (**12b**). This study illustrates the potential of untargeted LC–MS metabolomics and molecular networking to discover new natural compounds even in an extensively studied organism such as *S. officinalis*. It also highlights the effect of anthropogenic pollution on the chemical profiles within the sponge.

## 1. Introduction

Marine sponges are sessile benthic organisms that fight against biofouling, predation or competition by producing chemical defenses of a great chemical diversity [1,2]. These molecules can represent biotechnological interests in anticancer, antibiotic, anti-inflammatory or analgesic fields. The Mediterranean Demospongiae *Spongia officinalis* Linnaeus, 1759 [3], one of the commercial bath sponges, constitutes a rich source of secondary metabolites, especially sterols such as hydroxy-, seco- and epoxy-sterols as well as terpenoids including diterpenes and furanosesterterpenes and scalarane sesterterpenes [4,5,6,7,8]. Furanoterpenes are particularly diverse and abundant in this sponge and have been suggested as defense molecules within the holobiont [6,7]. For example, furospongin-1 displays antibacterial, antiprotozoal and cytotoxic activities [9,10,11], while furospongin-2 and furospongin-5 exhibit cytotoxic activities [12]. *S. officinalis* has also been shown to harbor a dense and diverse bacterial community [13,14] and to accumulate high concentrations of heavy metals [13,15]. Given the influence of environmental conditions on both bacterial and metal contents within the sponge, *S. officinalis* has been previously proposed as a bioindicator of water quality [14,16]. However, the influence of environmental conditions on the secondary metabolite content within the sponge has not been explored. 

In this study, we assessed the diversity and intra-specific variability of the secondary metabolites of *S. officinalis* by untargeted metabolomics. Our objectives were: (i) to evaluate the influence of site and time on collection on the metabolomic profile within the sponge and presence of potential biomarkers of anthropogenic pollution; and (ii) to assess the potential of metabolomics for the discovery of new natural compounds, even in an extensively explored species. Untargeted LC–MS metabolomic profiling was performed on extracts from sponge samples collected at two sites located near Marseille (South of France), which were differentially impacted by urban sewage in terms of bacterial diversity and metal content as previously reported [13]. In addition, LC–MS/MS analyses and molecular networking were used to search for new compounds related to the metabolomic clustering.

## 2. Results and Discussion

### 2.1. Untargeted Metabolomic Profiles of S. officinalis

**Figure 1:**
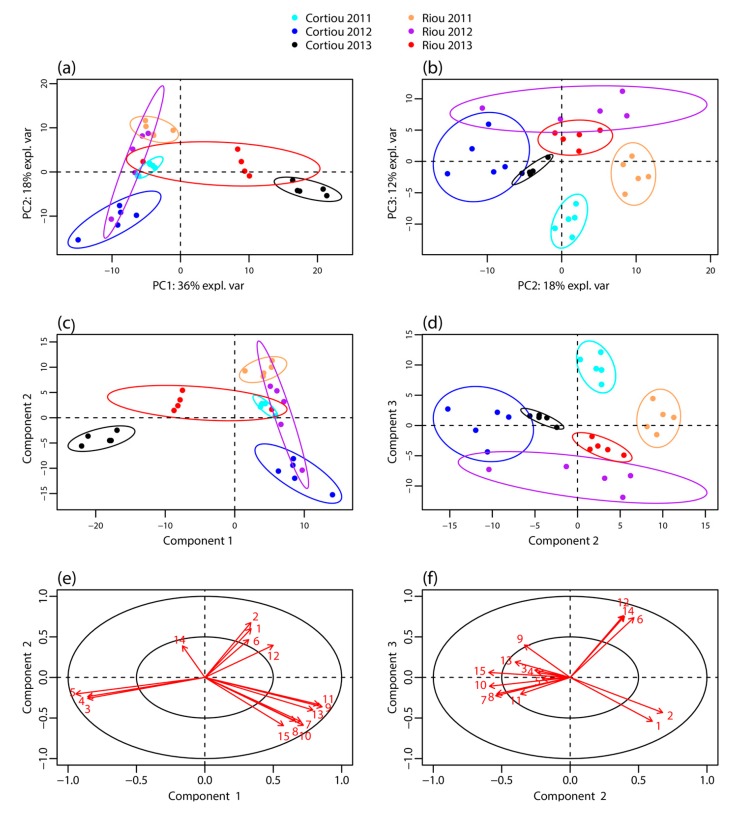
Metabolomic profiling of *S. officinalis* extracts. Score plot of the: PCA (**a**,**b**); and PLS-DA (**c**,**d**) performed from the LC–MS data of the sponge extracts collected at Cortiou and Riou in October 2011, September 2012 and December 2013 (*n* = 30); and (**e**,**f**) PLS-DA loadings corresponding to the main metabolites involved in the clustering. The labels of the variables correspond to the compound numbering indicated in Table 1.

LC–MS profiling of the extracts of *S. officinalis* samples collected in October 2011, September 2012 and December 2013 at Cortiou and Riou was performed by LC–MS in positive ion mode. It revealed a large panel of apolar compounds (Figure S1 in the supplementary materials). The LC–MS data were processed using XCMS Online, which allowed peak detection, automatic retention time alignment and peak matching [17,18]. The generated multivariate matrix consisted of 30 samples and 297 peaks, each characterized by *m*/*z* ratio and retention time. These peaks were assigned to at least 86 compounds from peak-correlation-based annotation with CAMERA [19] (Table S1). The data matrix was submitted to multivariate statistical analysis: principal component analysis (PCA), partial least squares discriminant analysis (PLS–DA) [20] and sparse PLS-DA (sPLS-DA) [21], the latter facilitating the selection of the most discriminant variables. PCA analysis on non-normalized data showed a scale difference between samples from 2013 and those from 2011 and 2012 (Figure S2). Thus the data were mean-centered. The first three components of the PCA of normalized data, PC1, PC2 and PC3, explained 36, 18 and 12% of variance, respectively (Figure 1a,b). Component PC1 mainly showed a clustering differentiating the 2013 samples from those of the other years. Cortiou 2013 revealed a particular profile, while Riou 2013 was intermediate between Cortiou 2013 and the other samples. The PC2/PC3 score plot showed a clustering of the samples per site (Riou and Cortiou, differentiated on PC2) and per year (2011, 2012 and 2013, differentiated on PC3) (Figure 1b). The score plots of the PLS-DA also showed a good separation of the samples, with a clear discrimination of the 2013 samples (Figure 1c,d).

### 2.2. Signals Involved in the Metabolomic Clustering

**Figure 2:**
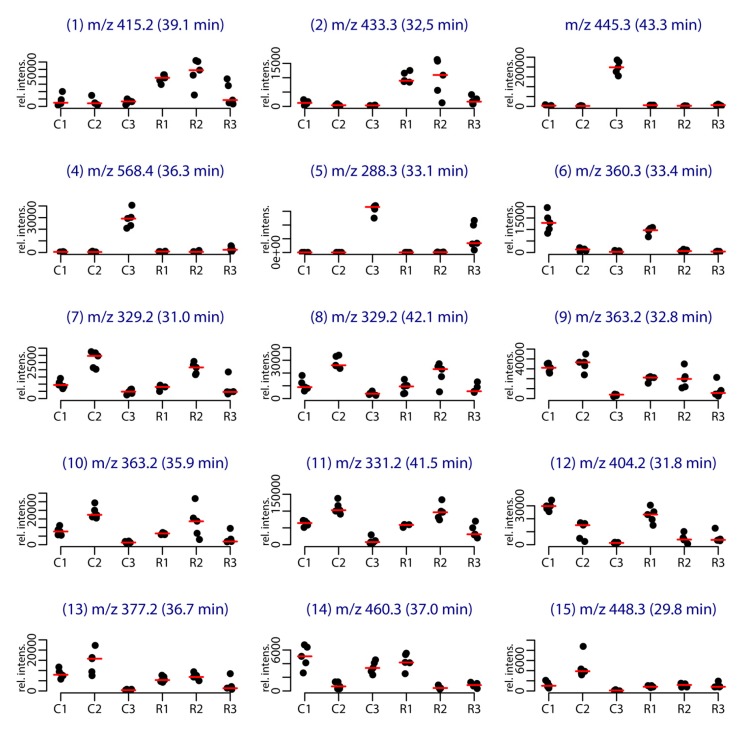
Stripchart representing the intensity of the [M + H]^+^ species of the main metabolites involved in the clustering per site and per year. The labels correspond to the site (C for Cortiou, R for Riou) and year (1, 2, 3 for 2011, 2012 and 2013, respectively).

Among the 86 compounds detected, five compounds were found in all samples and 20 were specific to one sample group (16 specific of Cortiou 2013, and four specific of Riou 2013). The variable importance in projection (VIP) scores obtained for the PLS-DA (Figure S3) and s-PLS-DA analysis (Figure S4) were used to find the peaks involved in the metabolomics clustering. Fifteen compounds were found to be the most discriminant variables (Table 1). Analysis of the scores provided by XCMS Online for the Cortiou/Riou two-group comparison (Table S1), in conjunction with a careful inspection of the LC–MS data in positive and negative ion modes, permitted to select two main compounds involved in the clustering per site: compound **1** ([M + H]^+^ at *m*/*z* 415.2, 39.1 min) and compound **2** ([M + H]^+^ at *m*/*z* 433.3, 32.5 min). These compounds were significantly more represented at Riou (Figure 2), and were correlated together (*p* < 0.05 in Spearman correlation test). The other metabolites associated with the clustering (compounds **3** to **15**) were deducted from the loading plots of sPLS-DA (Figure S4). Compounds **1** to **15** displayed *m*/*z* values ranging from 288.3 to 568.4 in positive ion mode and retention times ranging from 29.8 to 43.3 min (Table 1, Table S2). Their PLS-DA loadings are shown in Figure 1e,f and their relative abundance per site and year is shown in Figure 2. Compounds **3**, **4** and **5**, which were more represented at Cortiou 2013, were correlated two by two (*p* < 0.05 in Spearman correlation test). A correlation was also revealed between compounds **6** and **12**, more represented in 2011.

### 2.3. Compound Annotation through LC–MS/MS and Molecular Networking

**Figure 3:**
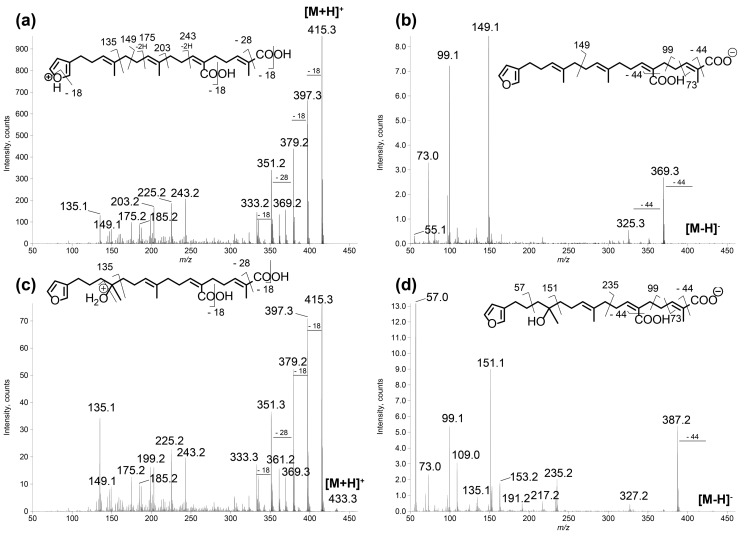
LC–MS/MS spectra of compounds **1** and **2** in: positive (**a**,**c**); and negative (**b**,**d**) ion modes. (**a**) [M + H]^+^ species of compound **1** (*m*/*z* 415.25, CE 20 eV); (**b**) [M − H]^−^ species of compound **1** (*m*/*z* 413.27, CE −40 eV); (**c**) [M + H]^+^ species of compound **2** (*m*/*z* 433.27, CE 20 eV); and (**d**) [M − H]^−^ species of compound **2** (*m*/*z* 431.24, CE −40 eV). The main product ions are shown in the structures.

**Figure 4:**
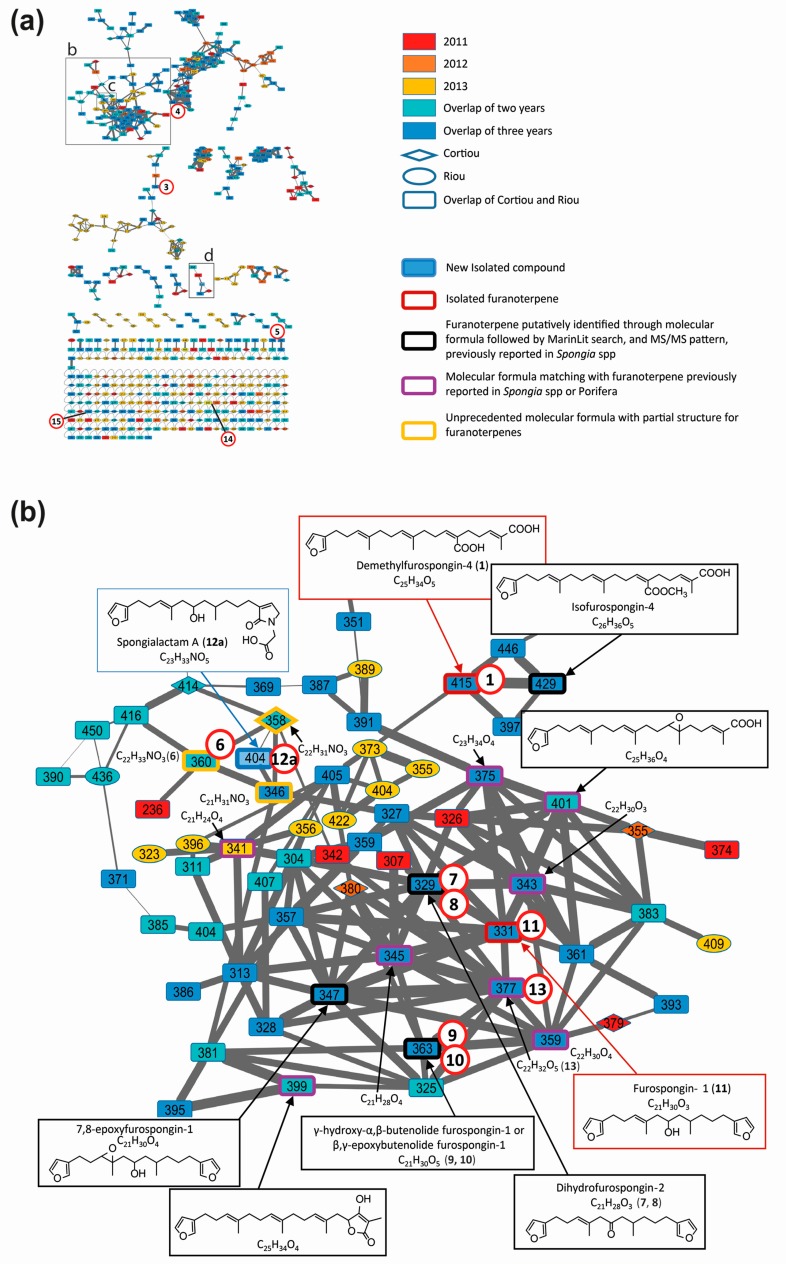

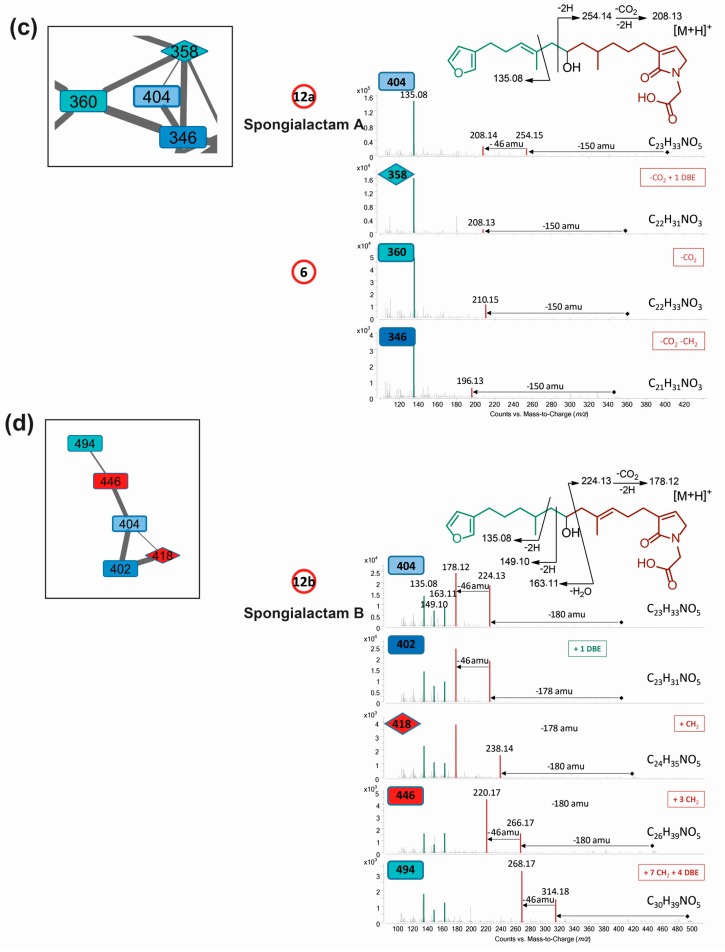
Molecular network of *S. officinalis* extracts: (**a**) global view; (**b**) partial annotation of the furanoterpene cluster (**b**); and (**c**,**d**) enlargements showing the two independent clusters carrying spongialactam A (**12a**) and B (**12b**) ([M + H]^+^ at *m*/*z* 404). Selected spectra from the related nodes are shown (DBE: double bond equivalent, green: furan part, red: glycinyl lactam part). Compounds involved in the metabolomics clustering are shown circled in red.

Ion-dependent LC–MS/MS analyses were performed in positive and negative ion modes on representative samples of each group. Manual inspection at the MS/MS spectra revealed 57 compound MS/MS spectra (51 of good quality and six of low quality, as listed in Table S3). These compounds were not detected in the blank samples. The MS/MS data on the 15 compounds involved in the metabolomic clustering (Figure 3) for compounds **1** and **2** revealed that most of them (**1**, **2**, and **6** to **15**) displayed similarities in their fragmentation pattern, revealing a diagnostic species at *m*/*z* 135 in positive ion mode. This suggested that they belong to the same chemical family. The LC–MS/MS data recorded in positive ion mode at three collision energies were used to generate a molecular network (Figure 4), using the Global Natural Products Social Molecular Networking (GNPS) workflow. This approach permits to cluster compounds based on the similarities of their MS/MS spectra [26,27]. Compounds implicated in differences observed with the metabolomic profiling were searched and annotated in priority. The network consisted of 594 nodes connected with 939 edges, 217 of them appeared as single loops. Among these nodes, 62 were detected in blank samples. One main cluster observed comprised seven nodes whose parent masses matched with 9 out of the 15 previously mentioned compounds (**1** and **6** to **13**, Figure 4b). Although no library hit was found for this cluster through the GNPS workflow, the marine database MarinLit provided fruitful information for manual dereplication based on the molecular formulas proposed from high-resolution mass measurements (Table S2) and MS/MS fragmentation patterns.

− Compound **1** was assigned to the linear furanosesterterpene demethylfurospongin-4, previously isolated from *S. officinalis* [12]. Its identification was confirmed by NMR on the isolated compound (Figures S5 and S6, Table S4). Therefore, the main cluster produced by molecular networking was assigned to the furanoterpene family.
− Compound **2** was not observed in the network due to in-source fragmentation in positive ion mode.
− Compounds **3**, **4** and **5** did not appear in the furanoterpene cluster and appeared structurally unrelated. No structure could be proposed for compounds **3** and **4** based on their molecular masses and fragmentation patterns (Figure S7A,B). Compound **5** displayed a molecular formula and MS/MS spectra consistent with the coconut diethanolamide (C_11_ DEA), a synthetic surfactant considered as marine pollutant (Figure S7C) [22].
− Compound **6** had no match in MarinLit as a furanoterpene molecule based on its assigned molecular formula C_22_H_33_NO_3_ and MS/MS spectrum (Figure S8A).
− Compounds **7** and **8** were assigned to the molecular formula C_21_H_28_O_3_, which matched with two molecules previously isolated from *Spongia* spp.: furospongenone [23] and dihydrofurospongin-2 [25]. The intense *m*/*z* 135 and the *m*/*z* 179 product ions observed for compound **7** were consistent with the structure of dihydrofurospongin-2 (Figure S9A). The corresponding node was thus annotated as a dihydrofurospongin-2 type. The MS/MS spectrum of compound **8** displayed a small product ion at *m*/*z* 135, together with a species at *m*/*z* 149 (Figure S9B). This compound was proposed to contain a dimethyl-allyl backbone, but could not be further identified.
− Compounds **9** and **10** were assigned to the molecular formula C_21_H_30_O_5_ corresponding to a series of furanoterpene isomers isolated from *S. officinalis*, named butenolide furospongin-1 [24]. These compounds contain a furan moiety and either a γ-hydroxy-α-β-butenolide or a β-γ-epoxy butenolide moiety. The fragmentation patterns of compound **9** in positive and negative ion modes (Figure S10A,B) were compatible with butenolide furospongin-1. The chromatographic peak corresponding to this compound had a bimodal peak shape, suggesting a close elution of two isomers. The fragmentation pattern of compound **10** seemed close to that of compound **9**, although much weaker, in particular in positive ion mode (Figure S10C,D). In the literature, no difference in the fragmentation pattern of butenolide furospongin-1 has been reported when a γ-hydroxy-α-β-butenolide is replaced by a β-γ-epoxy butenolide [24], hindering unambiguous identification of compounds **9** and **10**.
− Compound **11** was assigned to the molecular formula C_21_H_30_O_3_, which could correspond to different furanoterpernes isolated from species of the *Spongiidae* family: furospongin-1 [9], tetrahydrofurospongin-2 [25] and furospongenol [23]. The product ions detected in the MS/MS spectrum of the [M + H]^+^ species of this compound (Figure S11) was compatible with the three structures. NMR analysis of the purified compound permitted to assign it to furospongin-1 (Figures S12 and S13, Table S5).
− LC–MS/MS analysis revealed that compound **12** was in fact a mixture of two isomers showing distinct fragmentation patterns, and thus featured into two independent clusters (Figure 4c,d). These compounds were therefore named **12a** and **12b**. Their molecular formula C_23_H_33_NO_5_ had no match in MarinLit as a furanoterpene molecule.
− The molecular formula C_22_H_32_O_5_ assigned to compound **13** (Table S2, Figure S8B) matched with irciformonins B and J isolated from *Ircinia formosana* [28,29]. The compound fragmentation pattern was consistent with the structure of irciformonin B.
− Compounds **14** and **15**, assigned to the molecular formulas C_27_H_41_NO_5_ and C_28_H_33_NO_4_, respectively, appeared outside the furanoterpene cluster. However, their fragmentation patterns included the diagnostic ion *m*/*z* 135 which could imply their annotation as furanoterpenes with unusual product ions (Figure S8C,D).

Besides the 15 compounds involved in the metabolomic clustering, several nodes belonging to the furanoterpene cluster were annotated, matching with molecules of isofurospongin-4 type, 7,8-epoxyfurospongin-1 type, or tetronic acid derivatives (Figure 4a). Five molecular formulas were found to match with furanoterpenes previously reported in the *Porifera* phylum.

Dereplication of the main cluster featured on the molecular network, supported by the isolation and characterization of two representatives: demethylfurospongin-4 (**1**) and furospongin 1 (**11**), permitted to identify or at least provide partial structure of several metabolites involved in the untargeted metabolomic profiling and to highlight the diversity of furanoterpenes in *S. officinalis*. The molecular network guided the subsequent isolation work, highlighting three new compounds from the furanoterpene cluster, compounds **2**, **12a** and **12b**, which were isolated. Their structure elucidation is reported below.

### 2.4. Structure Elucidation of New Furanoterpene Derivatives

**Figure 5:**
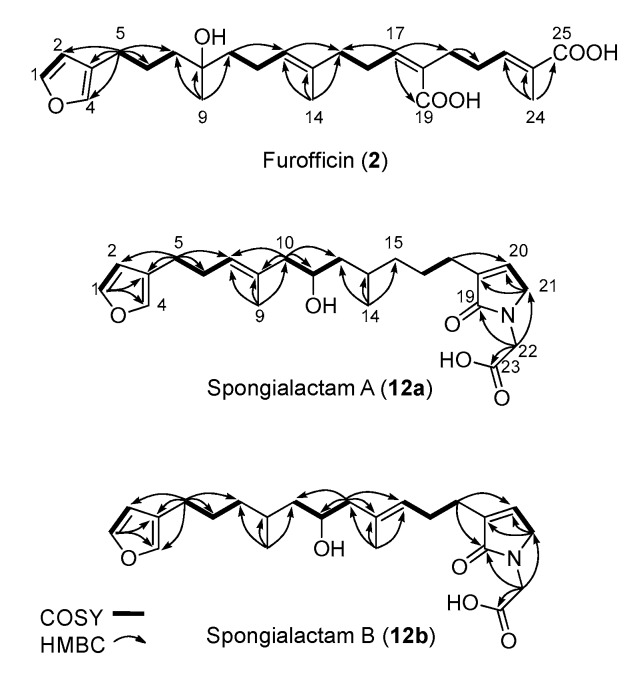
Selected COSY and HMBC correlations for compounds **2**, **12a** and **12b**.

**Figure 6:**
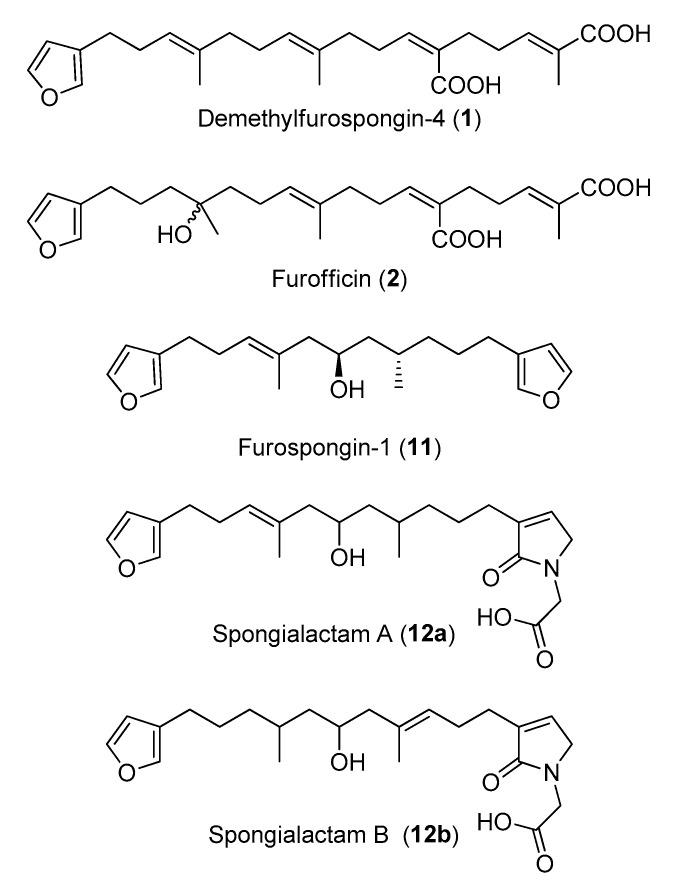
Structures of the compounds isolated in this study from the marine sponge *Spongia officinalis*.

The new furanoterpenes **2**, **12a**, **12b**, were isolated by successive steps of reverse-phase high-performance liquid chromatography (RP-HPLC). Compound **2** was isolated as a white amorphous solid. Its molecular formula C_25_H_36_O_6_ deduced from high resolution mass measurement indicated the presence of eight degrees of unsaturations. ^1^H NMR and HSQC data evidenced a furan moiety (δ_H_ 6.30, 7.27, 7.38; δ_C_ 111.6, 126.0, 139.8, 143.6), three ethylenic protons (δ_H_ 6.61, 5.31, 5.15) and three methyl groups (δ_H_ 1.13, 1.61, 1.81) (Table 2, Figures S14–S18). LC–MS/MS analysis of compound **2** revealed a fragmentation very close to that of demethylfurospongin-4 (**1**) in positive ion-mode, while negative mode revealed clearly distinct fragmentation patterns for the two compounds (Figure 3). The MS/MS spectrum of the compound **2** in negative ion mode ([M − H]^−^ at *m*/*z* 431.2) showed product ions at *m*/*z* 151 (C_9_H_11_O_2_^−^) and *m*/*z* 235 (C_15_H_23_O_2_^−^), suggesting the presence of a hydroxyl function and a dimethyl-allyl chain linked to the pentylfuran skeleton, respectively. In addition, the neutral losses of 44 u (*m*/*z* 387, C_24_H_35_O_4_^−^) and 88 u (*m*/*z* 343, C_23_H_35_O_2_^−^) indicated the presence of two carboxylic acid functionalities. The presence of the hydroxyl function was confirmed by NMR from the oxyquaternary carbon signal (δ_C_ 73.1 ppm) and the HMBC correlation from the methyl singlet at δ_H_ 1.13 (H-9) to this signal, which allowed localizing the hydroxyl at position C-8. HMBC correlations from the methyl at δ_H_ 1.81 (H-24) to the carbon at δ_C_ 174.9 (C-25) and from the olefinic triplet at δ_H_ 5.31 (H-17) to the carbon at δ_C_ 177.5 (C-19) permitted to localize the carboxylic acid functions. The *E* geometry of the trisubstituted double bonds C-12/C-13, and C-22/C-23 was assigned based on the upfield resonance of C-14 (δ_C_ 15.6) and C-24 (δ_C_ 13.0), as reported for demethylfurospongin-4 [30,31,32]. It was supported by NOESY correlations between H-11/H-14, H-12/H-15, and H-21/H-24.The *Z* geometry of C-17/C-18 was assigned by NOESY correlations between H-17/H-20. Selected ^1^H-^1^H orrelated spectroscopy (COSY) and heteronuclear multiple bond correlation (HMBC correlations of compound **2** are presented in Figure 5. The new compound **2** (Figure 6) was named furofficin.

**Figure 7:**
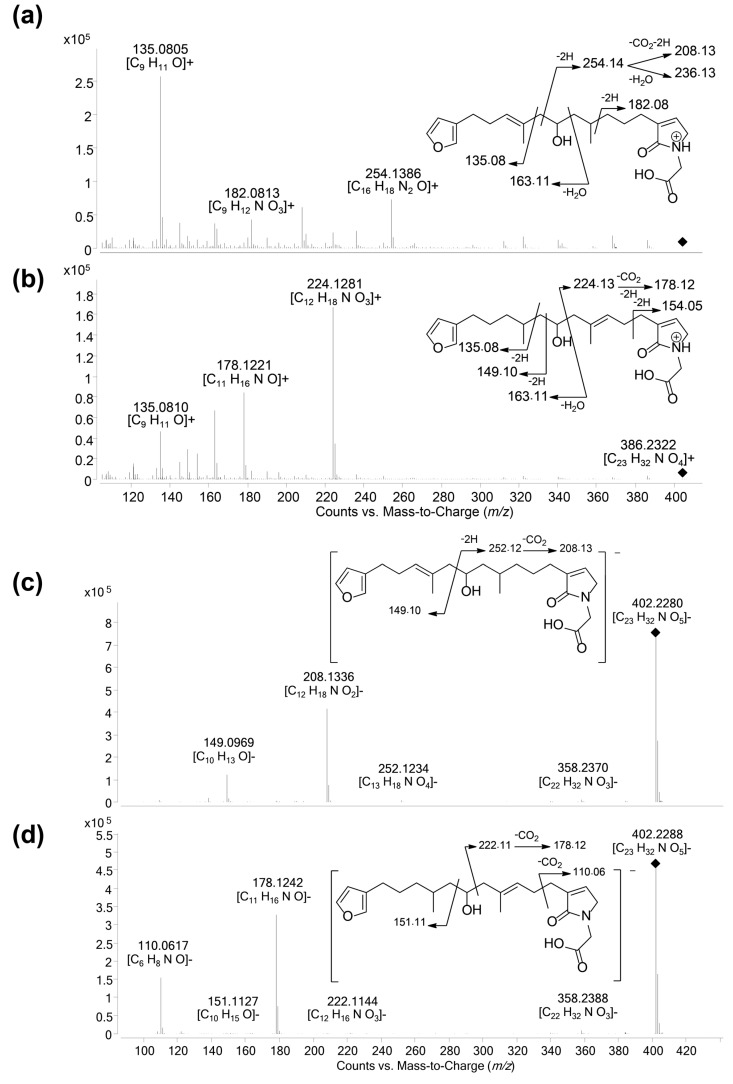
LC–MS/MS spectra of spongialactam A (**12a**) and spongialactam B (**12b**) in positive mode: [M + H]^+^ at *m*/*z* 404.2, CE 20 eV (**a**,**c**); and negative mode: [M − H]^−^ at *m*/*z* 402.2, CE −20 eV (**b**,**d**). The main product ions are shown on the structures.

Compound **12a** was isolated as a white amorphous solid. The molecular formula was established as C_23_H_33_NO_5_, which indicated 8 degrees of unsaturation. The ^1^H NMR and HSQC data recorded in methanol-*d*_4_ (Table 2, Figures S19–S23) evidenced a furan moiety (δ_H_ 6.31, 7.25 7.37; δc 112.0, 126.0, 140.0, 143.7), one trisubstituted double bond (δ_H_ 5.21, t, 7.1), a vinylic methyl group (δH 1.61, s), a secondary methyl doublet (δ_H_ 0.88, d, 6.6), and one oxymethine (δ_H_ 3.76, δc 68.1), showing similarities with the known furospongin-1 (**11**) (Figures S12 and S13, Table S5). LC–MS/MS analysis of compound **12a** (Figure 7a,b), Tables S4 and S5) revealed several product ions in favor of a dimethyl-allyl-furanyl sequence as observed for compound **1** (Figure 3 ) the *m*/*z* 135 (C_9_H_11_O^+^) and *m*/*z* 149 (C_10_H_13_O^−^) species in positive and negative ion modes, respectively. The *E* geometry of the trisubstituted olefin was assigned based on the upfield resonance of the vinylic methyl carbon (δ_C_ 16.5, C-9), as reported for furospongin-1 [11] and was supported by NOESY correlations between H-6/H-9 and H-7/H-10. HMBC correlations between the methylene at δ_H_ 2.05, 2.15 (H-10) and the carbon at δ_C_ 68.1 (C-11) allowed the localization of the hydroxyl in position C-11. This assignment was supported by the product ion at *m*/*z* 163 (C_11_H_15_O^+^) also detected for compound **11** (furospongin-1), which corresponds to a cleavage of C11-C12. In addition, HMBC correlations between the methyl doublet at δ_H_ 0.88 (H-14) and the carbons at δ_C_ 30.1 (C-13), δ_C_ 45.4 (C-12), δ_C_ 38.8 (C-15) allowed to localize the methyl group in position 14. Relative configuration of C-11 and C-13 was *anti* as for furospongin-1, according to NOESY correlations of the oxymethine δH 3.76 (H-11) with the methyl group δ_H_ 0.88 (H-14). Comparison of ^1^H NMR and HSQC spectra of **12a** with furospongin-1 (**11**) revealed that the second furan moiety was lacking and that additional signals were observed at δ_H_/δ_C_ 6.83/137.7, 4.06/53.0 and 4.05/46.7. These data combined with the presence of additional carbon signals at δ_C_ 138.2, 173.8 and 175.5 suggested the presence of a glycinyl-lactam function, a feature previously reported for sponge sesterterpenes such as ircinialactams and ianthellalactams [30,32,33]. Key HMBC correlations between the methylene at δ_H_ 2.24 (H-17) and carbons at δ_C_ 140.1 (C-18), 173.8 (C-19), 137.7 (C-20), and between the methylene at δ_H_ 4.05 (H-22) and the carbons at δ_C_ 173.8 (C-19), 53.0 (C-21), 175.5 (C-23) confirmed this structure (Figure 5, Table 2). The MS/MS spectra of **12a** showed product ions carrying the glycinyl-lactam function resulting from the hydroxyl α-cleavage, at *m*/*z* 254 (C_13_H_20_NO_4_^+^) and *m*/*z* 252 (C_13_H_18_NO_4_^−^) in positive and negative mode, respectively. This cleavage could be combined with a loss of 46 u (in positive mode) or 44 u (in negative mode), to yield product ions at *m*/*z* 208. The new compound **12a** (Figure 6) was named spongialactam A.

Compound **12b**, isolated as a white amorphous solid, displayed the same molecular formula C_23_H_33_NO_5_ as spongialactam A (**12a**). The NMR data indicated the presence of furan, trisubstituted olefin, hydroxyl and glycinyl-lactam cores in the two isomers (Table 2, Figures S24–S28). However, the MS/MS spectra of the two compounds were different (Figure 7, Figures S4–S7). In positive mode, the relative intensity of the product ion at *m*/*z* 135 (C_9_H_11_O^+^) and *m*/*z* 163 (C_11_H_15_O^+^) was inverted, the latter being favored for **12b**. In negative mode, the product ion at *m*/*z* 149 (C_10_H_13_O^−^) detected for spongialactam A (**12a**) was replaced by a species at *m*/*z* 151 (C_10_H_15_O^−^) for **12b**. Such difference, already observed between demethylfurospongin-4 (**1**) and furofficin (**2**) (Figure 3), suggested a loss of the C7-C8 unsaturation in the **12b** isomer. The product ions containing the glycinyl-lactam function (even *m*/*z* value), detected at *m*/*z* 224 (C_12_H_18_NO_3_^+^), *m*/*z* 178 (C_11_H_16_NO^+^) and *m*/*z* 154 (C_7_H_8_NO_3_^+^) in positive mode and *m*/*z* 222 (C_12_H_16_NO_3_^−^), *m*/*z* 178 (C_11_H_16_NO^−^) and *m*/*z* 110 (C_6_H_8_NO^−^) in negative mode, supported the presence of a hydroxyl group at C-11 and permitted to propose a double bond in C-12/C-16 region. The double bond localization at C-13/C-15 was provided by the COSY correlations between the ethylenic proton at δ_H_ 5.21 (H-15) and the methylene at δ_H_ 2.29 (H2–16) and HMBC correlations from this ethylenic proton to the carbon signals at δ_C_ 16.1 (C-14) and 27.0 (C-16) (Figure 5). The *E* configuration of the double bond was assessed by the upfield resonance of the vinyl methyl carbon (δc 16.1, C-14) and the relative configuration *anti* of C-8/C-11 was supported by NOESY correlations, as for **11** and **12a**. The new compound **12b** (Figure 6) was named spongialactam B.

### 2.5. Unravelling Pyrrolofuranoterpene Derivatives from S. officinalis

Spongialactams A (**12a**) and B (**12b**) appeared in two separate clusters in the molecular network (Figure 4c,d), due to high differences in fragmentation attributed to the position of the double bond. This allowed spotting some of their relatives, joining the ranks of this uncommon furanolactam family (Table S6).

Spongialactam A (**12a**) revealed a fragmentation pattern with a loss of 150 u from the precursor ion, corresponding to the dimethyl-allyl-furan. This trend was also observed for the closest nodes with precursor ions at *m*/*z* 360 (compound **6**), *m*/*z* 358 and *m*/*z* 346, assigned to the molecular formulas C_22_H_33_NO_3_, C_22_H_31_NO_3_ and C_21_H_31_NO_3_, respectively (Figure 4c). Among these nodes, only spongialactam A (**12a**) showed a 46 u loss from the M-150 species, which might be in favor of a simple pyrrolone instead of a glycinyl-lactam moiety for the other compounds. These compounds were thus proposed as pyrrolo-furanoterpenes. Compound **14** (C_27_H_41_NO_5_), although placed outside this cluster, noticeably displayed fragmentation similarities with compounds **6** and **12a**, in terms of mass differences between product ions, i.e., −150 u, −46 u and −30 u losses (Figure S8). 

Spongialactam B (**12b**) was allocated to a small cluster (Figure 4d), together with other nodes at *m*/*z* 402, *m*/*z* 418, *m*/*z* 446 and *m*/*z* 494, assigned to the molecular formulas C_23_H_31_NO_5_, C_24_H_35_NO_5_, C_26_H_39_NO_5_ and C_30_H_39_NO_5_, respectively. The loss of 180 u observed for most of the nodes was attributed to a release of the furan part through hydroxyl α-cleavage. Nodes corresponding to *m*/*z* 418, 446 and 494 were found to present on the glycinyl-lactam side one additional CH_2_, three additional CH_2_ and 7 additional CH_2_ together with 4 double bond equivalents (DBE), respectively, as compared with **12b**. The node at *m*/*z* 402 showed identical fragmentation pattern as **12b**, indicating structural similarity on the glycinyl-lactam side of the molecule. The additional unsaturation was thus located on the furan side of the molecule.

Although the furanoterpenes from *Spongia* species have been extensively studied, the analysis of *S. officinalis* extracts by LC–MS permitted to reveal a number of new members of this family, such as the nitrogen-containing compounds **6**, **12a**, **12b** and **14**. This is the first time that furanoterpenes including a glycinyl lactam moiety are reported from a *Spongia* species. Glycinyl lactam terpenes have been previously isolated from sponges [5] including pyrrolosesterpenes [31,32,33,34] and trinorsesterterpenoid lactams from the Dictyoceratida marine sponge *Sarcotragus* [35] and hippospongin C from the marine sponge *Hippospongia* sp. [36].

MS/MS in positive ion mode provided a diagnostic ion for derivatives with a dimethyl-allyl-furan substructure at *m*/*z* 135, assigned to C_9_H_11_O^+^. This species was also reported as intense product ion generated by MS/MS of M^+^ molecular ions generated by electron impact ionization [12,28,37]. MS/MS in negative ion mode furnished several signatures informative on the chemical groups present. For example, the product ions at *m*/*z* 99 and *m*/*z* 73 were characteristic of representatives with carboxylic acid groups such as compounds **1** and **2**, while the species at *m*/*z* 69 and *m*/*z* 57 were observed specifically for compounds **9** and **10**, carrying a tetronic acid or an epoxybutenolide moiety.

### 2.6. Metabolite Variability and Furanoterpene Signature

Various marine species exhibit variations in their secondary metabolome according to their geographic location, as illustrated with gorgonians *Annella mollis* and *A. reticulate* [38], the nudibranch *Asteronotus cespitosus* [39] and the sponges *Acanthella cavernosa* [40], *Spongia lamella* [41] and *Aplysina cavernicola* [42]. Essential factors such as water temperature, light exposure, chemical contamination or food availability should be considered for additional ecological interpretations of the chemical variability over space and time [43].

In this study, the linear furanosesterterpenes demethylfurospongin-4 (**1**) and the new furofficin (**2**) were identified as the main compounds explaining the variability between the two sites, Cortiou and Riou, both of them being found in higher concentration in Riou (Figure 2). However the clustering was mainly influenced by the year, with higher concentrations of pyrroloterpenes (compounds **6**, **12a**, **12b**, and **14**) in 2011, or of C_21_ bisfuranoterpenes of dehydrofurospongin-2 and furospongin-1 types (compounds **7**, **8** and **11**) in 2012. Lower concentrations were however spotted for furospongin-1 butenolide derivatives (compounds **9** and **10**) in 2013. Furthermore, clusters for one site/one year were also noticed. In this study, the particular metabolomic profile observed for the samples collected at Cortiou in 2013 was partly explained by the presence of the marine pollutant coconut C_11_ diethanolamide. It coincided with a high copper content measured in sponge and seawater samples [13]. The detection of these two pollutants within the sponge at this time point suggests a local anthropogenic pressure, which can be sensed within *S. officinalis*. Although evaluation of the water quality was only based on metal content measurements, these observations support the potential of this sponge as a bioindicator of water quality. 

Certain Demospongiae harbor a stable, dense and diverse microbial community, constituting up to 40% of the host volume [44,45]. In many cases, the bioactive natural compounds from the sponge holobiont can be ascribed to associated microorganisms, which thus contribute to the holobiont metabolism and defense [46,47,48]. To the best of our knowledge, it is not clear whether demethylfurospongin-4, previously isolated as one of a major furanoterpene from *S. officinalis*, is produced by the sponge itself or by its associated microbiota. If furanoterpenes are most probably biosynthesized by the sponge itself [49], the wide diversity of these compounds in the sponge holobiont could result from biotransformations by the hosted microorganisms [50] or variations in the bacterial communities associated with *S. officinalis* [13]. 

## 3. Materials and Methods 

### 3.1. Materials 

Specimens of *S. officinalis* (Demospongiae, Dictyoceratida, Spongiidae) were collected by scuba diving at Cortiou and Riou (France) at 10 and 18 m depth, in October 2011, September 2012 and December 2013 (five samples for each collection) [13]. Cortiou, located 300 m east of the discharge outlet in the vicinity of the city of Marseille, is known to be strongly influenced by sewage from Marseille and its suburb [15]. Riou is an island located 3 km off the coast (4 km from Cortiou); therefore, it is much less affected by anthropogenic pressure. The freshly collected samples were lyophilized and stored at −80 °C. 

Dichloromethane, methanol and acetonitrile were purchased from Carlo Erba (Product number 528372, stabilized with ethanol), VWR (Product numbers 20864.320 and 20060.320, Hipersolv Chromanorm for HPLC), respectively.

### 3.2. Sponge Extraction 

After lyophilization, samples (dry weight, 2 g) were ground to powder and extracted with a mixture of CH_2_Cl_2_/MeOH 1:1 (3 × 60 mL, sonication for 15 min at room temperature). The CH_2_Cl_2_/MeOH extracts were concentrated under reduced pressure to yield a yellow powder, which after mixing with 2 g of C18 silica (50 µm, 65 Å-Phenomenex) was loaded on a pre-packed Strata^®^ C18-E cartridge (2 g/12 mL-Phenomenex, Le Pecq, France) to perform a solid phase extraction. The cartridges were washed with 10 mL H_2_O and eluted with 10 mL of a 1:1 CH_2_Cl_2_/MeOH mixture. 

### 3.3. LC–MS Analyses

For metabolomic profiling, LC–MS of the sponge extracts was performed on an Ultimate 3000 Micro-HPLC system (Thermo Scientific) connected to an ESI-Qq-TOF Q-STAR Pulsar mass spectrometer (Sciex) equipped with an IonSpray source. The sponge extracts were subjected to HPLC (C18 Uptisphere WTF Interchim, 150 × 1 mm, 300 Å, 5 μm, column, 40 µL/min gradient elution, 90–20% CH_3_CN/H_2_O with an isocratic 0.1% HCOOH, over 35 min). The injection volume was 1 µL. Blank samples consisted of solvent alone, i.e., CH_2_Cl_2_/MeOH 1:1. The MS data were collected in positive ion mode in the *m*/*z* range 250–1500. Representative samples (mix of samples from Riou and one representative sample from Cortiou 2011, 2012 and 2013) were also analyzed in positive or negative ion mode in ion-dependent acquisition mode to generate automatic MS/MS spectra on the main ions detected. Finally, the ions of interest were analyzed by LC–MS/MS in positive or negative ion mode at 20, 30 and 40 eV.

For molecular networking and analysis of the compounds **2**, **12a** and **12b**, LC–MS/MS experiments were acquired on an Agilent 1260 HPLC (Agilent Technologies) coupled to an Agilent 6530 Q-ToF-MS equipped with a Dual ESI source. The chromatographic separation was performed using an HPLC (C18 Sunfire^®^ Waters Saint-Quentin-en-Yvelines, France, 150 × 2.1 mm, 3.5 µm column, 250 µL/min gradient elution, 30–85% CH_3_CN/H_2_O with an isocratic 0.1% HCOOH, over 30 min). The divert valve was set to waste for the first 3 min. In positive ion mode, purine C_5_H_4_N_4_ [M + H]^+^ ion (*m*/*z* 121.0509) and hexakis (1H, 1H, 3H-tetrafluoropropoxy) phosphazine C_18_H_18_F_24_N_3_O_6_P_3_ [M + H]^+^ ion (*m*/*z* 922.0098) (HP 0921) were used as internal lock masses. Source parameters were set as follow: capillary voltage at 3500 V, gas temperature at 320 °C, drying gas flow at 10 L/min, nebulizer pressure at 40 psi. Fragmentor was set at 175 V. Acquisition was performed in auto MS^2^ mode on the range *m*/*z* 100–1200 with an MS rate of 1 spectra/s and an MS/MS scan rate of 3 spectra/s. Isolation MS/MS width was 4 u. Fixed collision energies 20, 30, and 40 eV were used. MS/MS events were performed on the three most intense precursor ions per cycle with a minimum intensity of 5000 counts. Full scans were acquired at a resolution of 11,000 [FWHM] (*m*/*z* 922). In negative ion mode, parameters were identical to positive mode. TFA anion, C_2_O_2_F_3_ (NH_4_) (*m*/*z* 112.9867) and HP 0921 formate (*m*/*z* 966.0007) were used as lock mass. Data analysis was performed with MassHunter^®^ (Agilent Technologies, Les Ulis, France), Qualitative Analysis B.07.00. Average MS/MS spectrum for all collision energies were extracted with a positive MS/MS TIC threshold of 10,000 and a negative MS/MS TIC threshold of 1000, following the workflow “find compound by AutoMS/MS”, with a mass match tolerance of 0.05 *m*/*z* and a retention time window of 0.25 min, prior to exportation in mgf format. The MS/MS spectra generated by this workflow for spongialactams were inspected manually and clear spectra were selected prior to mgf exportation. 

### 3.4. NMR Analysis

All NMR experiments were recorded on Avance III HD 400 MHz and 600 MHz spectrometers (Bruker) equipped with a BBFO Plus Smartprobe and a triple resonance TCI cryoprobe, respectively. 

### 3.5. Molecular Networking and Manual Dereplication

#### LC–MS/MS data were converted into mgf files using MassHunter® software,(Qualitative Analysis B.07.00, Agilent Technologies, Les Ulis, France). Converted data files were subjected to online GNPS workflow (http://gnps.ucsd.edu). Consensus spectra were generated through MS-Cluster with a parent ion mass tolerance of 0.5 Da and a fragment ion mass tolerance of 0.5 Da, with a minimum of 2 spectra. The networks were generated using the following settings: min pair cos: 0.7, minimum matched fragment ion: 6, network topK: 10. Resulting networks were visualized using Cytoscape 3.2.0. The preferred layout was applied. Node colors were mapped based on the source files of MS/MS spectra. The edge thickness attribute was defined to reflect cosine similarity scores, with thicker lines indicating higher similarity. Manual dereplication was performed using the MarinLit database (http://pubs.rsc.org/marinlit).

### 3.6. Multivariate Data Analysis

The profile-mode LC–MS data were processed using XCMS Online version 2.01.00 (https://xcmsonline.scripps.edu/index.php) [17,18]. Peaks were detected with the matched-filter method, using a maximal tolerated *m*/*z* deviation in consecutive scans of 30 ppm, and minimal and maximal peak widths of 10 and 60 s, respectively. The multivariate matrix generated consisted of 30 samples and 297 peaks, each characterized by a *m*/*z* ratio and a retention time. Isotopic peaks and adducts were annotated using the package CAMERA [19] implemented on XCMS Online, with automatic assignment of isotopes and adducts. The peak correlation based annotation obtained with CAMERA, which highlights and annotates co-eluted peaks, proposed that the 297 peaks detected correspond to at least 86 individual metabolites. The multivariate matrix was treated using the freely available R environment version 3.1.1 (www.r-project.org). PCA, PLS-DA and sPLS-DA were carried out using R package MixOmics [51]. The multivariate analyses were performed on mean-centered data. sPLS-DA was carried out for 3 components with 10 variables kept on the first component and 30 variables kept on components 2 and 3. Within the variables selected, the compounds with a maximum area of 5000 counts and not eluted in the void volume were further picked out.

### 3.7. Compound Isolation and Characterization

The sponge extracts Riou 2011 and 2012 were combined (1.71 g) and subjected to successive RP-HPLC using an Ultimate 3000 HPLC (Thermo Scientific) system (C18 Luna Phenomenex column, 250 × 4.6 mm, 100 Å, 5 µm, gradient elution 1 mL/min, CH_3_CN/H_2_O with isocratic 0.1% HCOOH, wavelength: 226 nm) to yield compounds **1** (2.7 mg) and **11** (1.8 mg) at retention times 24.0 and 26.5 min, respectively. Aliquot of the combined CH_2_Cl_2_/MeOH extracts of Riou 2011 and 2012 (40 mg) containing the highest amount of compound **2** according to box plot analysis (Figure 2) were dissolved in MeOH, then centrifugated (5000 g, 5 min). The same process was applied to other CH_2_Cl_2_/MeOH extracts (Cortiou 2011 and Riou 2013, total 180 mg) in order to isolate compounds (**12a**) and (**12b**). The methanolic supernatants were subjected to successive fractionations on a Kinetex Biphenyl column (250 × 4.6 mm, 100 Å, 5 µm, Phenomenex) then on a C18 Luna column (250 × 4.6 mm, 100 Å, 5 µm, Phenomenex) in the gradient elution 1 mL/min 30–85% CH_3_CN/H_2_O with an isocratic 0.1% HCOOH over 32 min, wavelength 210 nm. Furofficin (**2**), spongialactam A (**12a**) and spongialactam B (**12b**) were obtained in these experimental conditions at retention times of 20.3 (0.1 mg), 19.0 (0.4 mg) and 19.4 (0.3 mg) min, respectively.

*Demethylfurospongin-4* (**1**): Colorless oil; UV (EtOH) λ_max_ (ε) 206 (12700) nm; IR (NaCl disk) ν_max_ 2924, 2854, 1685, 1558 cm^−1^; NMR data see Supplementary data Table S4, Figures S5 and S6; (+) HRESIMS *m*/*z* 415.2494 [M + H]^+^ (calcd. 415.2479 for C_25_H_35_O_5_). 

*Furofficin* (**2**): White amorphous solid. [α]^25^_D_ +6 (*c* 0.09 MeOH). NMR data see Table 2 and Figures S14–S18; (−) HRESIMS *m/z* 431.2442 [M − H]^−^ (calcd for C_25_H_35_O_6_, 431.2439). 

*Furospongin-1* (**11**): Colorless oil. NMR data see Supplementary data Table S5, Figures S12 and S13; (+) HRESIMS *m/z* 331.2 [M + H]^+^ (calcd. 331.2268 for C_21_H_31_O_3_). 

*Spongialactam A* (**12a**): White amorphous solid. [α]^25^_D_ +9 (*c* 0.07 MeOH). NMR data see Table 2; and Figures S19–S23; (−) HRESIMS *m/z* 402.2280 [M − H]^−^ (calcd for C_23_H_32_NO_5_, 402.2286). MS/MS data see 5c and 7, Tables S7 and S8.

*Spongialactam B* (**12b**): White amorphous solid. [α]^25^_D_ +7 (*c* 0.13 MeOH). NMR data see Table 2 and and Figures S24–S28; (−) HRESIMS *m/z* 402.2288 [M − H]^−^ (calcd for C_23_H_32_NO_5_, 402.2286). MS/MS data see 5d,7; Tables S9 and S10.

## 4. Conclusions

*S. officinalis* secondary metabolite profiles obtained from a set of samples collected at two sites over three years confirmed the richness of this sponge in furanoterpenes and revealed a spatial and temporal variability in the composition of this family. Although furanoterpenes from *Spongia* sp. have been extensively explored, LC–MS/MS together with molecular networking permitted to delineate several new representatives of this family. To the best of our knowledge, our study constitutes the first analysis of collision induced dissociation of furanoterpene ions generated by electrospray ionization. Interpretation of the fragmentation data permitted to propose putative structures based on dereplication (six compounds), but unambiguous determination of the structural formula was hindered due to ambiguities in the localization of hydroxyl groups and/or unsaturations. Isolation and structural analysis permitted to identify five compounds, including a new furanosesterterpene and two new furanoterpene derivatives with a glycinyl lactam moiety. 

The chemical profiles of samples collected at two sites differentially impacted by anthropogenic pollution revealed the detection of specific metabolites including marine pollutants at certain time points, such as the synthetic surfactant C_11_ DEA. LC–MS/MS based metabolomics on marine bioindicator organisms with either targeted or untargeted approaches thus appear to be a promising method, complementary to ecotoxicological studies, to trace biomarkers of marine pollution (metabolomics targeted on specific pollutants) or identify pollutants in environmental samples (untargeted metabolomics).

## Figures and Tables

**Table 1:** Compounds associated with the clustering of the metabolomic profiles of *S. officinalis* extracts: retention times, monoisotopic *m*/*z* of the [M + H]^+^ species and identification. New compounds are indicated in bold.

Compound	Retention Time (min)	*m*/*z*	Identification/Annotation	Reference
**1**	39.1	415.2	Demethylfurospongin-4	[12]
**2**	32.5	433.3	Furofficin, new compound	this study
**3**	43.3	445.3	Unknown	-
**4**	36.3	568.4	Unknown	-
**5**	33.1	288.3	Coconut C_11_ diethanolamide ^(a)^	[22]
**6**	33.4	360.3	Furanoterpene, C_22_H_33_NO_3_	-
**7**	31.0	329.2	Dihydrofurospongin-2 ^(a)^	[23]
**8**	42.1	329.2	Furanoterpene, C_21_H_28_O_3_	-
**9**	32.8	363.2	Isomers of γ-hydroxy-α,β-butenolide or β-γ-epoxy butenolide furospongin-1 ^(a)^	[24]
**10**	35.9	363.2	Furanoterpene, C_21_H_30_O_5_	-
**11**	41.5	331.2	Furospongin-1	[9,23,25]
**12**	31.8	404.2	Two new isomers: **Spongialactam A** (**12a**) and **Spongialactam B** (**12b**)	this study
**13**	36.7	377.2	Irciformonin B ^(a)^	-
**14**	37.0	460.3	Furanoterpene, C_27_H_41_NO_5_	-
**15**	29.8	448.3	Furanoterpene, C_28_H_33_NO_4_	-

^(a)^ Annotation proposed from LC–MS/MS-based dereplication.

**Table 2:** NMR spectroscopic data (600 MHz, methanol-*d4*) for furofficin (**2**), spongialactam A (**12a**) and spongialactam B (**12b**).

	Furofficin (2)				Spongialactam A (12a)				Spongialactam B (12b)			
	δ_C_^a^	δ_H_ (mult, *J* in Hz)	COSY	HMBC (^1^H͢͢-^13^C)	δ_C_^a^	δ_H_ (mult, *J* in Hz)	COSY	HMBC (^1^H͢͢-^13^C)	δ_C_^a^	δ_H_ (mult, *J* in Hz)	COSY	HMBC (^1^H͢͢-^13^C)
**1**	143.6	7.38 (dd, 1.7 ; 1.7)	2, 4	2, 3, 4	143.7	7.37 (t, 1.7)	2,4	3, 4	143.7	7.37 (t, 1.7)	2, 4	2, 3, 4
**2**	111.6	6.30 (dd, 1.7 ; 0.7)	1, 4	1, 3, 4	112,0	6.31 (bd 0.9)	1, 4	1, 4	111.8	6.29 (bd, 0.9)	1, 4	1, 4
**3**	126.0	-	-	-	126.05*	-	-	-	126.4*	-	-	-
**4**	139.8	7.27 (dd, 1.5 ; 0.9)	1, 2, 5	2, 3, 1	140.0	7.25 (quint, 0.8)	1, 2, 5	1, 2	139.9	7.25 (m)	1, 2, 5	1, 2, 3
**5**	25.9	2.42 (brt, 7.4)	4, 6	2, 3, 4, 6, 7	25.8	2.46 (t, 7.4)	4, 6	2, 3, 6, 7	25.7	2.40 (t, 7.4)	4, 6	2, 3, 4, 6, 7
**6**	25.3	1.60 (m)	5, 7	5, 7	29.5	2.27 (m)	5, 7	7	28.5	1.59 (m)	5, 7	7
**7a**	41.8	1.48 (m)	6	8, 9	127.8	5.21 (bt, 7.1)	6	9, 10	38.5	1.21 (m)	6, 7b	-
**7b**										1.32 (m)	6, 7a, 9	-
**8**	72.8	-	-	-	133.9*	-	-	-	29.9	1.71 (m)	9, 10a	-
**9**	26.5	1.13 (s)	-	7, 8, 10	16.5	1.61 (brs)	7	7, 8, 10	19.5	0.89 (d, 6.6)	7b, 8	7, 8, 10
**10a**	42.3	1.45 (m)	11	7, 8, 9, 11, 12	50.0	2.05 (dd, 13.5 ; 6.3)	10b, 11	7, 8, 9, 11	45.2	1.14 (ddd, 13.8, 9.8, 3.5)	10b, 11	-
**10b**						2.15 (dd, 13.1 ; 6.8)	10a, 11	7, 8, 9, 11, 12		1.37 (ddd, 14.3, 9.5, 3.9)	10a, 11	-
**11**	23.2	2.00 (m)	10, 12	10, 12, 13	68.1	3.76 (m)	10a, 10b, 12a	-	68.0	3.77 (m)	10, 12	11
**12a**	125.4	5.15 (td, 7.1 ; 1.0)	11, 14	11, 14, 15	45.4	1.13 (ddd, 14.1 ; 10.0 ; 3.4)	11, 12b	-	49.7	2.06 (dd, 13.3, 6.3)	11, 12b	11, 13, 14, 15
**12b**						1.36 (ddd, 13.9 ; 9.9 ; 3.9)	12a, 13	-		2.14 (dd, 13.4, 7.6)	11, 12a	10, 11, 13, 14, 15
**13**	135.5^b^	-	-	-	30.1	1.72 (m)	12b, 14	-	134.2^b^	-	-	-
**14**	15.6	1.61 (brs)	12	12, 13, 15	19.5	0.88 (d, 6.6)	13	12, 13, 15	16.1	1.63 (brs)	15, 16	12, 13, 15
**15a**	40.4	2.05 (m)	16	12, 13, 14, 16, 17	38.8	1.24 (m)	15b, 13	-	127.2	5.21 (m)	14, 16	-
**15b**						1.34 (m)	15a	-				
**16**	28.8	2.35 (m)	15, 17	13, 15, 17, 18	26.2	1.58 (m)	17	-	27.0	2.29 (m)	15	13, 15, 17
**17**	129.5	5.31 (t)	16	15, 16, 19, 20	27.0	2.24 (m)	16, 20, 21	18, 20	26.8	2.30 (m)	20, 21	16, 18, 19, 20
**18**	140.2^b^	-	-	-	140.1^b^	-	-	-	138.2^b^	-	-	-
**19**	177.5^b^	-	-	-	173.8^b^	-	-	-	173.8^b^	-	-	-
**20**	35.3	2.31 (m)	21, 22	21	137.7	6.83 (m)	17, 21	19, 21	137.9	6.83 (m)	17, 21	19, 21
**21**	28.9	2.29 (m)	20, 22, 24	20	53.0	4.06 (d, 1.7)	-	18, 20	53.0	4.05 (d, 1.6)	17, 20	18, 20
**22**	139.2	6.61 (td, 8.7 ; 1.6)	20, 21, 24	24	46.7	4.05 (brs)	-	19, 21, 23	46.9	4.03 (brs)	-	19, 21, 23
**23**	129.9^b^	-	-	-	175.5^b^	-	-	-	175.9^b^	-	-	-
**24**	13.0	1.81 (brs)	21, 22	22, 23, 25								
**25**	174.9^b^	-	-	-								

^a^ ^13^C assignments supported by HSQC experiment. ^b^ ^13^C assignments supported by HMBC experiment.

## Supplementary Materials

The following are available online at www.mdpi.com/2218-1989/7/2/27/s1, Figure S1: Overlay of the total ion chromatograms of the extracts of *S. officinalis*, Figure S2: PCA analysis of not-normalized versus normalized dataset, Figure S3: VIP scores on the first three components of the PLS-DA, Figure S4: Score and loading plots of sPLS-DA, Figure S5: ^1^H NMR spectrum of demethylfurospongin-4 (**1**), Figure S6: ^13^C NMR spectrum of demethylfurospongin-4 (**1**), Figure S7: LC–MS/MS spectra of the of compounds **3**, **4** and **5**, Figure S8: LC–MS/MS spectra of compounds **6**, **13**, **14** and **15**, Figure S9: LC–MS/MS spectra of the compounds **7** and **8**, Figure S10: LC–MS/MS spectra of compounds **9** and **10**, Figure S11: LC–MS/MS spectrum of compound **11**, Figure S12. ^1^H NMR spectrum of furospongin-1 (**11**), Figure S13: DEPT NMR spectrum of furospongin-1 (**11**), Figure S14: ^1^H NMR spectrum of furofficin (**2**), Figure S15: ^1^H-^1^H COSY spectrum of furofficin (**2**), Figure S16: HSQC spectrum of furofficin (**2**), Figure S17: HMBC spectrum of furofficin (**2**), Figure S18: NOESY spectrum of furofficin (**2**), Figure S19: ^1^H NMR spectrum of spongialactam A (**12a**), Figure S20: ^1^H-^1^H COSY spectrum of spongialactam A (**12a**), Figure S21: HSQC spectrum of spongialactam A (**12a**), Figure S22: HMBC spectrum of spongialactam A (**12a**), Figure S23: NOESY spectrum of spongialactam A (**12a**), Figure S24: ^1^H NMR spectrum of spongialactam B (**12b**), Figure S25: ^1^H-^1^H COSY spectrum of spongialactam B (**12b**), Figure S26: HSQC spectrum of spongialactam B (**12b**), Figure S27: HMBC spectrum of spongialactam B (**12b**), Figure S28: NOESY spectrum of spongialactam B (**12b**), Table S1: Compound annotation of each LC–MS peak detected. Peaks are named MxTy, where x denotes the nominal *m*/*z* ratio and y indicates the nominal retention time, Table S2: Detail of the peaks detected by LC–MS for compounds **1** to **15**, Table S3: Positive-mode ion-dependent automatically-acquired MS/MS spectra of the detected compounds, Table S4: ^1^H and ^13^C NMR data of demethylfurospongin-4 (**1**), Table S5: ^1^H and ^13^C NMR data of furospongin-1 (**11**), Table S6: Assignment of selected nodes from the furanoterpene cluster observed in the molecular network, Table S7: Product ions from spongialactam A (**12a**) obtained by LC–MS/MS in positive mode, Table S8: Product ions from spongialactam A (**12a**) obtained in negative mode, Table S9: Product ions from spongialactam B (**12b**) obtained by LC–MS/MS by LC–MS/MS in positive mode, Table S10: Product ions from spongialactam B (**12b**) obtained by LC–MS/MS in positive mode.
