# Supplementary material for: Furanoterpene Diversity and Variability in the Marine Sponge Spongia officinalis, from Untargeted LC–MS/MS Metabolomic Profiling to Furanolactam Derivatives
Source: Metabolites. 2017 Jun 13;7(2):27. doi: 10.3390/metabo7020027 (PMC5487998; doi:10.3390/metabo7020027)
Supplement: Supplementary file 1 [file metabolites-07-00027-s001.pdf]

Furanoterpene diversity and variability in the metal polluted sponge *Spongia officinalis*, from untargeted LC-MS/MS metabolic profiling to furanolactam derivatives

Cléa Bauvais,[†,‡,#] Natacha Bonneau,[†,#] Alain Blond,[†] Thierry Pérez,[§] Marie-Lise Bourguet-Kondracki,[†,*] Séverine Zirah[†,*]

†: MCAM UMR 7245, Muséum National d'Histoire Naturelle, Centre National de la Recherche Scientifique, Sorbonne Universités, Paris, France

‡: Université Pierre et Marie Curie, Sorbonne Universités, Paris, France

§: Institut méditerranéen de Biodiversité et d'Ecologie marine et continentale. CNRS UMR 7263 / IRD 237/ Aix-Marseille Université / Avignon Université

# These authors contributed equally to the work

* Corresponding authors

Supporting information

Table S1. Compound annotation of each LC-MS peak detected. Peaks are named MxTy, where x denotes the nominal *m*/*z* ratio and y indicates the nominal retention time. The columns mzmed and rtmed provide the median *m*/*z* and retention time, respectively. The fold change and p-value corresponds to the xcmsOnline comparison between Riou and Cortiou (t-test). Column npeaks gives the number of samples where peaks were detected. Isotope, adduct and peak group columns provide the peak annotation obtained with CAMERA (the 297 peaks are assigned to 86 peak groups based on rule based annotation of isotopes and adducts, and extracted ion chromatograms correlation). Compounds **1** to **15** are highlighted in blue and annotated in the column assignment.

	name	mzmed	rtmed	fold	pvalue	npeaks	maxint	isotopes	adduct	Peak group	assignment
1	M411T31	411.23	31.21	3.5	5.5E-06	8	93			55	
2	M416T32	416.26	32.44	6.3	8.3E-06	21	659	[27][M]+	[M+H]+ 415.252	9	
3	M432T39	432.29	39.09	4.8	9.3E-06	19	218		[M+H+NH3]+ 414.244	7	
4	M398T32	398.25	32.43	3.4	2.5E-05	8	84		[M+H-H20]+ 415.252	9	
5	M434T32	434.27	32.46	8.0	2.6E-05	11	224	[29][M+1]+		9	
6	M417T32	417.26	32.42	2.7	2.7E-05	11	101	[27][M+1]+		9	
7	M450T33	450.29	32.55	4.4	3.0E-05	10	86			48	
8	M727T33	727.45	33.05	2.3	4.1E-05	16	147	[44][M+2]+		18	
9	M893T31	893.49	31.16	5.3	4.4E-05	8	38			68	
10	M415T39_1	415.25	39.11	5.1	5.8E-05	17	2319	[26][M]+	[M+H]+ 414.244	7	**(1)**
11	M397T32	397.25	32.44	6.3	6.3E-05	11	294			9	
12	M447T31	447.25	31.29	5.9	6.4E-05	11	207			55	
13	M861T33	860.53	32.58	3.5	8.4E-05	13	185	[56][M+1]+		48	
14	M415T32_1	415.25	32.45	11.9	1.1E-04	17	2248		[M+H-H20]+ 432.256	9	
15	M860T33	859.53	32.60	4.4	1.5E-04	18	362	[56][M]+	[M+K]+ 820.563<br>[M+Na]+ 836.537<br>[M+H]+ 858.519	48	
16	M832T39	831.51	39.13	6.3	1.9E-04	12	94	[53][M+2]+		7	
17	M416T39	416.26	39.16	3.2	1.9E-04	15	778	[26][M+1]+		7	
18	M433T32	433.27	32.49	10.3	2.9E-04	18	833	[29][M]+	[M+H]+ 432.256	9	**(2)**
19	M397T39	397.25	39.09	2.7	3.6E-04	15	228		[M+H-H20]+ 414.244	7	
20	M831T39	830.50	39.13	11.9	3.7E-04	12	380	[53][M+1]+		7	
21	M877T33	876.55	32.58	2.9	3.9E-04	12	103		[M+K+NH3]+ 820.563<br>[M+Na+NH3]+ 836.537<br>[M+H+NH3]+ 858.519	48	
22	M415T32_2	415.38	32.45	6.6	4.1E-04	8	76			9	
23	M829T39	829.49	39.12	14.9	4.9E-04	12	718	[53][M]+	[2M+H]+ 414.244	7	

	name	mzmed	rtmed	fold	pvalue	npeaks	maxint	isotopes	adduct	Peak group	assignment
24	M464T31	464.27	31.35	3.6	5.7E-04	10	81			78	
25	M1245T39	1244.74	39.16	9.4	6.5E-04	8	76			7	
26	M350T38	350.14	37.84	2.6	6.6E-04	11	123			74	
27	M884T32	883.55	32.42	7.0	7.5E-04	10	138	[58][M+1]+		9	
28	M289T35	289.23	35.07	1.7	8.5E-04	17	72			80	
29	M847T39	846.53	39.14	2.6	9.9E-04	13	86			7	
30	M465T26	465.26	26.12	7.6	1.0E-03	10	143			71	
31	M813T33	812.50	32.76	3.2	1.3E-03	11	68	[51][M]+	[M+K+NH3]+ 756.516 [M+Na+NH3]+ 772.49 [M+H+NH3]+ 794.472	21	
32	M883T32	882.54	32.43	10.0	1.4E-03	11	262	[58][M]+		9	
33	M757T33	757.46	32.96	2.5	1.5E-03	17	201			61	
34	M866T32	865.51	32.45	52.4	1.7E-03	8	1795	[57][M]+	[2M+H]+ 432.256	9	
35	M415T39_2	415.38	39.10	3.3	1.7E-03	9	75			7	
36	M867T32	866.52	32.46	34.3	1.7E-03	10	943	[57][M+1]+		9	
37	M488T30	488.27	30.44	7.0	1.8E-03	13	286			54	
38	M849T32	848.51	32.43	3.9	1.9E-03	10	111	[55][M+1]+		9	
39	M885T32	884.55	32.42	3.1	2.1E-03	10	46	[58][M+2]+		9	
40	M659T41	659.44	41.33	1.5	2.4E-03	21	343	[38][M]+		51	
41	M868T32	867.53	32.45	6.6	2.5E-03	10	225	[57][M+2]+		9	
42	M743T33	743.47	33.06	1.7	3.2E-03	20	130	[45][M+1]+		18	
43	M398T45	398.34	44.63	1.7	3.4E-03	14	129			13	
44	M848T32	847.51	32.43	5.4	3.6E-03	10	214	[55][M]+		9	
45	M795T33	795.48	32.72	3.6	3.6E-03	14	202		[M+K]+ 756.516 [M+Na]+ 772.49 [M+H]+ 794.472	21	
46	M814T33	813.51	32.81	2.1	4.6E-03	8	48	[51][M+1]+		21	
47	M1299T32	1298.77	32.44	14.1	5.7E-03	8	201	[60][M+1]+		9	
48	M270T41	270.29	41.25	2.0	5.8E-03	8	58			26	
49	M1298T32	1297.77	32.43	12.2	6.8E-03	10	250	[60][M]+	[3M+H]+ 432.256	9	
50	M796T33	796.48	32.72	2.6	6.8E-03	10	91			29	
51	M348T41	348.26	41.48	1.7	8.2E-03	26	502	[12][M]+		38	
52	M388T39	388.27	38.91	2.6	8.3E-03	12	167	[23][M+1]+		1	
53	M369T40	369.14	40.38	2.0	8.8E-03	10	84			23	
54	M571T30	571.11	30.29	9.2	1.1E-02	24	168	[34][M]+		66	
55	M416T40	416.32	40.29	5.5	1.1E-02	10	553			23	

	name	mzmed	rtmed	fold	pvalue	npeaks	maxint	isotopes	adduct	Peak group	assignment
56	M572T30	572.11	30.30	9.1	1.1E-02	8	33	[34][M+1]+		66	
57	M732T31	732.46	31.15	2.1	1.2E-02	14	159			68	
58	M742T33	742.46	33.06	1.8	1.3E-02	21	291	[45][M]+	[M+H+NH3]+ 724.425	18	
59	M660T41	660.45	41.32	3.1	1.3E-02	20	160	[38][M+1]+		51	
60	M678T42	678.48	41.54	1.6	1.4E-02	10	55			3	
61	M724T34	724.43	33.52	2.5	1.5E-02	15	246	[43][M+1]+		33	
62	M644T42	644.45	41.56	1.3	1.8E-02	21	105	[37][M+1]+		3	
63	M417T39	417.26	39.09	1.7	1.9E-02	13	102	[26][M+2]+		7	
64	M387T39	387.26	38.90	2.0	2.0E-02	14	712	[23][M]+		1	
65	M371T45	371.30	44.63	4.8	2.0E-02	20	311			13	
66	M856T43	855.58	43.12	71.7	2.1E-02	15	427			4	
67	M890T43	889.59	43.07	26.2	2.1E-02	21	230			4	
68	M726T33	726.44	33.04	2.0	2.2E-02	19	615	[44][M+1]+		18	
69	M891T43	890.59	43.11	14.4	2.2E-02	19	131			4	
70	M457T44	457.34	43.61	6.9	2.3E-02	14	569			8	
71	M445T43	445.30	43.15	14.4	2.4E-02	26	765			4	**(3)**
72	M383T38	383.27	37.52	20.7	2.5E-02	12	261			44	
73	M385T43	385.28	43.02	9.5	2.6E-02	26	352	[21][M]+		11	
74	M778T32	778.50	32.34	4.2	2.6E-02	11	249			59	
75	M1088T33	1087.65	32.99	2.7	2.7E-02	15	226		[3M+2H]2+ 724.425	18	
76	M367T43	367.27	42.95	7.5	2.7E-02	9	74			79	
77	M725T33	725.43	33.04	2.1	2.7E-02	19	1444	[44][M]+	[M+H]+ 724.425	18	
78	M386T43	386.29	43.03	6.7	3.0E-02	11	93	[21][M+1]+		11	
79	M426T43	426.31	43.20	7.1	3.1E-02	13	161			67	
80	M723T34	723.42	33.52	2.6	3.2E-02	18	586	[43][M]+		33	
81	M443T37	443.29	37.46	18.8	3.4E-02	15	405			44	
82	M446T43	446.30	43.37	4.5	3.5E-02	24	383			5	
83	M536T40	536.38	40.15	11.3	3.6E-02	23	250			58	
84	M875T43	874.56	43.43	7.7	3.7E-02	15	319			5	
85	M461T38	461.30	38.02	17.0	3.8E-02	13	320			53	
86	M790T33	790.49	33.05	2.3	3.8E-02	20	311	[49][M+1]+		18	
87	M992T42	991.67	41.57	1.8	3.8E-02	18	191	[59][M]+		3	
88	M354T43	354.31	42.51	1.5	3.9E-02	12	71			82	

	name	mzmed	rtmed	fold	pvalue	npeaks	maxint	isotopes	adduct	Peak group	assignment
89	M395T37	395.27	36.68	1.4	3.9E-02	20	159	[24][M+1]+		19	
90	M568T36	568.35	36.15	6.3	4.2E-02	14	192			25	**(4)**
91	M522T39	522.37	38.66	3.8	4.3E-02	20	62			83	
92	M466T36	466.30	35.80	4.1	4.5E-02	11	89			32	
93	M789T33	789.48	33.07	2.4	4.7E-02	20	733	[49][M]+	[M+K]+ 750.522 [M+Na]+ 766.496 [M+H]+ 788.478	18	
94	M349T41	349.27	41.42	1.4	4.8E-02	22	116	[12][M+1]+		38	
95	M723T45	722.57	44.63	1.6	4.8E-02	21	164	[42][M]+		13	
96	M370T45	370.30	44.63	4.3	4.9E-02	21	118		[M+H+NH3]+ 352.267	13	
97	M480T37	480.32	37.26	2.4	5.1E-02	22	179			52	
98	M858T43	857.53	43.48	4.0	5.1E-02	13	1168			5	
99	M570T37	570.37	37.09	5.5	5.2E-02	20	265			56	
100	M754T37	754.47	36.73	2.3	5.2E-02	15	342	[46][M+1]+		30	
101	M319T35	319.09	34.98	3.1	5.2E-02	13	58			84	
102	M993T42	992.68	41.56	1.7	5.3E-02	18	116	[59][M+1]+		3	
103	M784T31	784.47	31.24	1.8	5.7E-02	14	173			55	
104	M643T42	643.45	41.58	1.3	5.8E-02	21	216	[37][M]+		3	
105	M255T32	255.15	31.69	1.4	6.2E-02	15	129	[1][M+1]+		12	
106	M342T39	342.19	38.86	28.6	6.3E-02	14	2825			1	
107	M341T39	341.18	38.86	13.5	6.3E-02	16	4872			1	
108	M536T41	536.38	41.03	2.9	6.9E-02	15	180			64	
109	M753T37	753.47	36.73	3.0	6.9E-02	19	642	[46][M]+		30	
110	M689T36	689.42	35.95	2.2	7.1E-02	17	551			17	
111	M603T38	603.42	37.62	2.8	7.1E-02	14	170			40	
112	M447T36	447.36	35.70	1.6	7.1E-02	17	361	[30][M]+		49	
113	M331T44	331.23	43.64	2.2	7.2E-02	11	2258			8	
114	M369T43	369.29	43.00	108.1	7.3E-02	14	2079			11	
115	M625T42	625.44	41.58	1.3	7.5E-02	18	49			3	
116	M464T38	464.39	38.41	1.7	7.6E-02	20	161	[31][M+1]+		34	
117	M807T33	806.51	33.02	1.8	8.0E-02	14	160		[M+K+NH3]+ 750.522 [M+Na+NH3]+ 766.496 [M+H+NH3]+ 788.478	18	
118	M837T32	836.51	32.32	1.6	8.6E-02	9	76			59	
119	M298T5	298.11	4.71	2.3	8.7E-02	22	168			65	
120	M361T43	361.27	43.02	3.2	8.7E-	13	92	[15][M+1]+		11	

	name	mzmed	rtmed	fold	pvalue	npeaks	maxint	isotopes	adduct	Peak group	assignment
					02						
121	M647T38	647.45	37.58	2.6	8.8E-02	17	146			57	
122	M559T38	559.39	37.67	2.6	9.0E-02	19	196			40	
123	M288T34	288.30	34.08	4.2	9.1E-02	14	426			42	
124	M273T38	273.19	38.27	1.7	9.1E-02	28	526	[3][M]+		35	
125	M360T43	360.26	43.02	3.6	9.2E-02	16	350	[15][M]+		11	
126	M771T37	771.50	36.69	1.5	9.7E-02	14	98	[48][M+1]+		19	
127	M600T43	600.48	42.79	1.7	1.0E-01	13	93			77	
128	M488T44	488.32	44.35	2.2	1.0E-01	15	142	[32][M+1]+		45	
129	M805T32	805.47	31.76	2.2	1.0E-01	13	260			12	
130	M411T41	411.22	41.14	219.8	1.0E-01	11	4444			2	
131	M841T43	840.56	43.29	1.8	1.0E-01	13	90	[54][M+1]+		69	
132	M840T43	839.56	43.29	1.7	0.11	14	154	[54][M]+		69	
133	M405T32	405.25	31.76	4.4	0.11	20	593	[25][M+1]+		12	
134	M339T42	339.26	42.26	1.4	0.11	16	87			10	
135	M324T39	324.23	38.51	2.7	0.11	16	231			47	
136	M300T42	300.30	42.06	2.0	0.11	20	127			6	
137	M460T37	460.31	37.21	1.6	0.12	14	320			52	**(14)**
138	M463T38	463.39	38.41	1.9	0.12	21	529	[31][M]+		34	
139	M274T38	274.20	38.25	1.7	0.12	16	128	[3][M+1]+		35	
140	M343T43	343.23	43.09	2.4	0.12	22	3545	[11][M]+		4	
141	M329T41	329.22	41.21	2.0	0.12	15	859		[M+Na+K]2+ 596.487	26	
142	M267T39	267.28	39.08	2.2	0.13	14	156			7	
143	M505T44	505.33	44.35	1.4	0.13	21	131			45	
144	M448T30	448.26	29.83	2.0	0.13	13	518			36	**(15)**
145	M355T45	355.29	44.63	1.2	0.13	16	67	[13][M+2]+		13	
146	M364T33	364.23	32.82	1.4	0.14	20	280	[17][M+1]+		21	
147	M363T33	363.22	32.84	1.4	0.14	21	1219	[17][M]+		21	**(9)**
148	M429T43	429.26	43.45	1.7	0.14	15	2853	[28][M]+		5	
149	M378T37	378.24	36.64	1.5	0.14	18	440			19	
150	M657T31	657.42	30.98	1.8	0.15	17	478			20	
151	M288T33	288.26	33.18	2.7	0.15	16	1503			16	**(5)**
152	M289T33	289.27	33.15	2.6	0.15	13	263			16	
153	M608T17	608.40	16.54	2.0	0.16	17	41			85	
154	M360T37	360.23	36.64	1.3	0.16	21	238	[14][M+1]+		19	
155	M770T37	770.49	36.69	1.6	0.16	21	187	[48][M]+		19	
156	M291T36	291.26	35.55	2.0	0.16	29	238			31	
157	M345T33	345.21	33.18	1.4	0.16	21	1021			16	
158	M388T43	388.30	43.05	2.1	0.16	15	176			4	
159	M283T43	283.27	42.64	2.6	0.17	15	1554			15	
160	M341T44	341.28	44.38	1.8	0.17	16	336			45	
161	M577T38	577.43	37.66	2.0	0.18	16	144	[35][M+1]+		40	
162	M421T32	421.28	31.75	1.4	0.19	17	101			12	
163	M808T32	808.49	31.75	1.8	0.19	15	1024	[50][M+1]+		12	
164	M335T36	335.29	35.64	1.8	0.19	19	180			31	
165	M488T38	488.37	37.75	2.0	0.19	19	129			73	
166	M809T32	809.49	31.74	1.7	0.20	15	215	[50][M+2]+		12	
167	M377T37	377.24	36.66	1.5	0.20	21	1244			19	**(13)**
168	M387T32	387.25	31.74	1.7	0.20	15	220	[22][M+1]+		12	

	name	mzmed	rtmed	fold	pvalue	npeaks	maxint	isotopes	adduct	Peak group	assignment
169	M484T41	484.32	40.84	1.5	0.20	17	198			27	
170	M496T40	496.35	40.23	1.4	0.20	21	387			46	
171	M652T17	652.42	17.15	1.8	0.20	20	34			86	
172	M641T42	641.43	42.17	1.4	0.21	14	143			62	
173	M435T36	435.36	35.59	2.4	0.21	16	623			31	
174	M325T43	325.23	43.09	1.9	0.21	19	309			4	
175	M381T36	381.25	35.97	1.2	0.21	15	205	[20][M+1]+		17	
176	M333T42	333.24	41.53	1.4	0.22	21	116	[9][M+2]+		3	
177	M665T38	665.48	37.60	1.8	0.22	20	121	[40][M+1]+		40	
178	M621T38	621.45	37.63	1.8	0.22	19	143	[36][M+1]+		40	
179	M871T33	870.57	32.64	1.8	0.22	12	263		[M+K+NH3]+ 814.574<br>[M+Na+NH3]+ 830.548<br>[M+H+NH3]+ 852.529	29	
180	M250T45	250.10	44.69	2.0	0.23	11	122			13	
181	M361T34	361.21	33.53	1.5	0.23	16	392			24	
182	M369T32	369.24	31.74	1.6	0.23	13	58			12	
183	M327T33	327.20	32.78	1.2	0.23	19	318			21	
184	M753T38	752.53	37.54	1.8	0.23	19	156			57	
185	M343T41	343.24	40.85	2.3	0.23	19	756			27	
186	M576T38	576.42	37.66	1.8	0.24	20	485	[35][M]+		40	
187	M620T38	620.45	37.64	1.8	0.24	22	453	[36][M]+		40	
188	M359T37	359.23	36.65	1.3	0.24	21	995	[14][M]+		19	
189	M344T43	344.24	43.09	1.9	0.24	23	1440	[11][M+1]+		4	
190	M664T38	664.48	37.59	1.8	0.24	21	350	[40][M]+		40	
191	M709T38	708.50	37.57	1.8	0.25	19	254			57	
192	M336T45	336.27	44.63	1.2	0.26	19	60	[10][M+1]+		13	
193	M285T36	285.29	35.76	1.9	0.26	16	138			32	
194	M279T39	279.17	38.52	1.4	0.27	20	383			47	
195	M296T39	296.20	38.51	1.9	0.27	16	60			47	
196	M854T33	853.54	32.68	1.9	0.27	12	669		[M+K]+ 814.574<br>[M+Na]+ 830.548<br>[M+H]+ 852.529	29	
197	M807T32	807.48	31.74	1.7	0.27	15	2046	[50][M]+		12	
198	M430T43	430.27	43.44	1.4	0.28	19	1042	[28][M+1]+		5	
199	M508T36	508.35	36.19	1.5	0.28	15	138			25	
200	M504T44	504.34	44.33	1.3	0.29	21	106			45	
201	M403T42	403.24	42.13	1.6	0.29	22	143			62	
202	M394T37	394.27	36.69	1.3	0.29	21	720	[24][M]+		19	
203	M258T3	258.14	3.26	1.6	0.29	24	55			50	
204	M827T32	826.52	31.76	1.4	0.30	14	70	[52][M+2]+		12	
205	M331T42_2	331.34	41.54	1.2	0.30	21	146			3	
206	M825T32	824.51	31.75	1.6	0.31	16	441	[52][M]+		12	
207	M301T3	301.15	3.21	1.6	0.31	28	117			76	
208	M826T32	825.52	31.74	1.5	0.32	15	214	[52][M+1]+		12	
209	M381T31	381.24	31.27	1.2	0.33	19	269			55	
210	M309T38	309.20	38.15	1.7	0.33	13	182			63	
211	M275T44	275.27	43.90	1.6	0.34	22	35			41	
212	M317T3	317.12	3.22	1.5	0.34	17	22			50	
213	M342T34	342.25	33.51	1.5	0.34	15	356			33	
214	M402T32	402.24	32.18	1.4	0.36	20	759			22	
215	M335T45	335.27	44.63	1.2	0.37	21	250	[10][M]+	[M+H-H20]+ 352.267	13	
216	M399T40	399.37	40.38	1.3	0.38	15	279			23	
217	M418T33	418.27	33.00	1.5	0.38	13	274		[M+Na]+ 395.278	18	
218	M755T43	754.52	43.12	1.3	0.38	19	319	[47][M+1]+		4	
219	M448T36	448.37	35.71	1.3	0.38	17	107	[30][M+1]+		49	
220	M382T31	382.27	30.89	1.3	0.38	18	186			20	

	name	mzmed	rtmed	fold	pvalue	npeaks	maxint	isotopes	adduct	Peak group	assignment
221	M254T32	254.15	31.69	1.4	0.39	21	823	[1][M]+		12	
222	M788T43	787.53	43.07	1.3	0.39	17	204			4	
223	M386T32	386.24	31.73	1.3	0.40	20	889	[22][M]+		12	
224	M302T3	302.17	3.23	1.4	0.42	21	50			50	
225	M269T4	269.11	3.94	1.2	0.42	13	99			37	
226	M358T34	358.25	34.02	1.4	0.43	13	238			42	
227	M313T42_2	313.33	41.56	1.2	0.43	21	141			3	
228	M291T3	291.08	3.27	1.2	0.43	17	58			50	
229	M280T44	280.27	43.62	2.3	0.44	14	180			8	
230	M296T42	296.22	41.52	1.2	0.44	18	51			3	
231	M330T42	330.22	42.31	1.2	0.45	20	565	[7][M+1]+		10	
232	M315T42	315.23	41.53	1.1	0.45	21	129	[5][M+1]+		3	
233	M261T6	261.15	5.98	1.3	0.45	15	134			72	
234	M493T42	493.34	41.56	1.1	0.45	21	109			3	
235	M330T31	330.22	30.96	1.2	0.46	17	280	[8][M+1]+		20	
236	M345T36	345.21	36.12	1.2	0.46	20	1060			25	
237	M404T32	404.25	31.77	1.3	0.47	21	1768	[25][M]+		12	**(12)**
238	M685T43	685.46	43.08	1.7	0.47	16	1187			4	
239	M267T40	267.28	40.38	1.4	0.48	30	534	[2][M]+	[M+H]+ 266.27	23	
240	M506T25	506.28	25.29	1.4	0.49	10	152			70	
241	M360T34	360.26	33.57	1.4	0.49	15	1109			24	**(6)**
242	M346T42	346.24	42.31	1.2	0.50	15	391			10	
243	M380T36	380.25	36.00	1.2	0.50	21	946	[20][M]+		17	
244	M532T38	532.39	37.69	1.4	0.50	17	179			40	
245	M329T42	329.21	42.31	1.2	0.51	21	2203	[7][M]+		10	**(8)**
246	M268T40	268.28	40.37	1.3	0.51	21	109	[2][M+1]+		23	
247	M454T37	454.34	37.29	1.4	0.51	16	123			75	
248	M268T4	268.11	3.93	1.2	0.52	18	508			37	
249	M265T42	265.26	42.11	1.3	0.53	23	198			62	
250	M380T33	380.25	33.10	1.1	0.54	19	334		[M+H-CH4]+ 395.278	18	
251	M368T32	368.23	31.74	1.2	0.54	19	200			12	
252	M724T45	723.57	44.64	1.1	0.55	20	110	[42][M+1]+		13	
253	M329T31	329.22	30.97	1.2	0.55	20	1228	[8][M]+		20	**(7)**
254	M363T36	363.22	35.94	1.2	0.55	21	1470	[16][M]+		17	**(10)**
255	M319T43	319.29	43.44	1.2	0.55	20	57			5	
256	M754T43	753.52	43.05	1.3	0.55	15	611	[47][M]+		4	
257	M487T44	487.32	44.35	1.2	0.57	21	400	[32][M]+		45	
258	M597T41	597.50	41.25	1.2	0.58	20	126		[M+H]+ 596.487	26	
259	M264T4	264.17	4.04	1.2	0.59	16	71			81	
260	M364T36	364.23	35.94	1.2	0.61	20	324	[16][M+1]+		17	
261	M297T40	297.25	40.40	1.2	0.63	29	129		[2M+Na+K]2+ 266.27	23	
262	M295T42	295.22	41.54	1.1	0.63	21	186			3	
263	M663T42	663.46	41.56	1.1	0.63	21	178	[39][M+2]+		3	
264	M661T42	661.44	41.56	1.2	0.64	21	2061	[39][M]+		3	
265	M749T32	749.48	31.91	1.3	0.66	14	1637			14	
266	M662T42	662.45	41.56	1.1	0.67	21	915	[39][M+1]+		3	
267	M614T42	614.50	41.93	1.1	0.71	17	203			39	
268	M535T40	534.54	40.37	1.2	0.73	24	436	[33][M+1]+		23	
269	M422T37	422.30	36.71	1.1	0.74	20	225			19	
270	M534T40	533.54	40.36	1.2	0.76	30	1113	[33][M]+	[2M+H]+ 266.27	23	
271	M329T43	329.22	43.14	1.1	0.76	21	1632	[6][M]+		4	
272	M313T42_1	313.21	41.57	1.1	0.76	24	4251			3	
273	M613T42	613.49	41.90	1.1	0.78	22	492			39	
274	M366T44	366.28	43.90	1.1	0.79	20	102	[18][M+1]+		41	
275	M365T44	365.28	43.90	1.1	0.80	21	442	[18][M]+		41	
276	M346T32	346.24	32.18	1.1	0.80	13	1153			22	
277	M566T42	565.53	42.08	1.1	0.81	17	185			62	

	name	mzmed	rtmed	fold	pvalue	npeaks	maxint	isotopes	adduct	Peak group	assignment
278	M707T45	706.55	44.64	1.1	0.81	20	168	[41][M+1]+		13	
279	M404T36	404.25	35.78	1.1	0.81	14	607			32	
280	M850T43	849.64	43.34	1.1	0.82	12	107			69	
281	M331T42_1	331.22	41.54	1.0	0.83	21	4156	[9][M]+		3	**(11)**
282	M328T32	328.23	32.09	1.1	0.84	13	707			28	
283	M367T46	367.29	45.56	1.0	0.88	21	420	[19][M]+		43	
284	M310T32	310.23	32.05	1.1	0.89	14	207			28	
285	M368T46	368.30	45.56	1.0	0.90	19	114	[19][M+1]+		43	
286	M353T45	353.27	44.63	1.0	0.91	22	1683	[13][M]+	[M+H]+ 352.267	13	
287	M354T45	354.28	44.63	1.0	0.92	21	426	[13][M+1]+		13	
288	M330T43	330.22	43.15	1.0	0.92	20	409	[6][M+1]+		4	
289	M332T42	332.23	41.57	1.0	0.93	26	1752	[9][M+1]+		3	
290	M440T30	440.30	29.83	1.0	0.94	17	320			36	
291	M284T42	284.27	42.03	1.0	0.94	28	650	[4][M+1]+		6	
292	M706T45	705.54	44.64	1.0	0.95	21	383	[41][M]+	[2M+H]+ 352.267	13	
293	M268T3	268.11	3.27	1.0	0.96	19	358			50	
294	M403T32	403.24	32.24	1.0	0.96	13	206			60	
295	M399T36	399.20	35.68	1.0	0.97	15	136			49	
296	M314T42	314.23	41.58	1.0	0.98	29	1829	[5][M]+		3	
297	M283T42	283.27	42.01	1.0	0.99	28	2725	[4][M]+		6	

Table S2. Detail of the peaks detected by LC-MS for compounds **1** to **15**.

Comp.	Retention time (min)	*m/z* detected in positive-ion mode	*m/z* detected in negative-ion mode	High-resolution measured *m/z*	Calculated *m/z*	Mass accuracy Δm (ppm)	Molecular formula
**1**	39.1	415.2 $[M+H]^+$<br>432.4 $[M+NH_4]^+$<br>829.5 $[2M+H]^+$<br>846.5 $[2M+NH_4]^+$	413.2 $[M-H]^-$	$[M+H]^+$<br>415.2462	$[M+H]^+$<br>415.2479	4.09	$C_{25}H_{35}O_5$
**2**	32.5	433.3 $[M+H]^+$<br>415.2 $[M+H-H_2O]^+$<br>450.3 $[M+NH_4]^+$<br>865.5 $[2M+H]^+$<br>882.5 $[2M+NH_4]^+$	431.2 $[M-H]^-$	$[M-H]^-$<br>431.2442	$[M-H]^-$<br>431.2439	0.70	$C_{25}H_{36}O_6$
**3**	43.3	445.3 $[M+H]^+$	443.3 $[M-H]^-$<br>489.3 $[M+HCOO]^-$	$[M+H]^+$<br>445.2934			
**4**	36.3	568.4 $[M+H]^+$<br>585.4 $[M+NH_4]^+$	nd	$[M+H]^+$<br>568.3439			
**5**	33.1	288.3 $[M+H]^+$	286.2 $[M-H]^-$	$[M+H]^+$<br>288.2524	$[M+H]^+$<br>288.2533	-3.12	$C_{16}H_{33}NO_3$
**6**	33.4	360.3 $[M+H]^+$<br>342.3 $[M+H-H_2O]^+$	nd	$[M+H]^+$<br>360.2536	$[M+H]^+$<br>360.2533	0.83	$C_{22}H_{33}NO_3$
**7**	31.0	329.2 $[M+H]^+$<br>311.2 $[M+H-H_2O]^+$<br>657.4 $[2M+H]^+$	nd	$[M+H]^+$<br>329.2094	$[M+H]^+$<br>329.2111	-5.16	$C_{21}H_{28}O_3$
**8**	42.1	329.2 $[M+H]^+$<br>346.2 $[M+NH_4]^+$<br>311.2 $[M+H-H_2O]^+$<br>657.4 $[2M+H]^+$	nd	$[M+H]^+$<br>329.2100	$[M+H]^+$<br>329.2111	-3.34	$C_{21}H_{28}O_3$
**9**	32.8	363.2 $[M+H]^+$<br>380.2 $[M+NH_4]^+$<br>345.2 $[M+H-H_2O]^+$<br>742.5 $[2M+NH_4]^+$	361.2 $[M-H]^-$	$[M+H]^+$<br>363.2145	$[M+H]^+$<br>363.2166	-5.78	$C_{21}H_{30}O_5$
**10**	35.9	363.2 $[M+H]^+$<br>380.2 $[M+NH_4]^+$<br>345.2 $[M+H-H_2O]^+$	nd	$[M+H]^+$<br>363.2146	$[M+H]^+$<br>363.2166	-5.51	$C_{21}H_{30}O_5$
**11**	41.5	331.2 $[M+H]^+$<br>348.3 $[M+NH_4]^+$<br>313.2 $[M+H-H_2O]^+$<br>661.5 $[2M+H]^+$<br>678.5 $[2M+NH_4]^+$	nd	$[M+H]^+$<br>331.2241	$[M+H]^+$<br>331.2268	-8.15	$C_{21}H_{30}O_3$
**12**	31.8	404.2 $[M+H]^+$<br>386.2 $[M+H-H_2O]^+$<br>421.3 $[M+NH_4]^+$<br>807.5 $[2M+H]^+$<br>824.5 $[2M+NH_4]^+$	nd	$[M+H]^+$<br>404.2415<br>(**12a**)<br>404.2416<br>(**12b**)	404.2431	-3.96 (**12a**)<br>-3.71 (**12b**)	$C_{23}H_{33}NO_5$
**13**	36.7	377.2 $[M+H]^+$<br>394.3 $[M+NH_4]^+$<br>359.2 $[M+H-H_2O]^+$<br>422.3 $[M+HCOOH]^+$<br>753.5 $[2M+H]^+$	nd	$[M+H]^+$<br>377.2306	$[M+H]^+$<br>377.2323	-4.51	$C_{22}H_{32}O_5$
**14**	37.0	460.3 $[M+H]^+$	nd	$[M+H]^+$<br>460.3039	$[M+H]^+$<br>460.3057	-3.91	$C_{27}H_{41}NO_5$
**15**	29.8	448.3 $[M+H]^+$<br>430.3 $[M+H-H_2O]^+$	nd	$[M+H]^+$<br>448.2472	$[M+H]^+$<br>448.2482	-2.23	$C_{28}H_{33}NO_4$

nd: not detected.

Table S3. Positive-mode ion-dependent automatically-acquired MS/MS spectra of the detected compounds (ESI-Qq-TOF Q-STAR instrument). Compounds **1** to **15** are highlighted in blue and annotated in the column assignment.

peak N°	name	mzmed	rtmed	peak group	assignement	MSMS
281	M331T42_1	331.22	41.54	3	**(11)**	yes
71	M445T43	445.30	43.15	4	**(3)**	yes
297	M283T42	283.27	42.01	6		yes
10	M415T39_1	415.25	39.11	7	**(1)**	yes
70	M457T44	457.34	43.61	8		yes
113	M331T44	331.23	43.64	8		yes
18	M433T32	433.27	32.49	9	**(2)**	yes
245	M329T42	329.21	42.31	10	**(8)**	yes
73	M385T43	385.28	43.02	11		yes
125	M360T43	360.26	43.02	11		yes
221	M254T32	254.15	31.69	12		yes
237	M404T32	404.25	31.77	12	**(12)**	yes
286	M353T45	353.27	44.63	13		yes
159	M283T43	283.27	42.64	15		yes
151	M288T33	288.26	33.18	16	**(5)**	yes
157	M345T33	345.21	33.18	16		yes
254	M363T36	363.22	35.94	17	**(10)**	yes
93	M789T33	789.48	33.07	18		yes
167	M377T37	377.24	36.66	19	**(13)**	yes
220	M382T31	382.27	30.89	20		yes
253	M329T31	329.22	30.97	20	**(7)**	yes
147	M363T33	363.22	32.84	21	**(9)**	yes
183	M327T33	327.20	32.78	21		yes
214	M402T32	402.24	32.18	22		yes
276	M346T32	346.24	32.18	22		yes
216	M399T40	399.37	40.38	23		yes
239	M267T40	267.28	40.38	23		yes
241	M360T34	360.26	33.57	24	**(6)**	yes
90	M568T36	568.35	36.15	25	**(4)**	yes
236	M345T36	345.21	36.12	25		yes
141	M329T41	329.22	41.21	26		yes
282	M328T32	328.23	32.09	28		yes
156	M291T36	291.26	35.55	31		yes
173	M435T36	435.36	35.59	31		yes
193	M285T36	285.29	35.76	32		low quality
279	M404T36	404.25	35.78	32		yes
213	M342T34	342.25	33.51	33		yes
138	M463T38	463.39	38.41	34		yes
144	M448T30	448.26	29.83	36	**(15)**	yes
290	M440T30	440.30	29.83	36		yes
51	M348T41	348.26	41.48	38		yes
275	M365T44	365.28	43.90	41		yes
123	M288T34	288.30	34.08	42		yes
283	M367T46	367.29	45.56	43		yes
72	M383T38	383.27	37.52	44		yes
81	M443T37	443.29	37.46	44		yes
200	M504T44	504.34	44.33	45		yes
7	M450T33	450.29	32.55	48		yes
112	M447T36	447.36	35.70	49		yes
137	M460T37	460.31	37.21	52	**(14)**	yes
85	M461T38	461.30	38.02	53		yes

peak N°	name	mzmed	rtmed	peak group	assignement	MSMS
37	M488T30	488.27	30.44	54		low quality
12	M447T31	447.25	31.29	55		low quality
210	M309T38	309.20	38.15	63		low quality
30	M465T26	465.26	26.12	71		low quality
207	M301T3	301.15	3.21	76		yes
24	M464T31	464.27	31.35	78		low quality

33

Table S4. ^{1}H (600 MHz) and ^{13}C (150 MHz) NMR data of demethylfurospongin-4 (**1**) recorded in $CDCl_3$.

	δ_C	δ_H (*J* in Hz)
1	142.5	7,33 (dd, 1.6; 2.0)
2	111.1	6.27 (brs)
3	125.0	_
4	138.8	7.20 (brs)
5	25.0	2.45 (brt, 7.5)
6	28.4	2.24 (td, 7.5; 8)
7	123.8	5.17 (tq, 7.2; 1.1)
8	135.7	_
9	16.1	1.59 (brs)
10	39.6	1.99 (brt, 7.3)
11	26.4	2.08 (m)
12	125.0	5.12 (tq, 6.9; 1.1)
13	134.0	_
14	15.8	1.59 (brs)
15	38.9	2.08 (m)
16	28.0	2.53 (m)
17	144.6	6.01 (brt, 7.3)
18	131.2	_
19	174.0	_
20	33.1	2.53 (m)
21	30.7	2.36 (dt, 7.5; 8)
22	143.7	6.94 (brt, 8.2)
23	128.0	_
24	11.7	1.79 (brs)
25	173.3	_

Table S5. ^{1}H (600 MHz) and ^{13}C (150 MHz) NMR data of furospongin-1 (**11**) recorded in CD_3OD.

	δ_C	δ_H (J in Hz)
1	143.8*	7.36¤ (t, 1.7)
2	112.0**	6.31 (m)
3	126.1	_
4	140.2¤	7.25 (m)
5	25.8¤¤	2.46 (bt, 7.4)
6	29.5	2.27 (tdd, 7.5; 8.0; 3.5)
7	127.8	5.21 (tq, 7.0; 1.2)
8	134.0	_
9	16.4	1.6 (bs)
10a	49.9	2.14 (dd, 15.0; 6.3)
10b		2.04 (dd, 15.0; 6.3)
11	68.2	3.76 (m)
12a	45.3	1.11 (ddd, 10.0; 3.2; 3.2)
12b		1.34 (ddd, 10.0; 3.9; 3.9)
13	30.1	1.7 (m)
14	19.6	0.88 (d, 7.0)
15a	38.6	1.31 (m)
15b		1.21 (m)
16	28.6	1.58 (m)
17	25.8¤¤	2.40 (t, 7.5)
18	126.5	_
19	140.0¤	7.26
20	111.9**	6.29 (m)
21	143.8*	7.37¤ (t, 1.7)

* overlapping signals, attributions can be exchanged
** overlapping signals, attributions can be exchanged
¤ overlapping signals, attributions can be exchanged
¤¤ overlapping signals, attributions can be exchanged

Table S6. Assignment of selected nodes from the furanoterpene cluster observed in the molecular network

Node	Measured *m/z*	Calculated *m/z*	Mass accuracy Δm (ppm)	Molecular formula	Matching compound or partial structural information	Ref.
341	341.1745	341.1747	-0.59	$C_{21}H_{24}O_4$	Nitenin	[1,2]
343	343.2238	343.2268	-8.74	$C_{22}H_{30}O_3$		[3]
346	346.2381	346.2377	1.16	$C_{21}H_{31}NO_3$	Pyrrolo-furanoterpene	
347	347.2197	347.2217	-5.76	$C_{21}H_{30}O_4$	7,8-epoxyfurospongin-1 type	[4]
358	358.2383	358.2377	1.67	$C_{22}H_{31}NO_3$	Pyrrolo-furanoterpene	
359	359.2215	359.2217	-0.56	$C_{22}H_{30}O_4$		[5]
399	399.2527	399.2530	-0.75	$C_{25}H_{34}O_4$	Tetronic acid derivative	[6]
401	401.2677	401.2686	-2.24	$C_{25}H_{36}O_4$	Epoxy derivative	[7]
402	402.2286	402.2275	2.73	$C_{23}H_{31}NO_5$	Glycinyl lactam derivative	
418	418.2580	418.2588	-1.91	$C_{24}H_{35}NO_5$	Glycinyl lactam derivative	
429	429.2646	429.2636	2.33	$C_{26}H_{36}O_5$	Isofurospongin-4 type	[4]
446	446.2892	446.2901	-2.02	$C_{26}H_{39}NO_5$	Glycinyl lactam derivative	
494	494.2865	494.2901	-7.28	$C_{30}H_{39}NO_5$	Glycinyl lactam derivative	

Table S7. Product ions from spongialactam A **(12a)** obtained with LC-MS/MS (+) ESI-Q-ToF at 20 eV.

m/z_{Exp}	Relative abundance %	Fragment Formula	m/z_{Calc}	Δ_{ppm}
135.0805	100	$C_9H_{11}O$	135.0804	0.74
163.1113	14.4	$C_{11}H_{15}O$	163.1117	-2.45
182.0813	16.7	$C_9H_{12}NO_3$	182.0812	0.55
208.1330	23.8	$C_{12}H_{18}NO_2$	208.1332	0.96
236.1276	10.1	$C_{13}H_{18}NO_3$	236.1281	-2.12
254.1386	28.4	$C_{13}H_{20}NO_4$	254.1387	-0.39

Table S8. Product ions from spongialactam A **(12a)** obtained with LC-MS/MS (-) ESI-Q-ToF at 20 eV.

m/z Exp	Relative abundance %	Fragment Formula	*m/z* Calc	Δppm
149.0969	15.9	$C_{10}H_{13}O$	149.0972	-2.01
208.1336	54.3	$C_{12}H_{18}NO_2$	208.1343	-3.36
252.1234	0.9	$C_{13}H_{18}NO_4$	252.1241	-2.78
358.2370	1.4	$C_{22}H_{32}NO_3$	358.2388	-5.02
402.2280	100	$C_{23}H_{32}NO_5$	402.2286	-1.49

Table S9. Product ions from spongialactam B **(12b)** obtained with LC-MS/MS (+)ESI-Q-ToF at 20 eV.

m/z Exp	Relative abundance %	Fragment Formula	*m/z* Calc	Δppm
135.0810	27.8	$C_9H_{11}O$	135.0804	4.44
149.0954	17.4	$C_{10}H_{13}O$	149.0961	-4.70
154.0495	14.9	$C_7H_8NO_3$	154.0499	-2.60
163.1112	39.9	$C_{11}H_{15}O$	163.1117	-3.07
178.1221	50.5	$C_{11}H_{16}NO$	178.1226	-2.81
224.1281	100.0	$C_{12}H_{18}NO_3$	224.1281	0.02

Table S10. Product ions from spongialactam B **(12b)** obtained with LC-MS/MS (-)ESI-Q-ToF at 20 eV.

m/z Exp	Relative abundance %	Fragment Formula	*m/z* Calc	Δppm
110.0617	32.4	C_6H_8NO	110.0611	5.45
151.1127	0.4	$C_{10}H_{15}O$	151.1128	-0.66
178.1242	68.7	$C_{11}H_{16}NO$	178.1237	2.81
222.1144	0.5	$C_{12}H_{16}NO_3$	222.1136	3.60
358.2388	1.9	$C_{22}H_{32}NO_3$	358.2388	0.00
402.2288	100	$C_{23}H_{32}NO_5$	402.2286	0.50

Figure S1. Overlay of the total ion chromatograms of the extracts of *S. officinalis* generated by LC-MS in positive ion mode (after retention time alignment with XCMS Online).

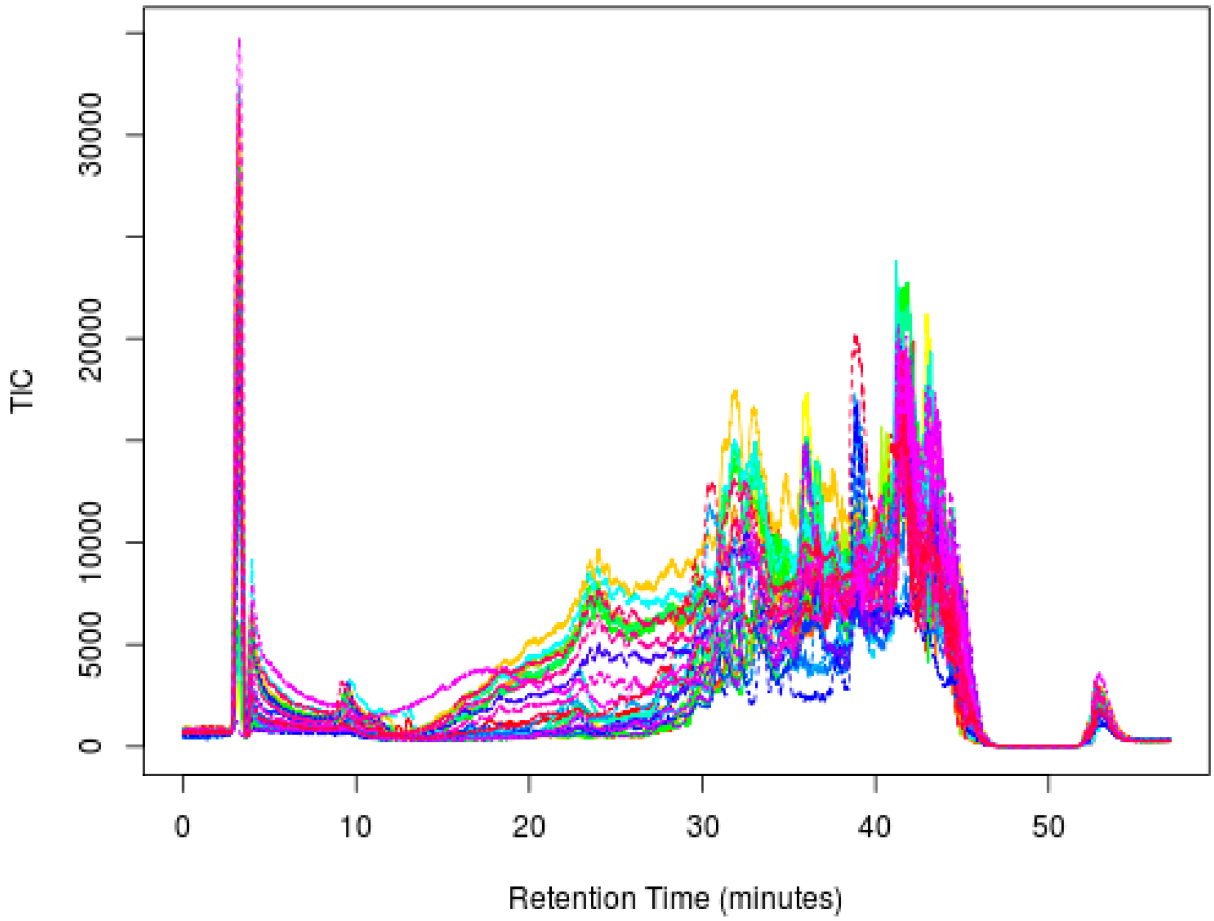

Figure S2. PCA analysis of not-normalized (a-c) versus normalized (d-e) dataset.

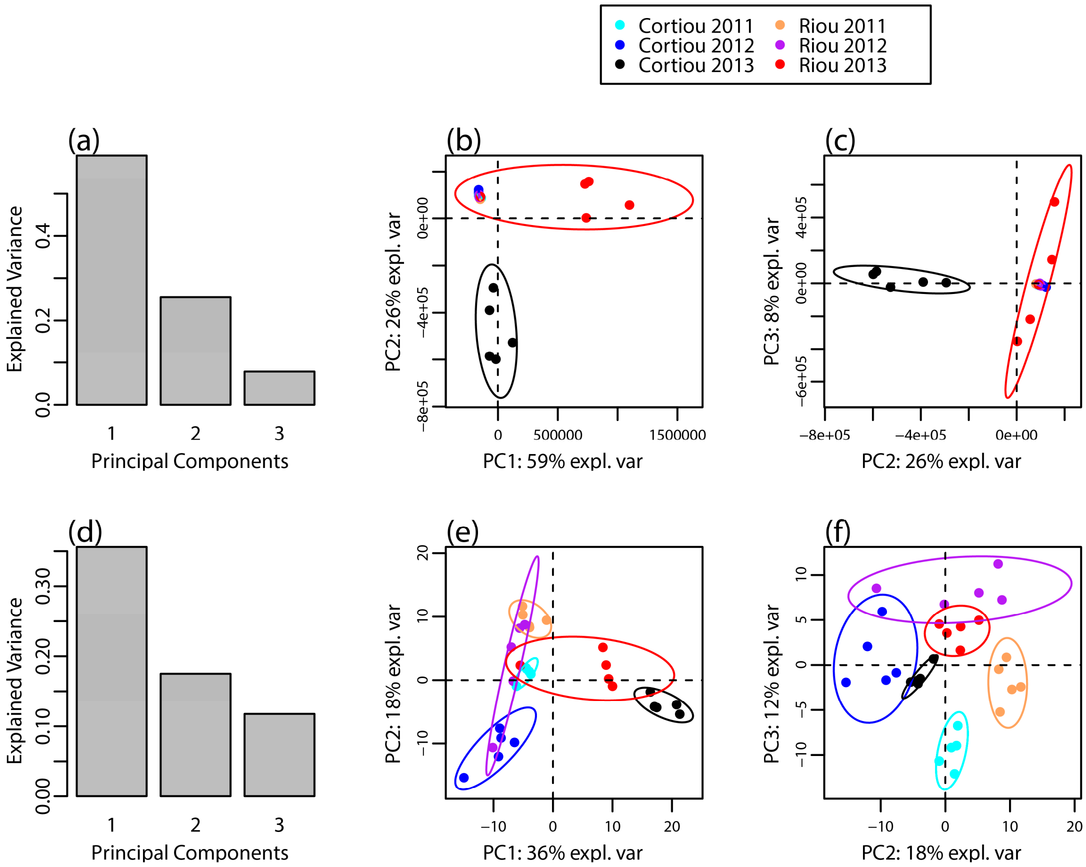

Figure S3. VIP scores on the first three components of the PLS-DA (for the most contributing variables, *i.e.*, values > 0.95 × max (VIP score)). The variables are labeled MxTy, where x denotes the nominal *m*/*z* ratio and y indicates the nominal retention time.

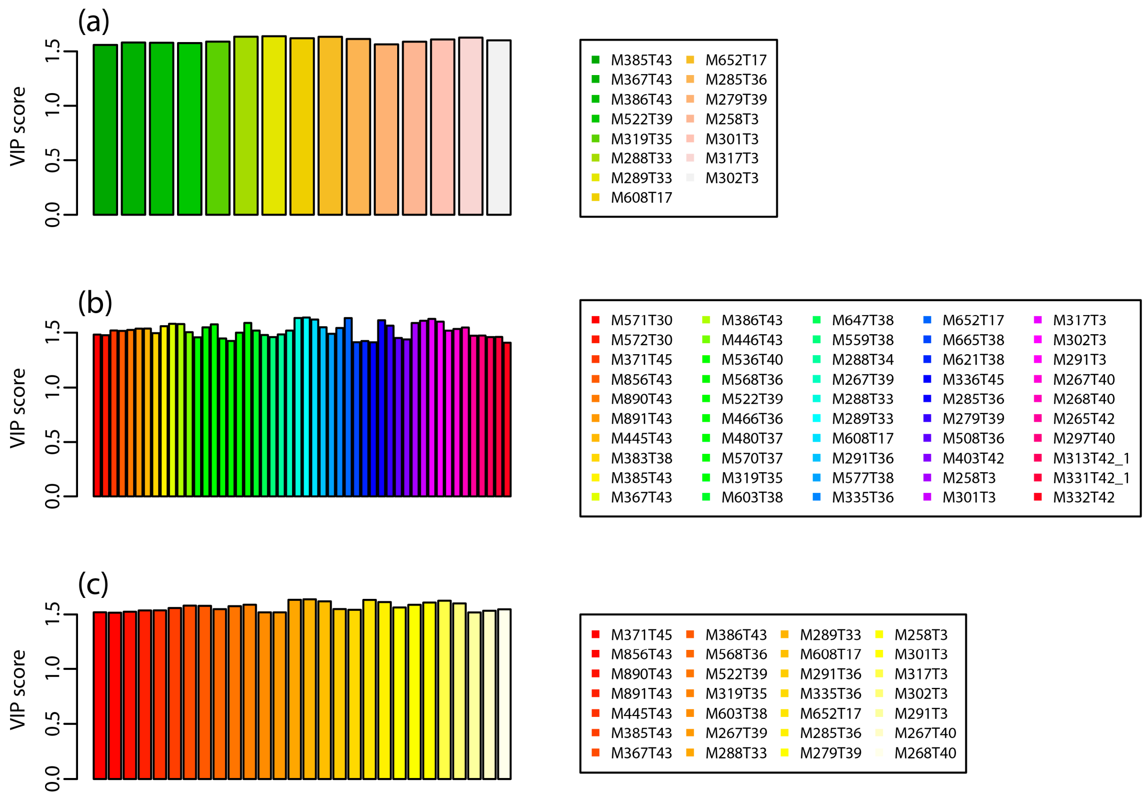

Figure S4. Score (A-B) and loading (C-D) plots of sPLS-DA with 3 components and 10, 30 and 30 variables selected on each component, selectively. The variables are labeled MxTy, where x denotes the nominal *m*/*z* ratio and y indicates the nominal retention time.

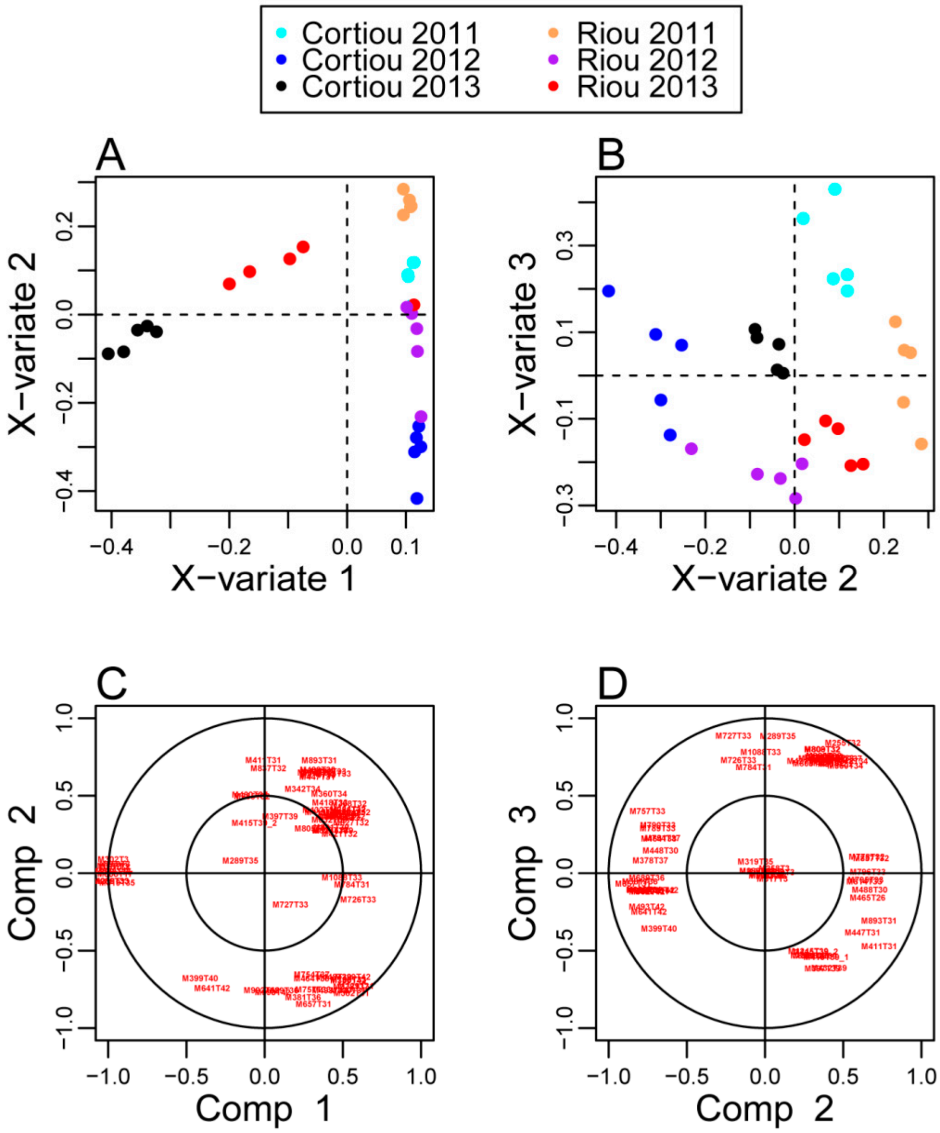

Figure S5. ^{1}H NMR spectrum of demethylfurospongin-4 (**1**) in $CDCl_3$ (600 MHz).

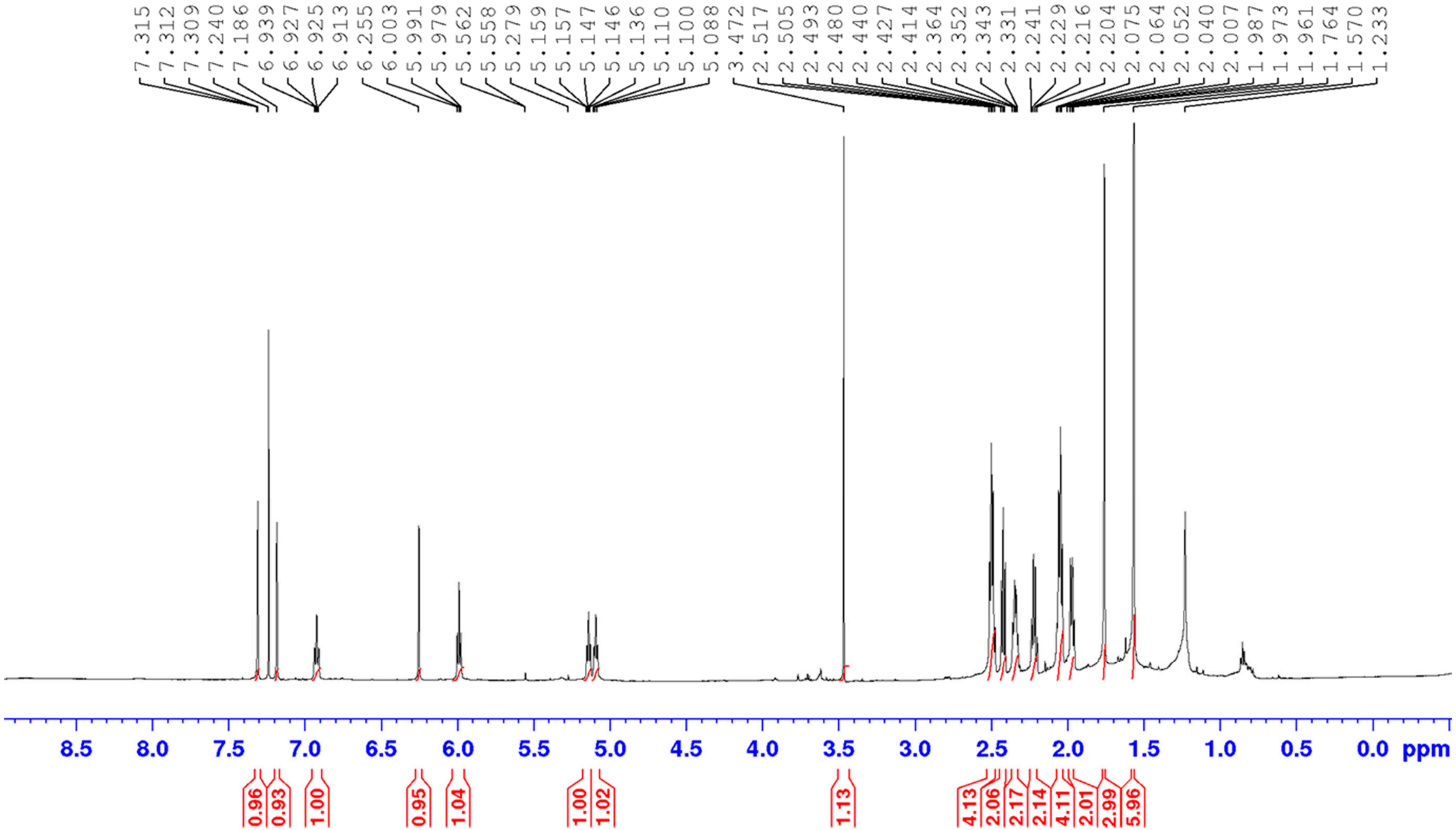

Figure S6. ^{13}C NMR spectrum of demethylfurospongin-4 (**1**) in $CDCl_3$ (150 MHz).

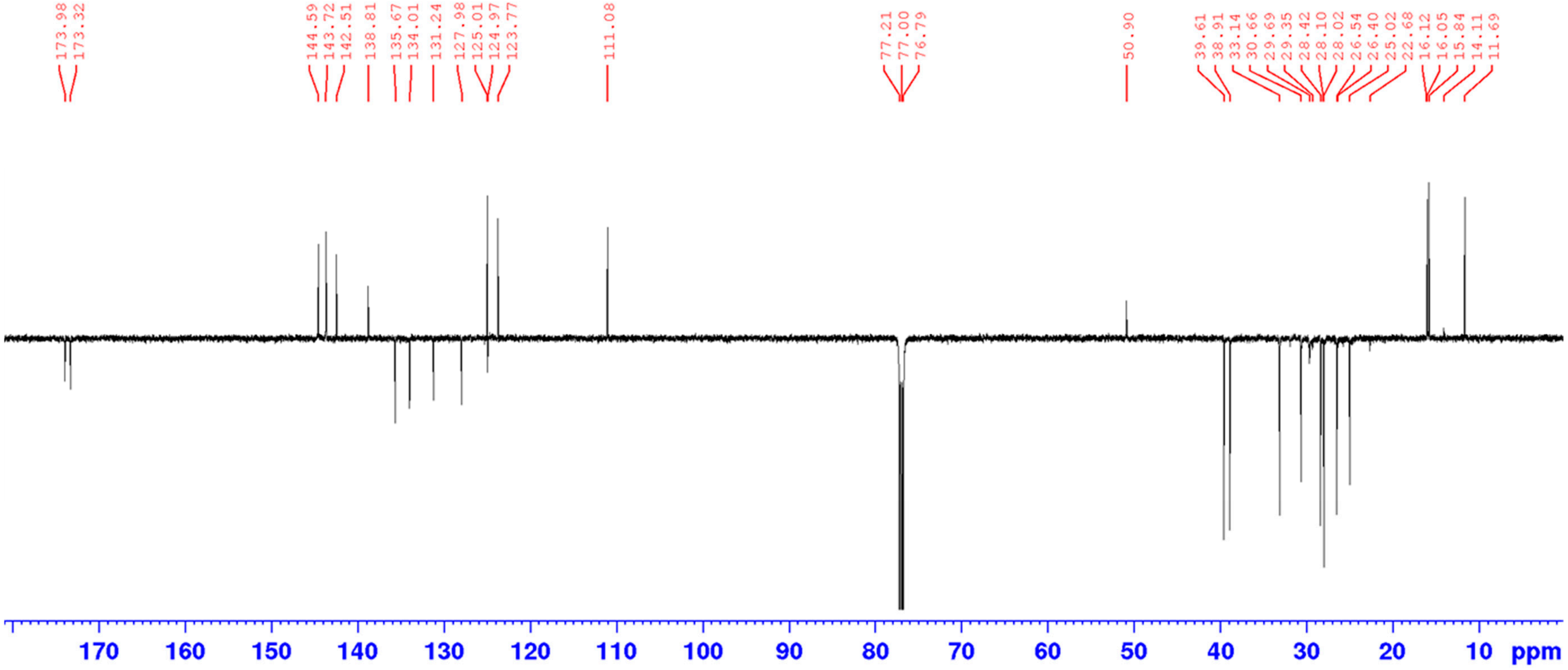

Figure S7. LC-MS/MS spectra of the $[M+H]^+$ species of compounds **3** (A, *m/z* 445.3), **4** (B, *m/z* 568.4) and **5**, assigned to coconut diethanolamide (C_{11} DEA) [8] (C, *m/z* 288.3), (CE 20 eV).

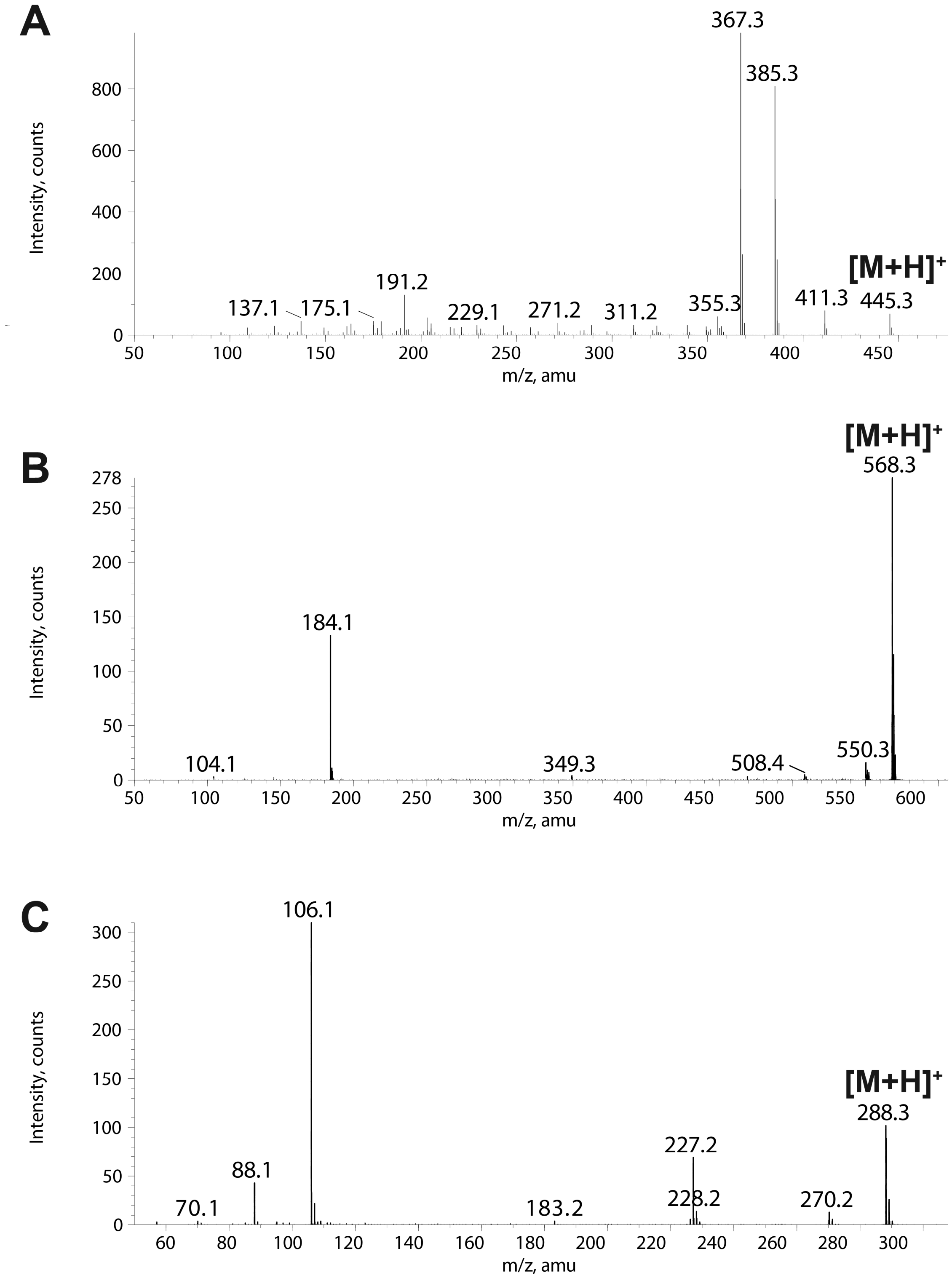

Figure S8. LC-MS/MS spectra of the [M+H]⁺ species of compounds **6** (A, *m/z* 360.3), **13** (B, *m/z* 377.2), **14** (C, *m/z* 460.3) and **15** (D, *m/z* 448.3), (CE 20 eV). Assignment for compound **6** is based on similarities with spongialactam A (**12a**), see Figure 4. *E:* Known furanoterpenes sharing the same molecular formula than compound **13**.

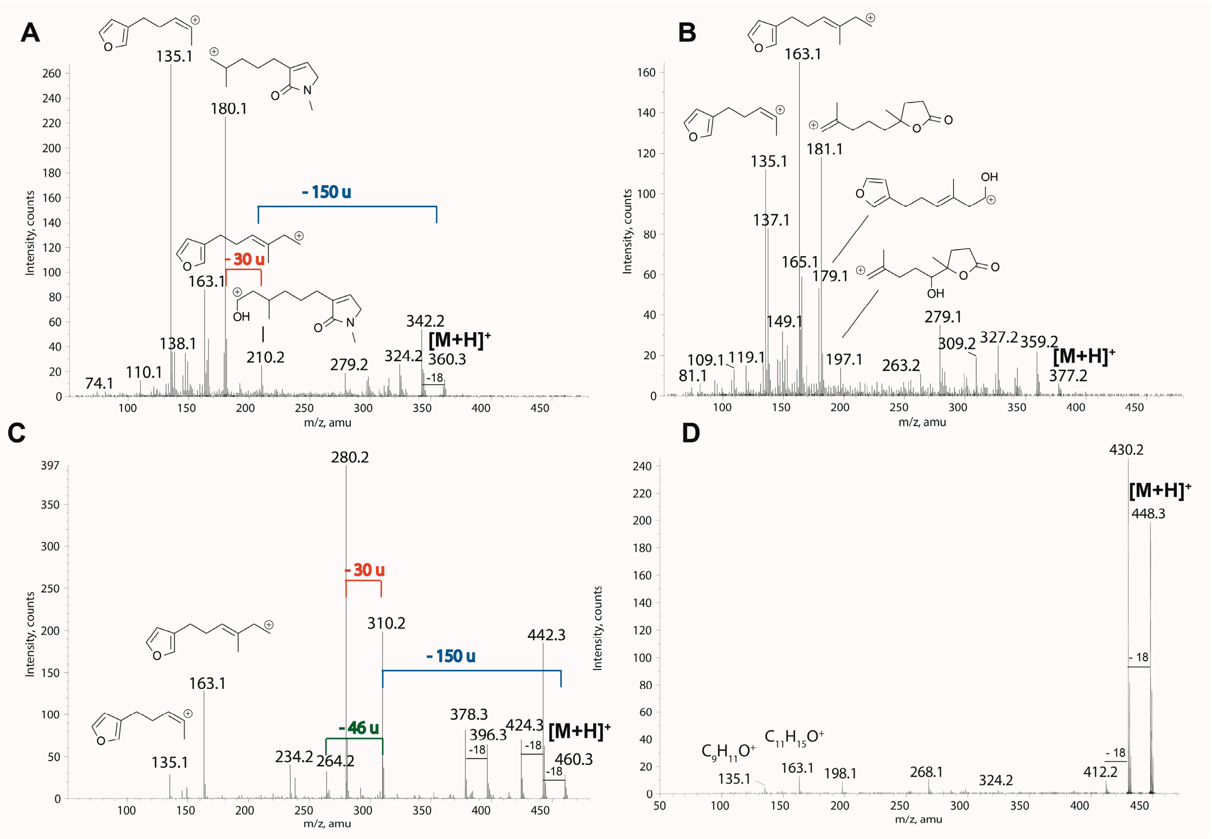

E

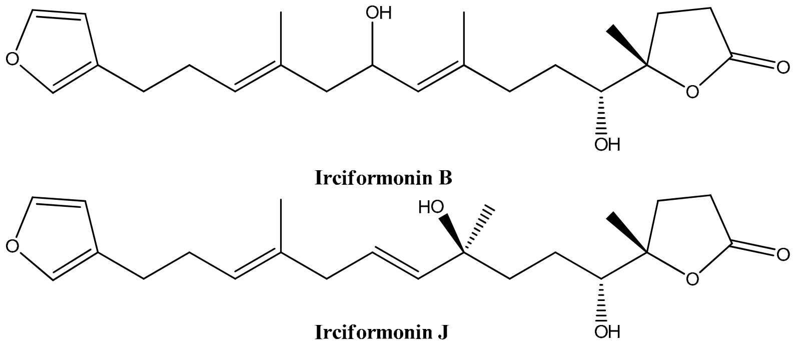

Irciformonin B

Irciformonin J

Figure S9. LC-MS/MS spectra of the $[M+H]^+$ species of **A**) compound **7** and **B**) compound **8** (*m*/*z* 329.21), (CE 20 eV). The main product ions are represented. **C**) Known furanoterpenes sharing the same molecular formula and compatible with the MS/MS data of compound **7**: furospongenone [9] and dihydrofurospongin-2 [10].

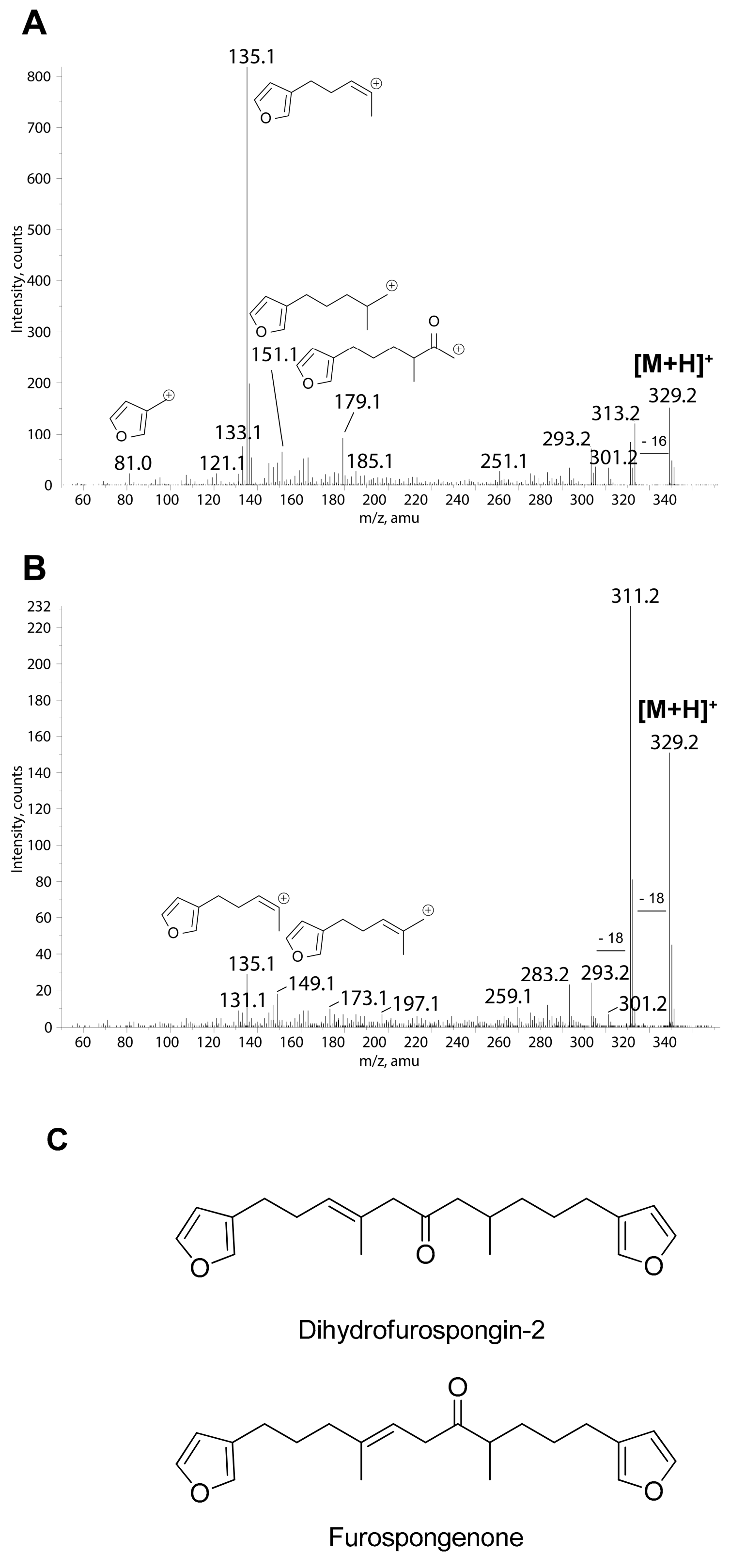

Figure S10. LC-MS/MS spectra of compounds **9** and **10** in positive (**A**, **C**) and negative (**B**, **D**) ion modes. **A**) [M+H]$^+$ species of compound **9** (*m/z* 363.22), (CE 20 eV). **B**) [M-H]$^-$ species of compound **9** (*m/z* 361.20), (CE 30 eV). **C**) [M+H]$^+$ species of compound **10** (*m/z* 363.22), (CE 20 eV). **D**) [M-H]$^-$ species of compound **10** (*m/z* 361.20), (CE 30 eV). The main product ions are represented. **E**) Known furanoterpenes sharing the same molecular formula and compatible with the MS/MS data of compound **9**: isomers of γ-hydroxy-α,β-butenolide and β,γ-epoxybutenolidesfurospongin-1 [11].

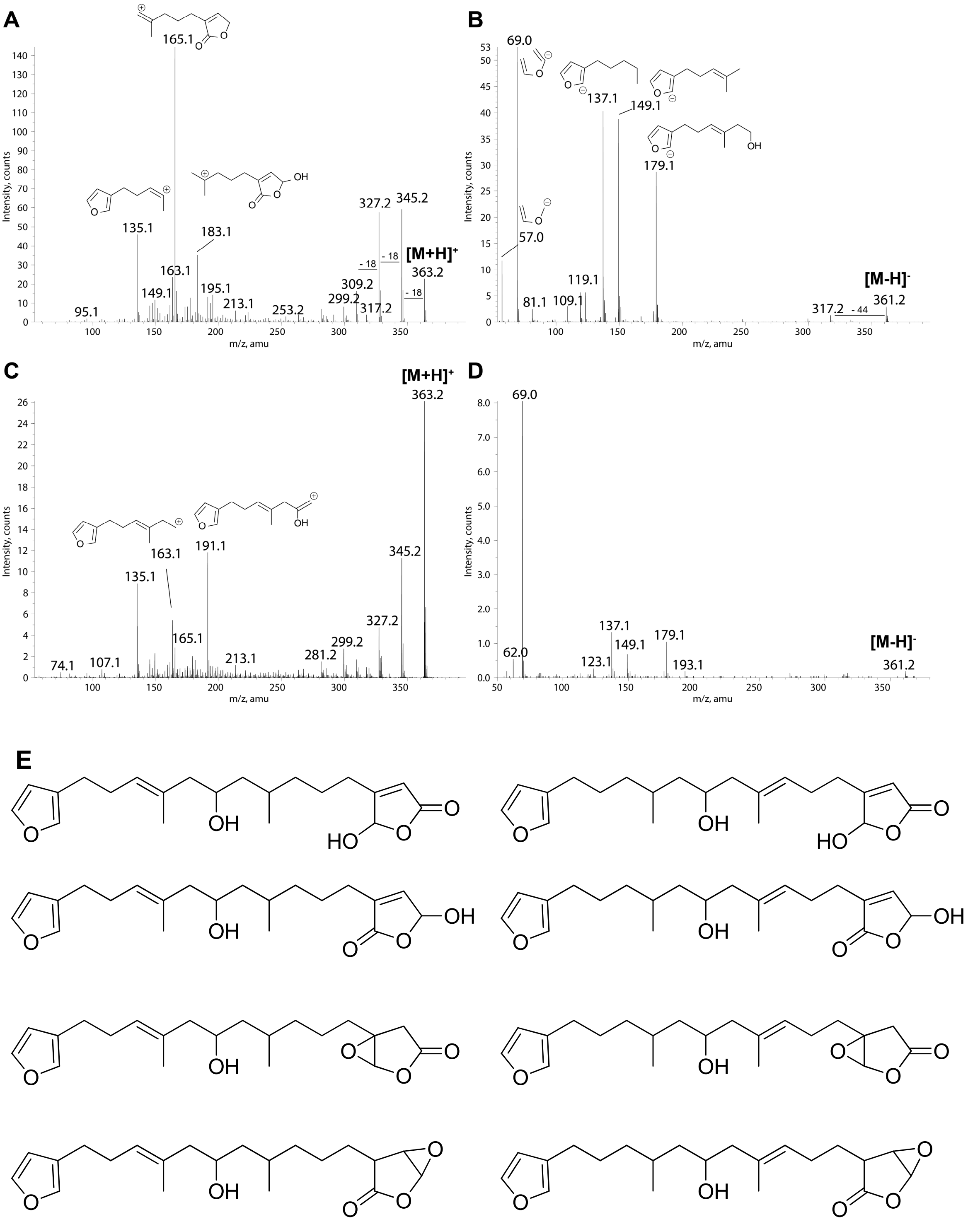

Figure S11. **A**) LC-MS/MS spectrum of the [M+H]$^+$ species of compound **11** (*m/z* 331.23), (CE 20 eV). The main product ions are represented. **B**) Known furanoterpenes sharing the same molecular formula and compatible with the MS/MS data: furospongin-1 [12], tetrahydrofurospongin-2 [10] and furospongenol [9].

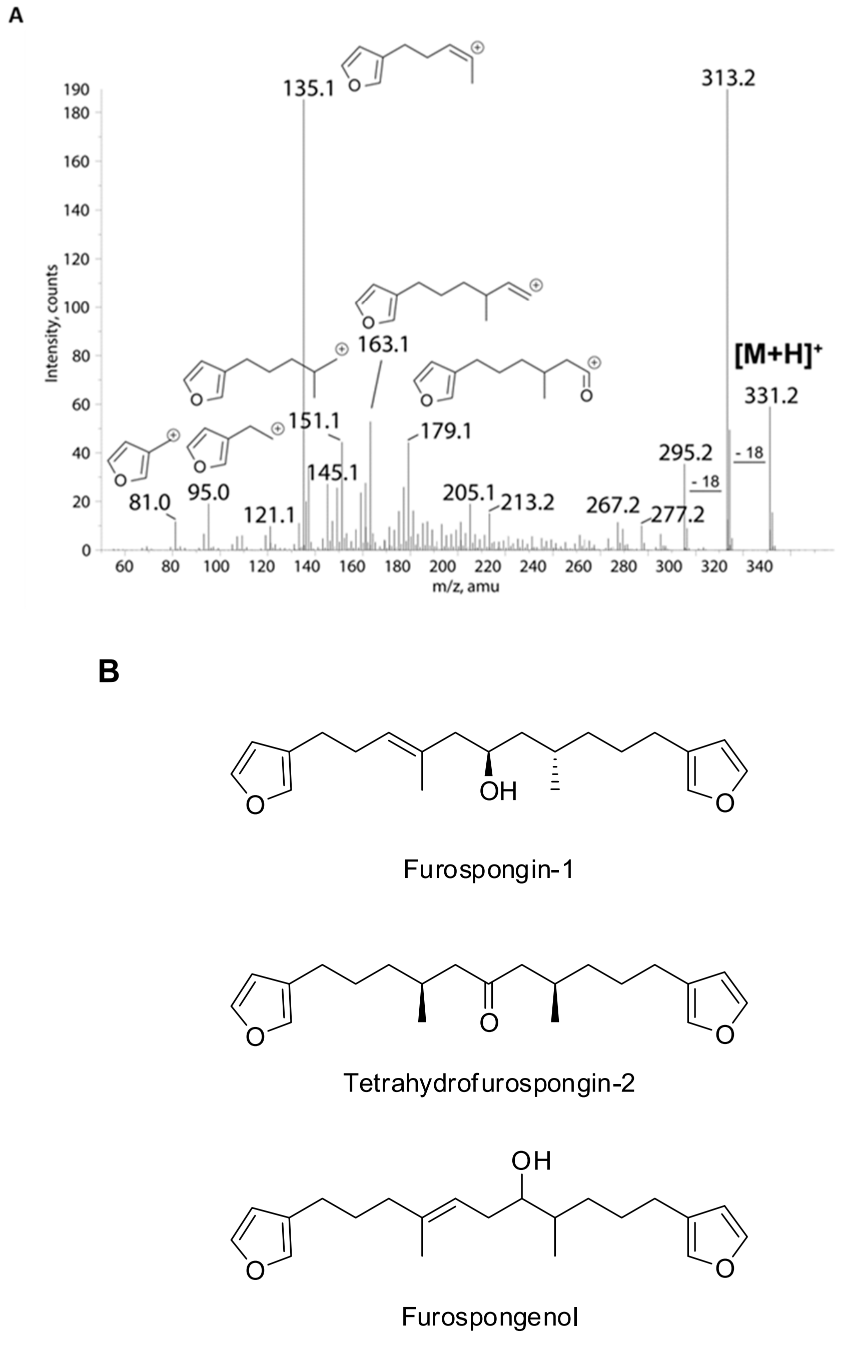

124 **Figure S12**. ^{1}H NMR spectrum of furospongin-1 (**11**) CD_3OD (600 MHz).

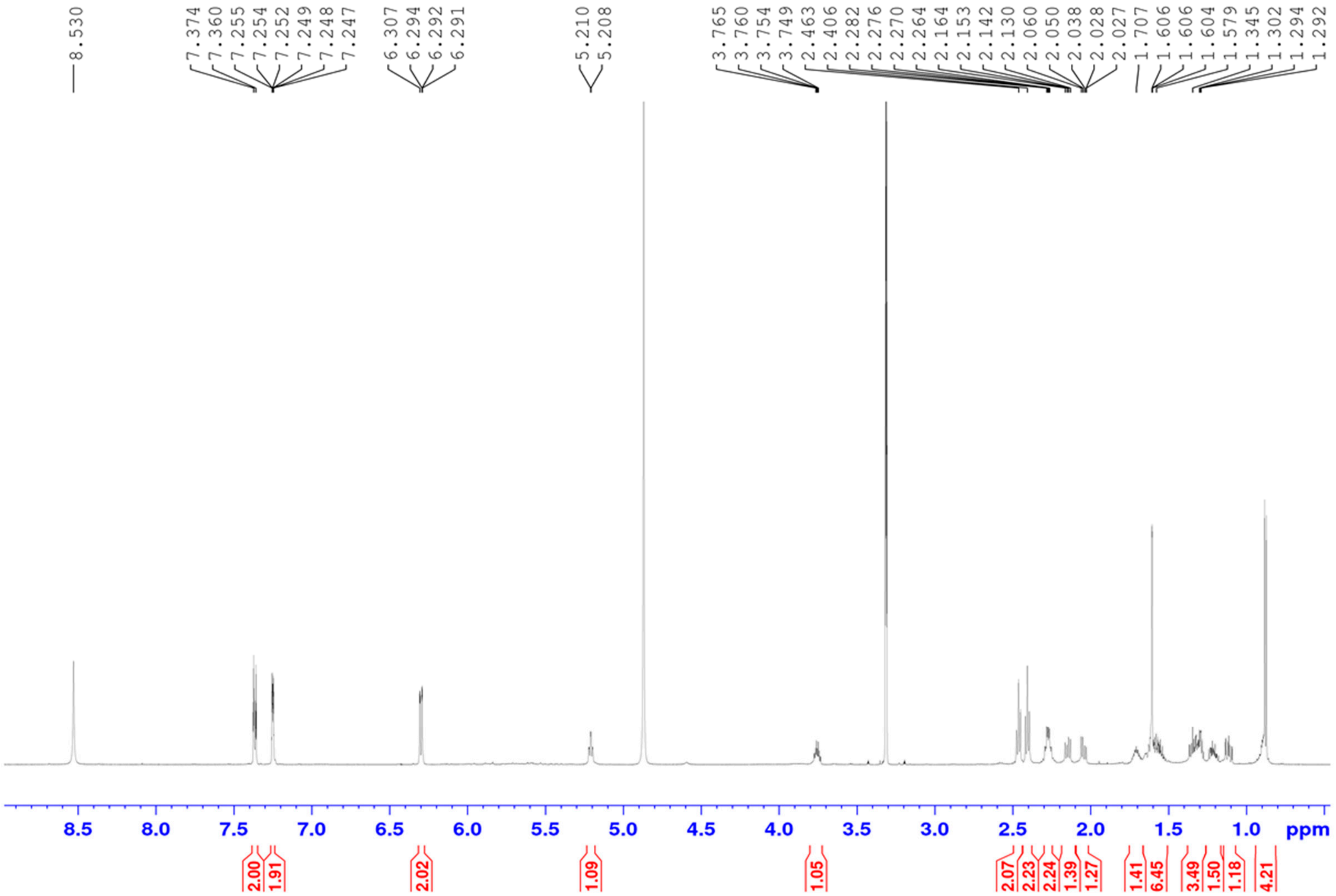

125

Figure S13. DEPT NMR spectrum of furospongin-1 (**11)** in CD_3OD (150 MHz).

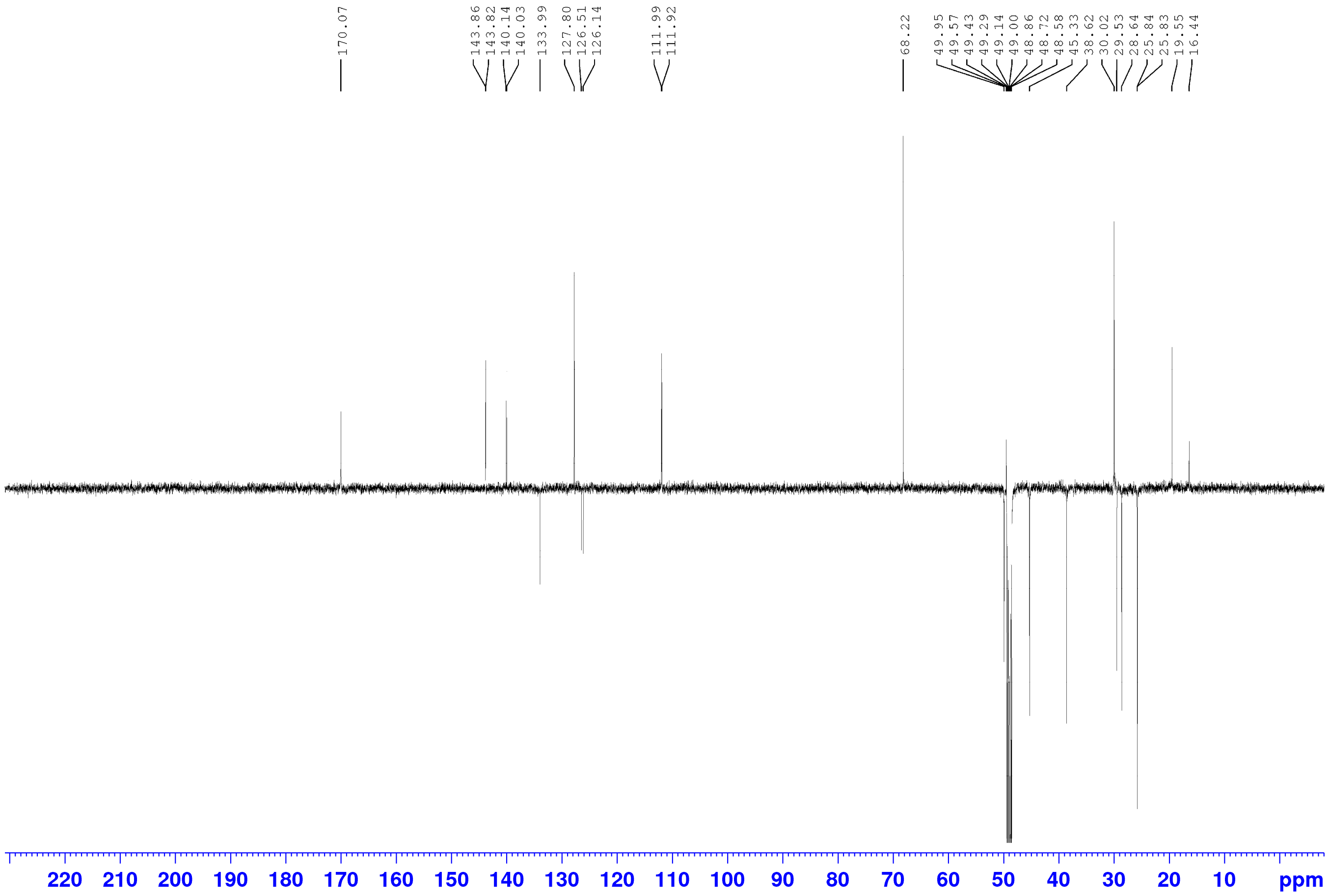

Figure S14. ^{1}H NMR spectrum of furofficin (**2**) in CD_3OD (600 MHz).

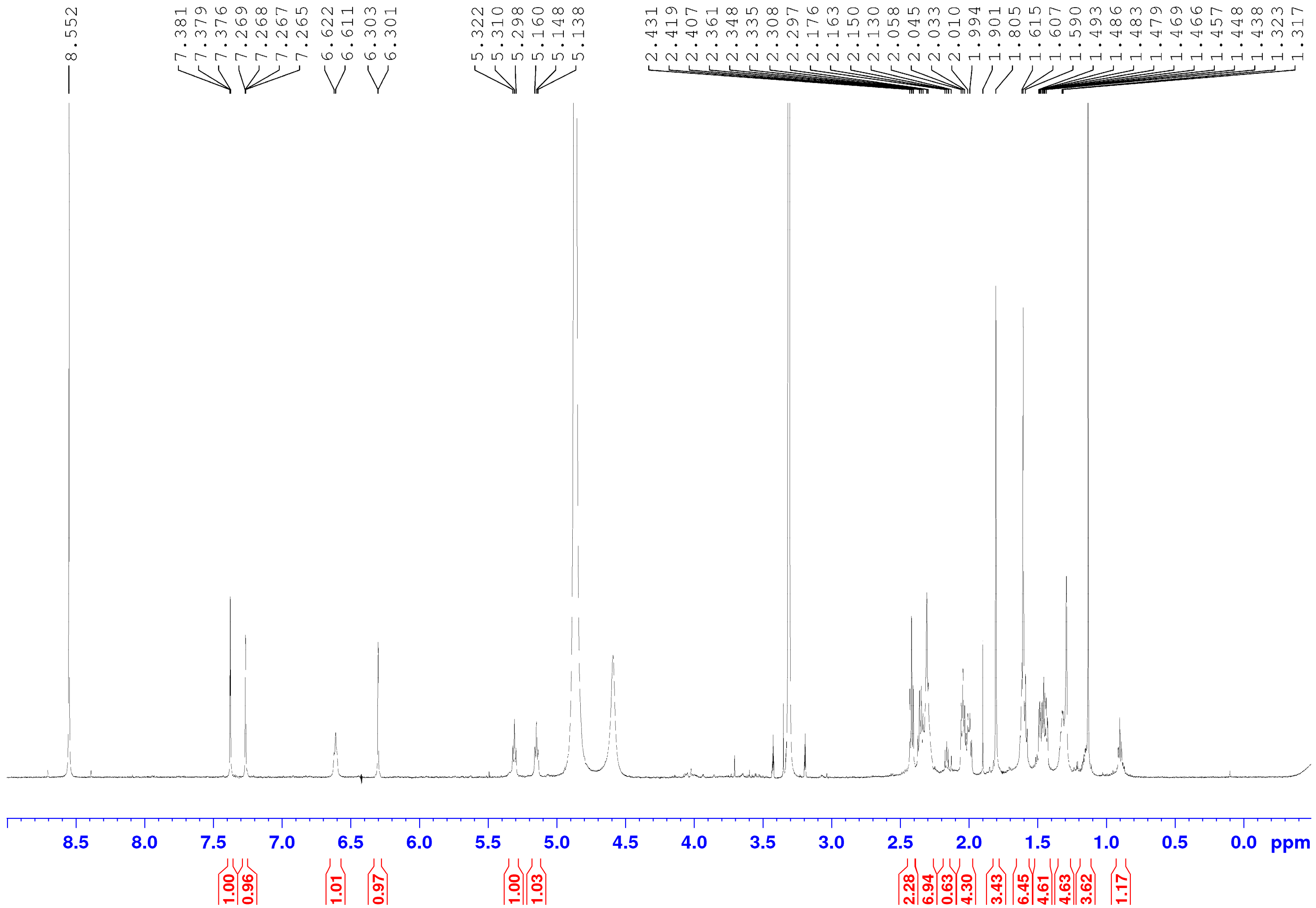

Figure S15. ^{1}H-^{1}H COSY spectrum of furofficin (**2**) in CD_3OD (600 MHz).

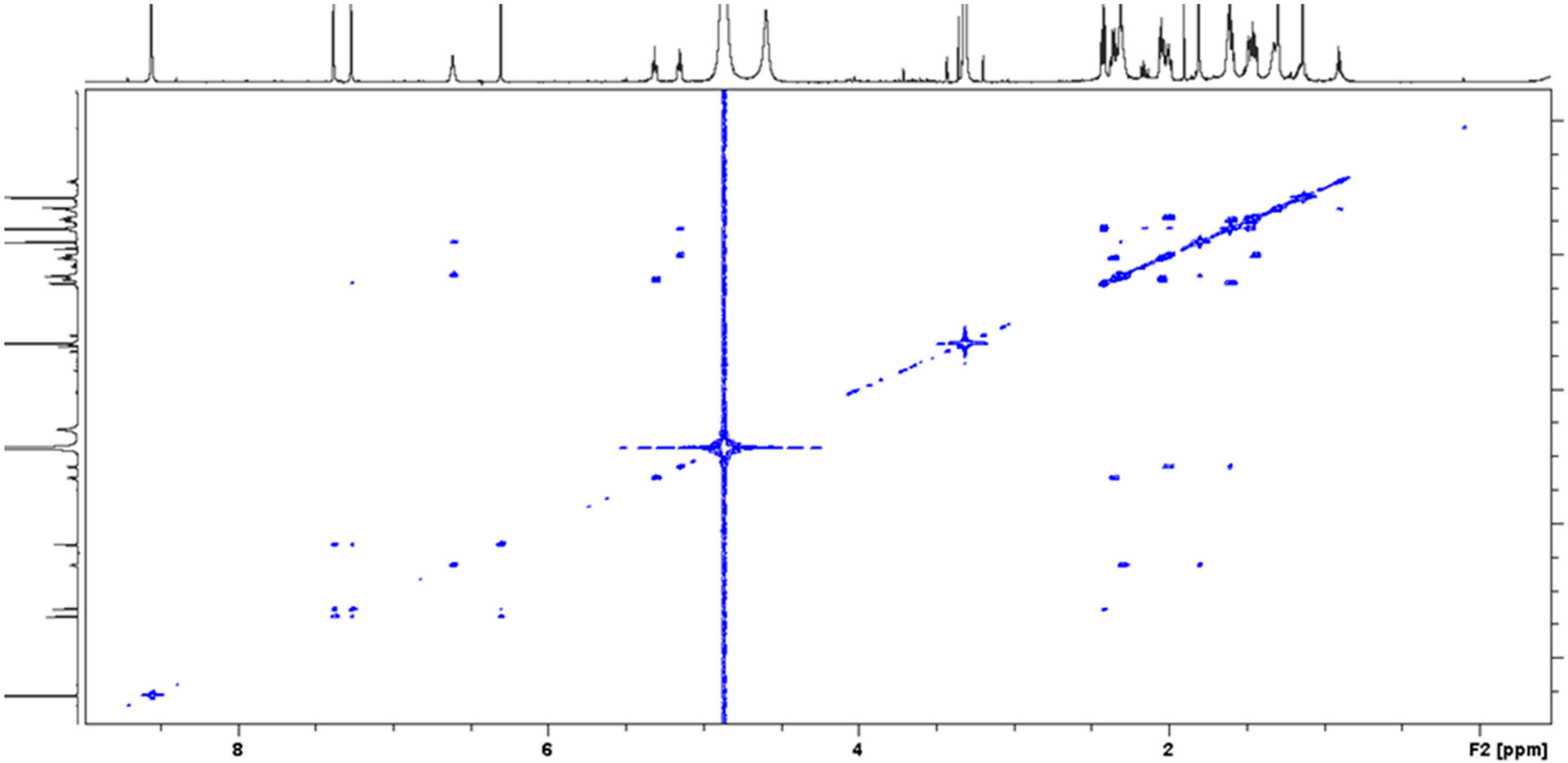

Figure S16. HSQC spectrum of furofficin (**2**) in CD_3OD (600 MHz).

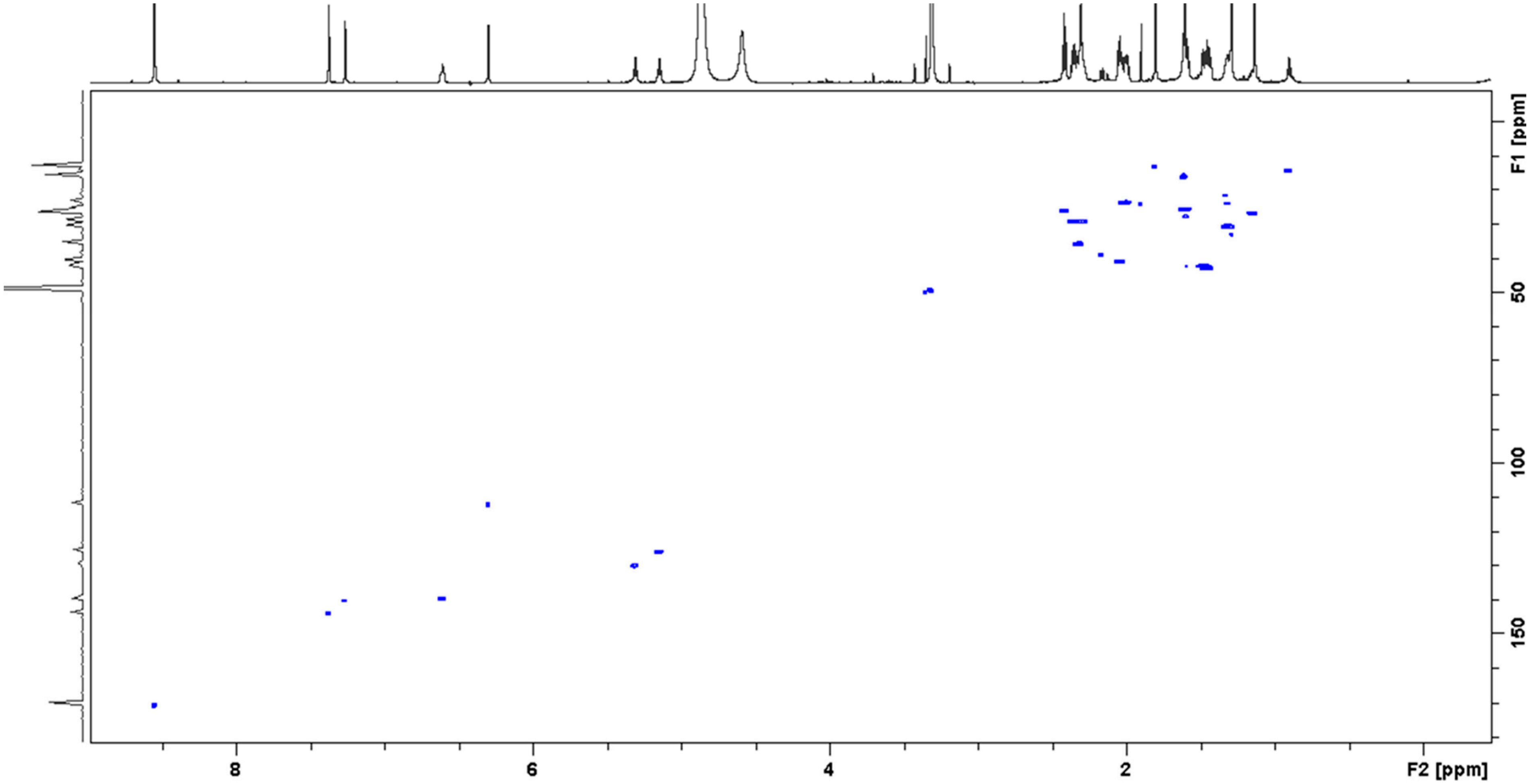

Figure S17. HMBC spectrum of furofficin (**2**) in CD_3OD (600 MHz).

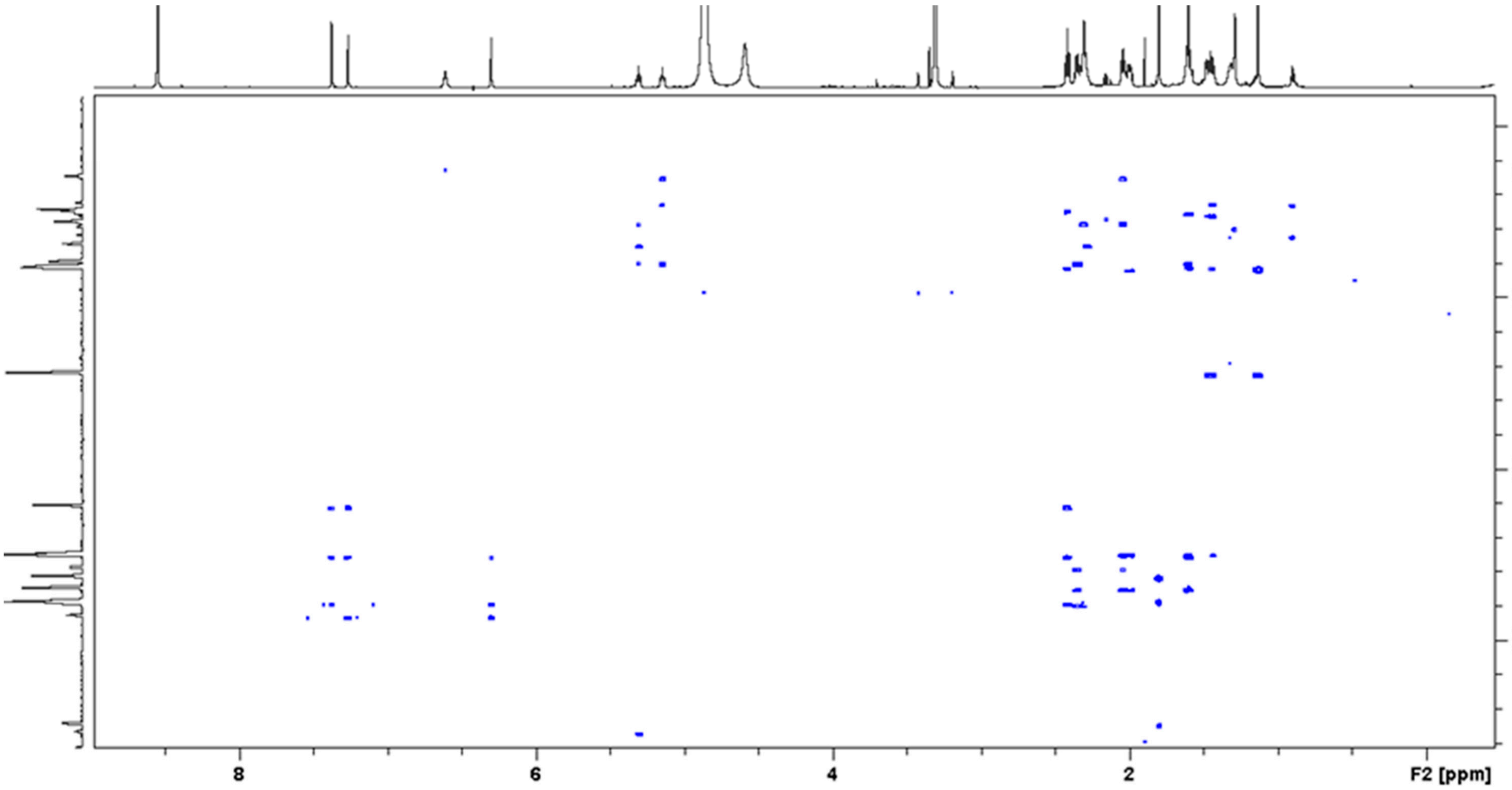

Figure S18. NOESY spectrum of furofficin (**2**) in CD_3OD (600 MHz).

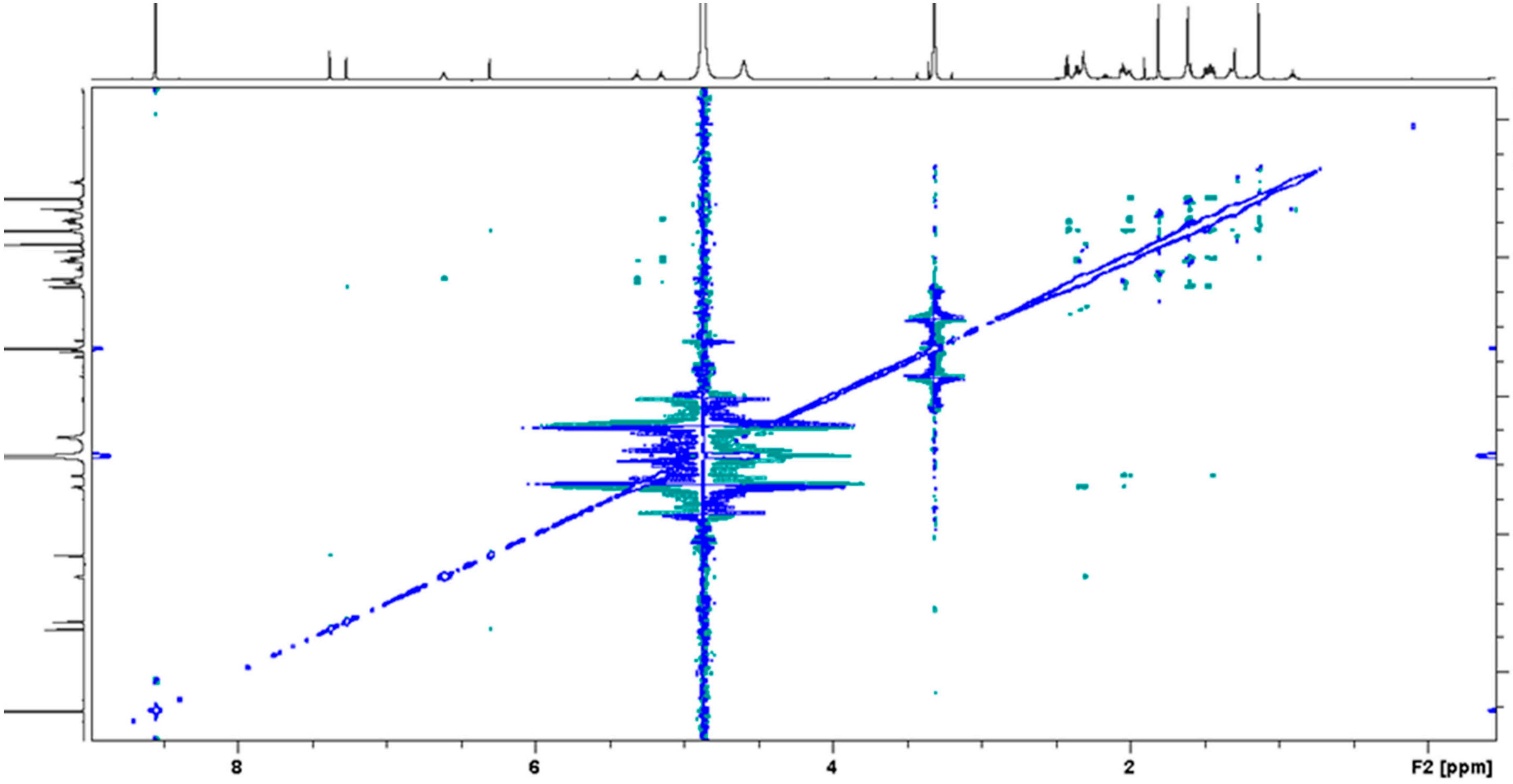

Figure S19. ^{1}H NMR spectrum of pyrrolospongicin-1 (**12a**) in CD_3OD (600 MHz)

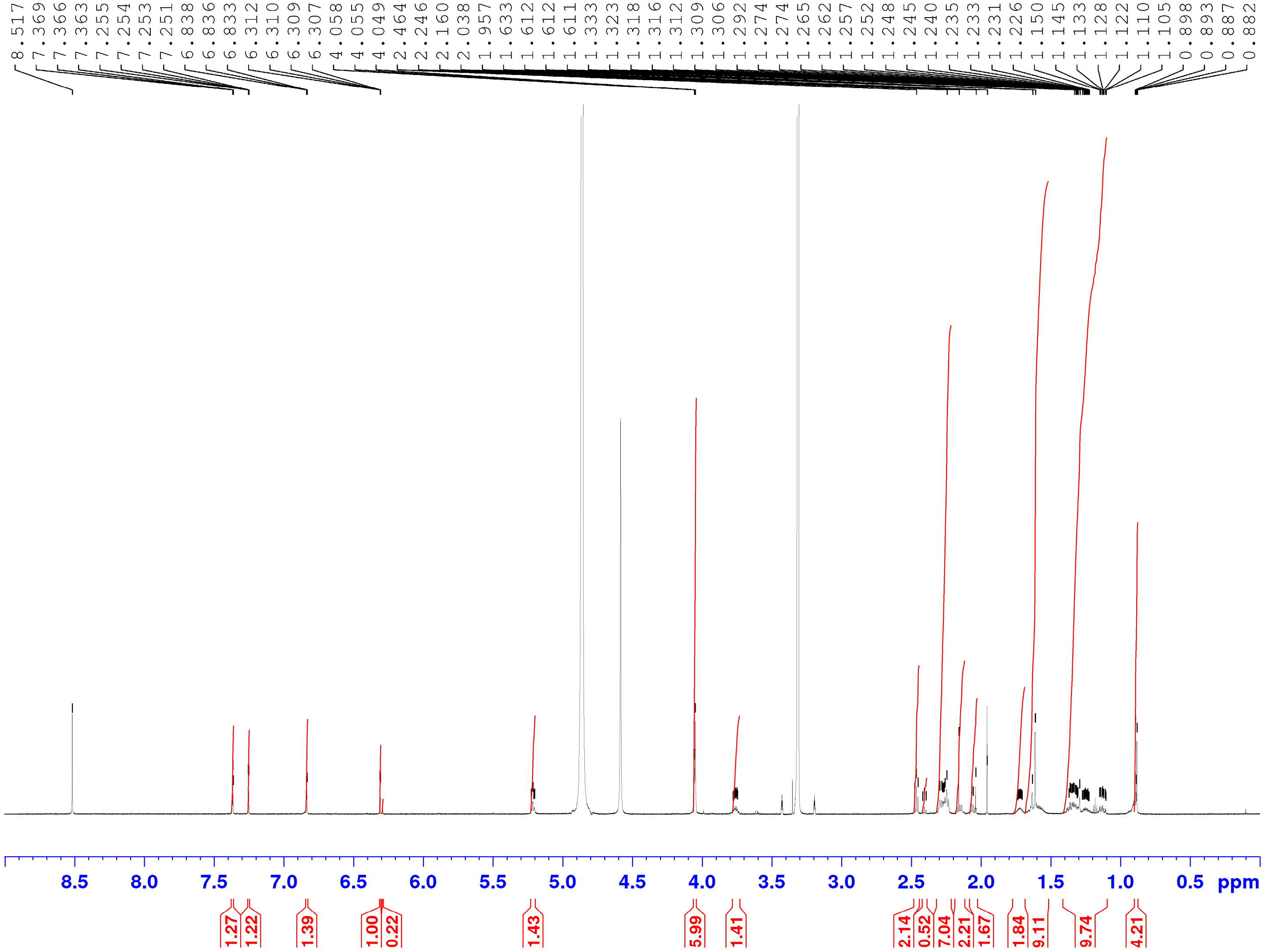

Figure S20. ^1H-^1H COSY spectrum of pyrrolospongicin-1 (**12a**) in CD_3OD (600 MHz).

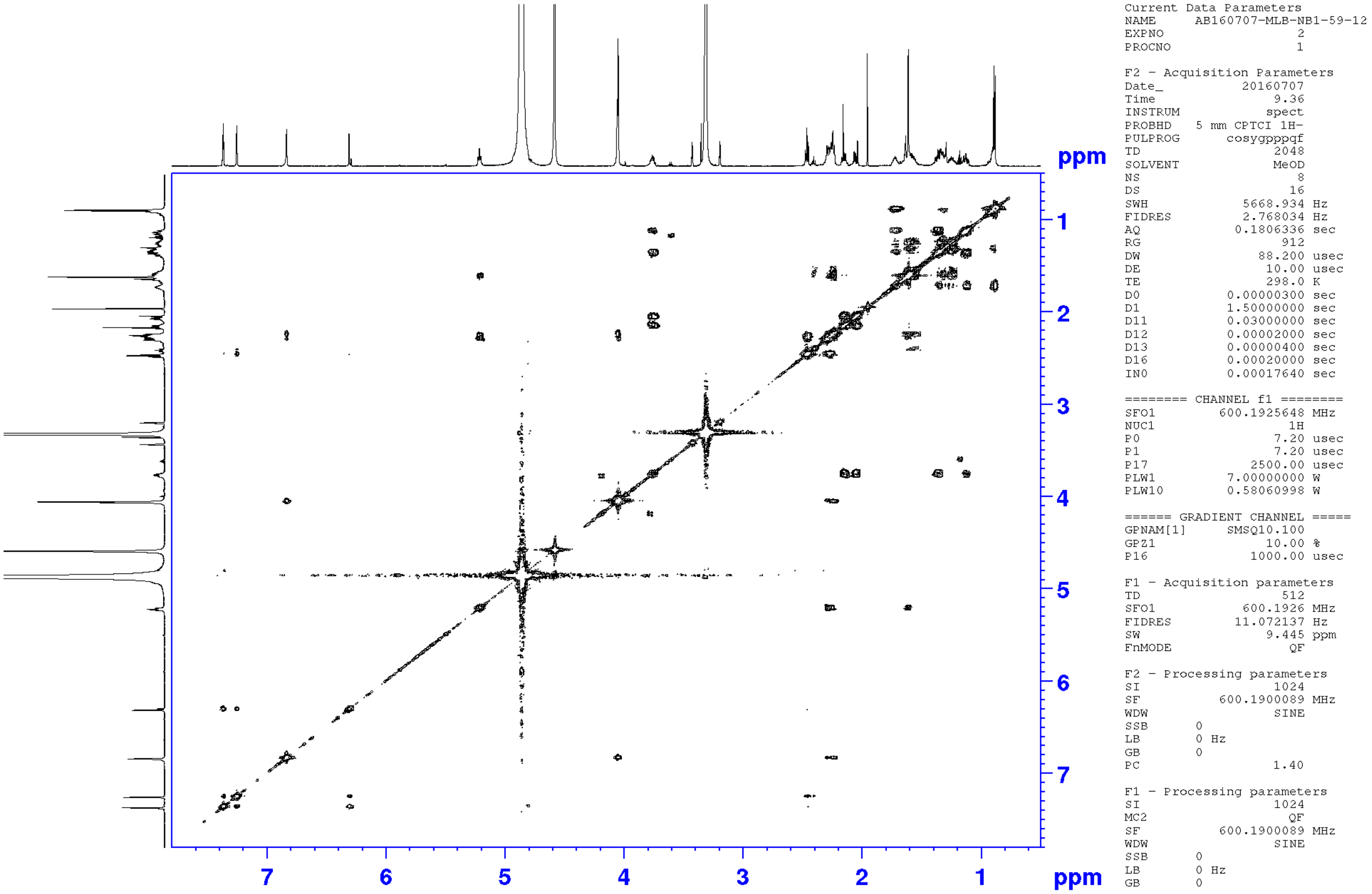

Figure S21. HSQC spectrum of pyrrolospongicin-1 (**12a**) in CD_3OD (600 MHz).

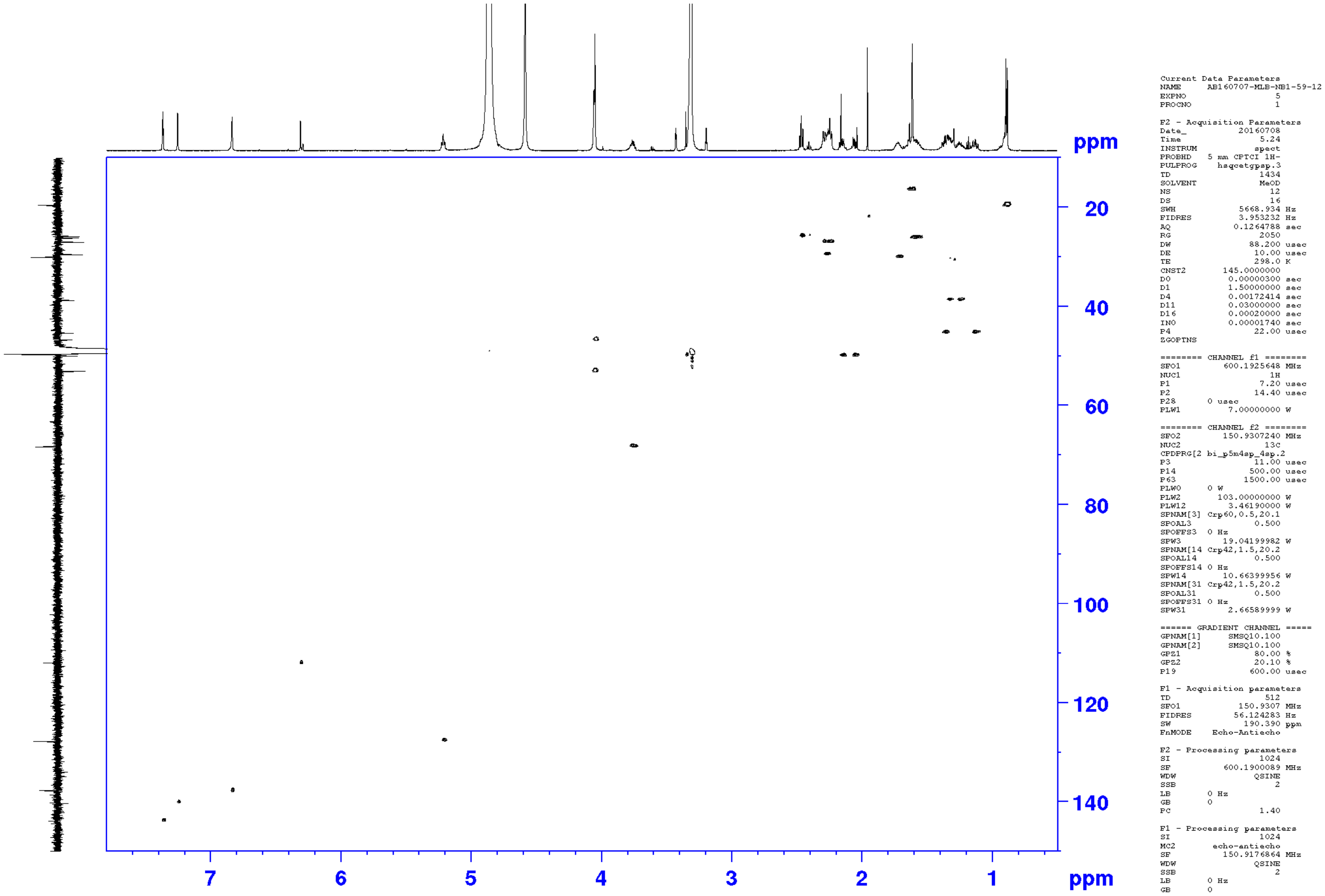

Figure S22. HMBC spectrum of pyrrolospongicin-1 (**12a**) in CD_3OD (600 MHz).

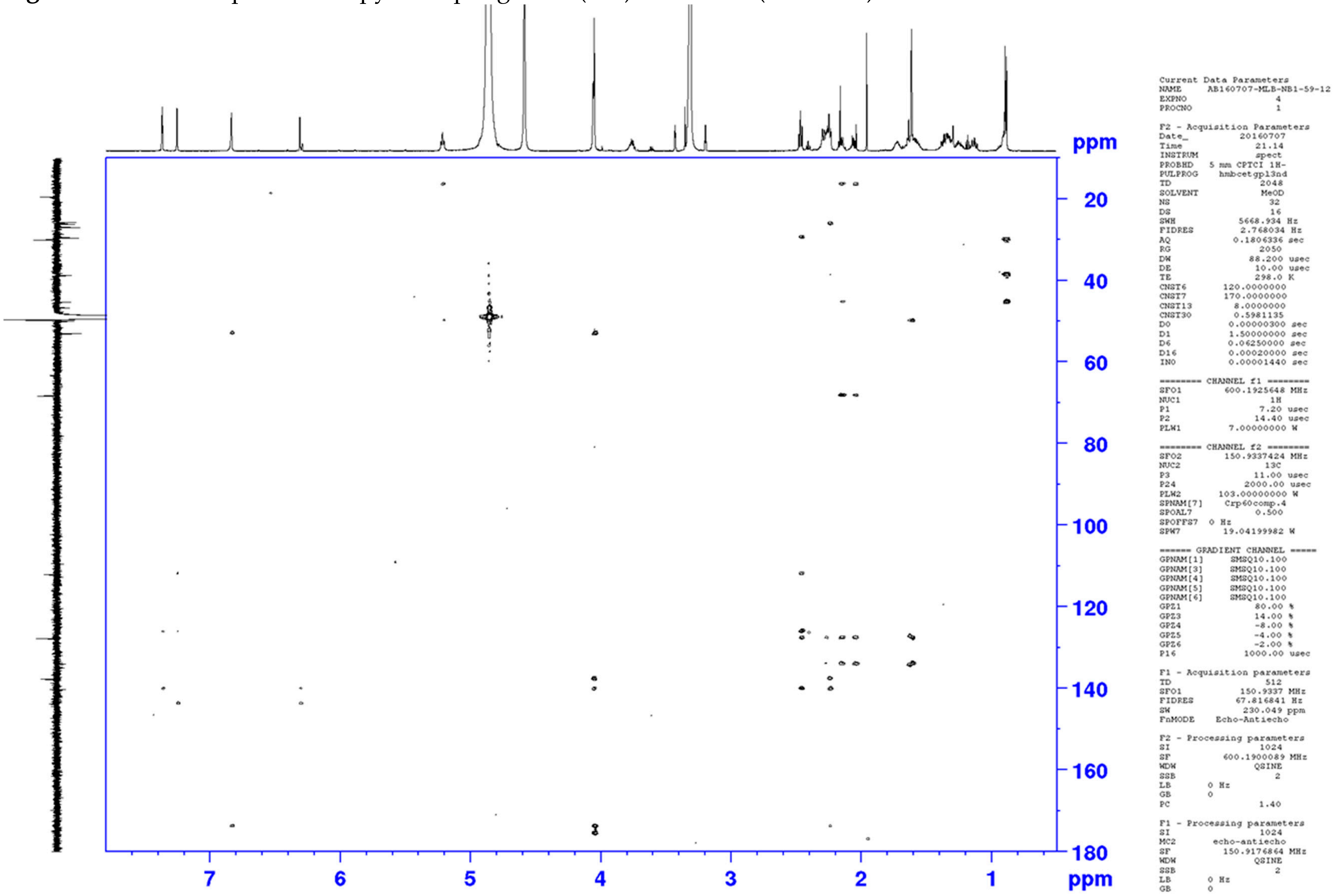

Figure S23. NOESY spectrum of pyrrolospongicin-1 (**12a**) in CD_3OD (600 MHz).

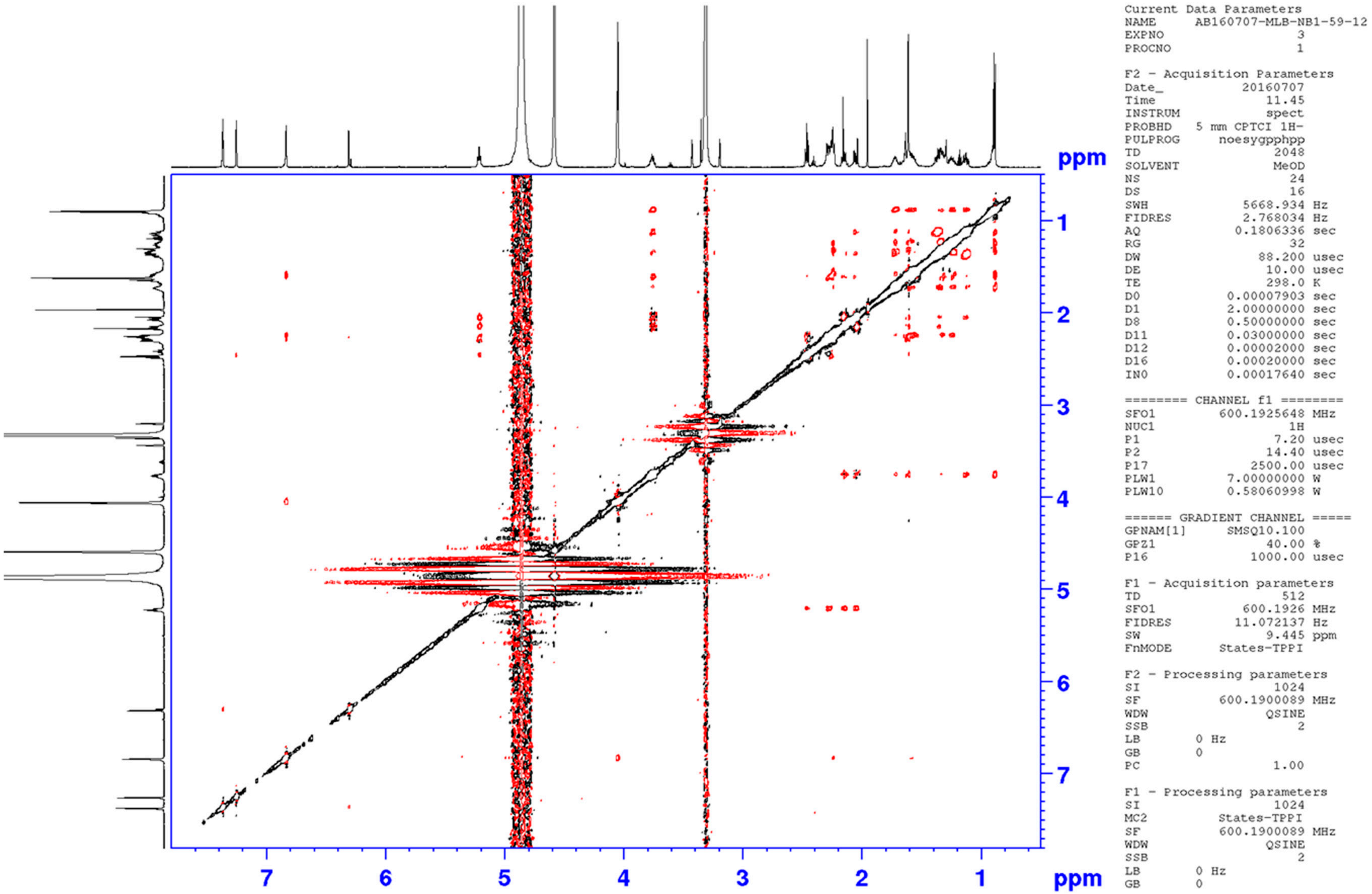

151 **Figure S24**. ^{1}H NMR spectrum of pyrrolospongicin-2 (**12b**) in CD_3OD (600 MHz).

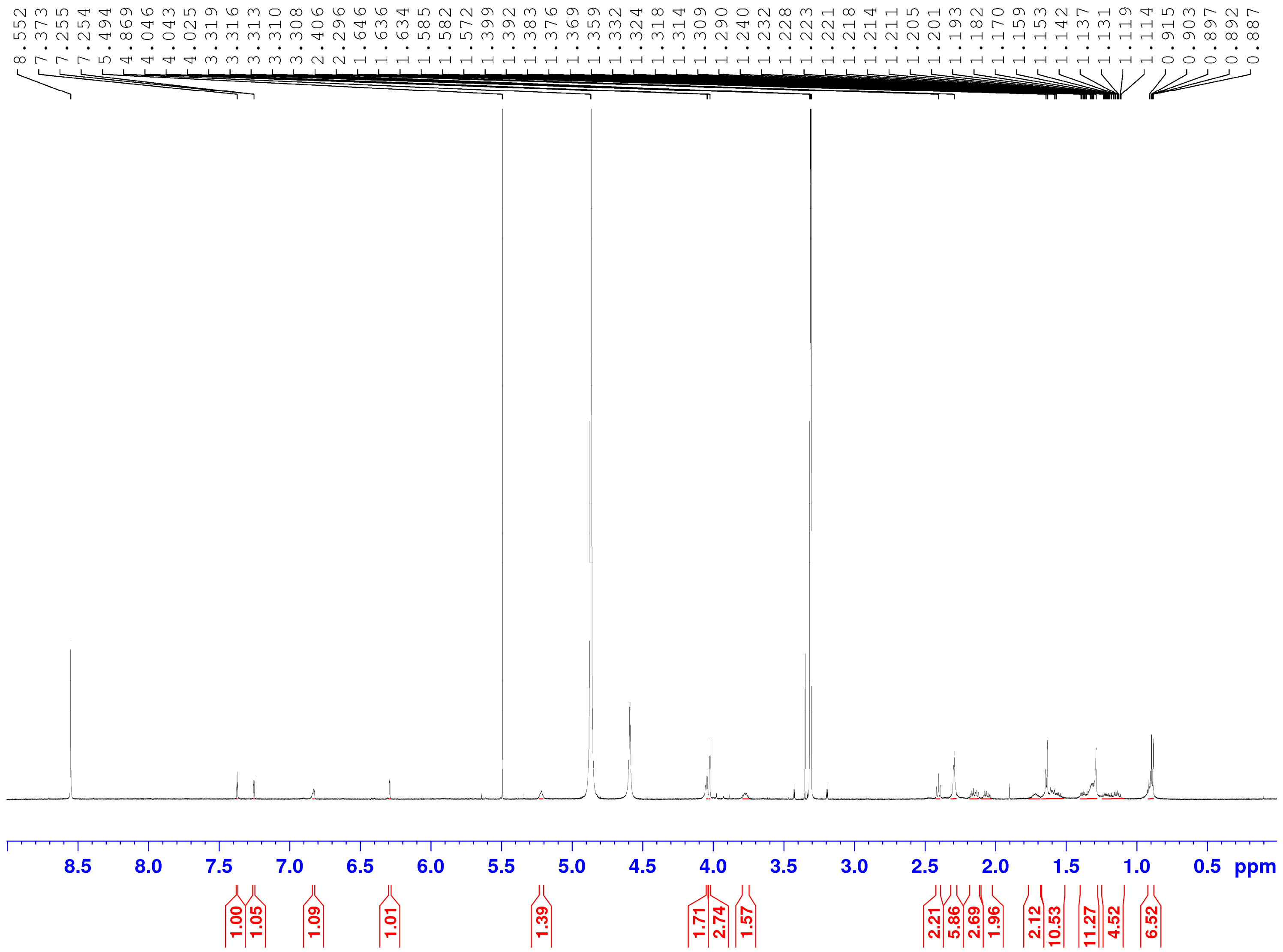

152

Figure S25. ^1H-^1H COSY spectrum of pyrrolospongicin-2 (**12b**) in CD_3OD (600 MHz).

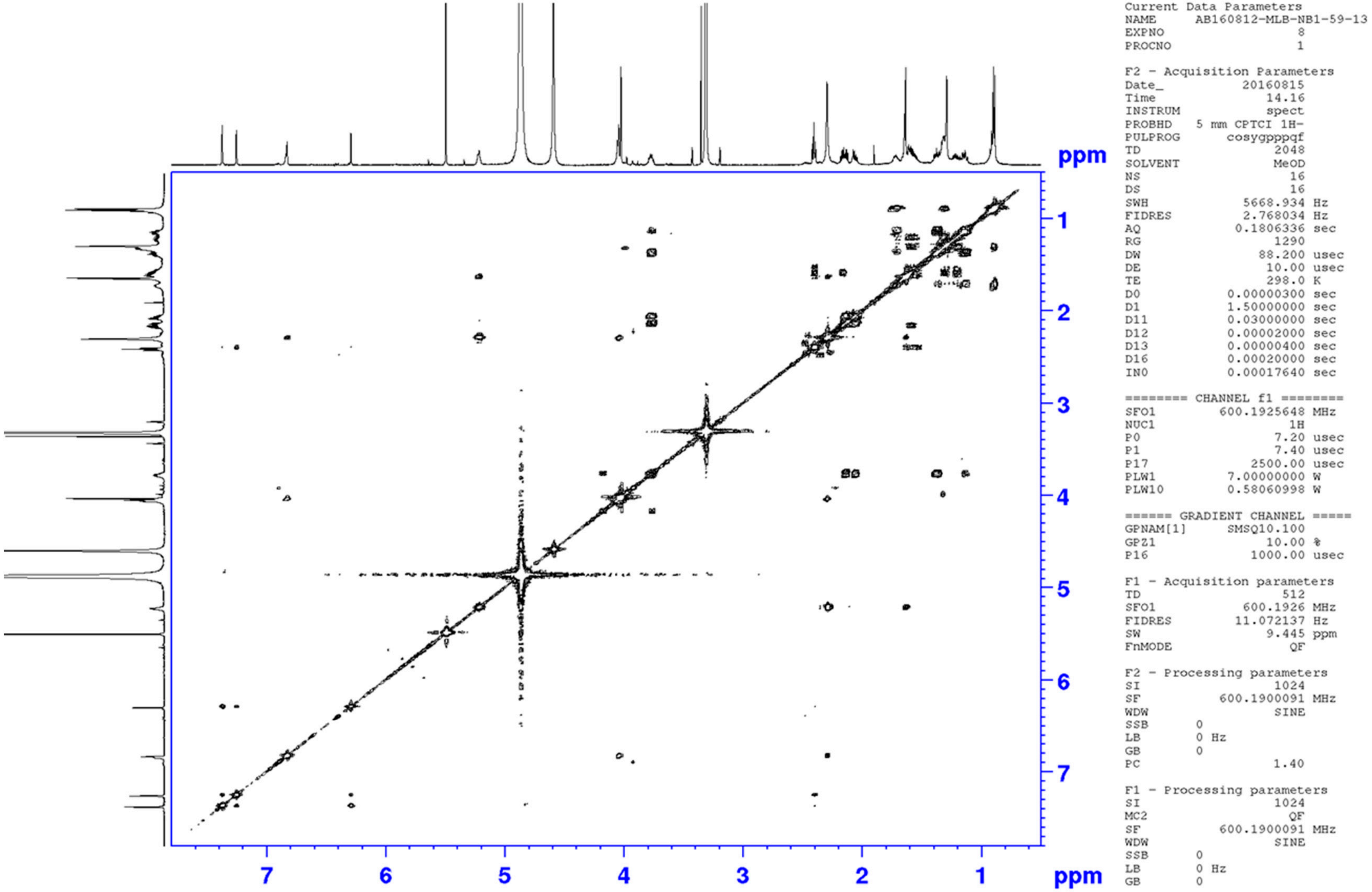

Figure S26. HSQC spectrum of pyrrolospongicin-2 (**12b**) in CD_3OD (600 MHz).

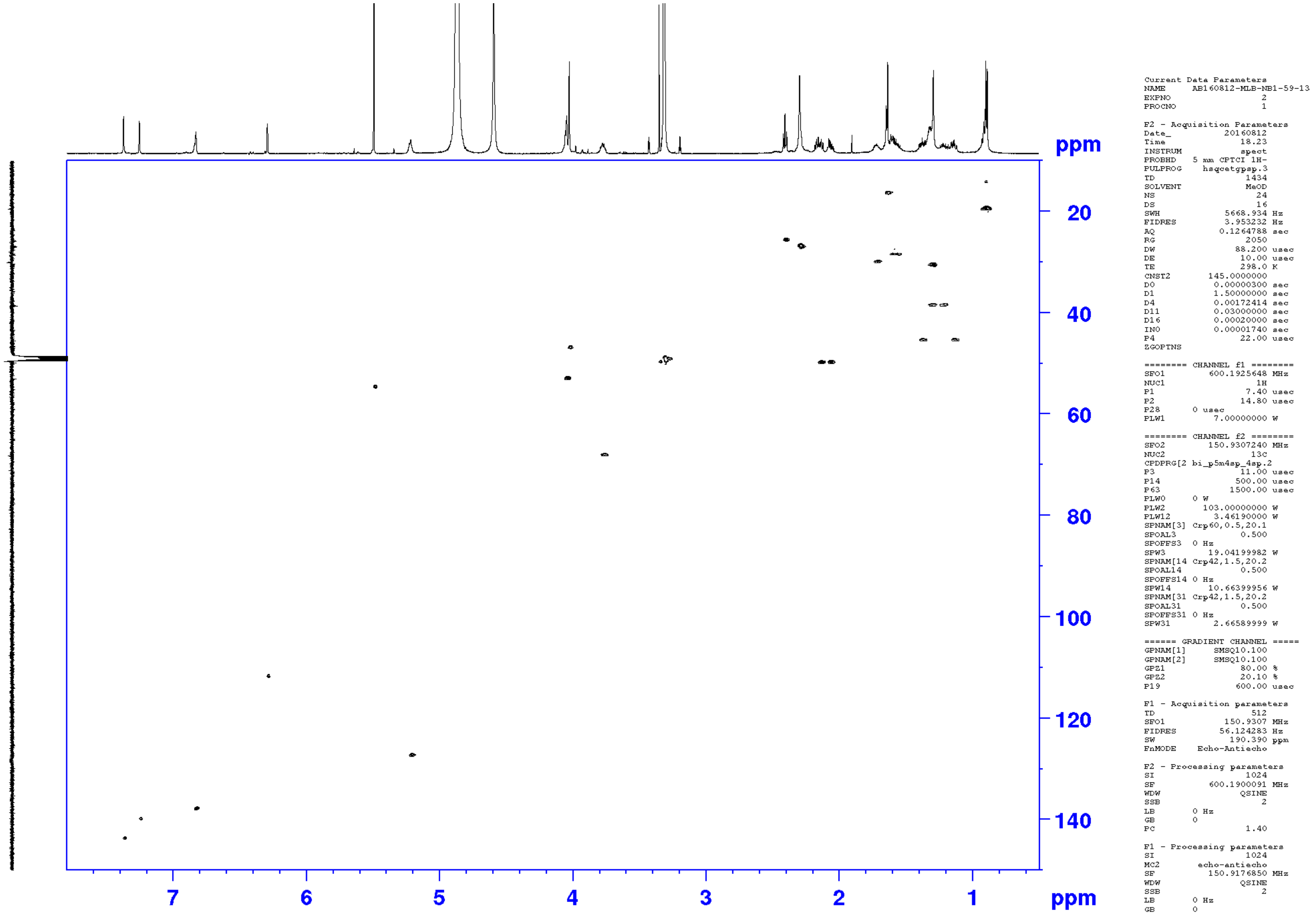

Figure S27. HMBC spectrum of pyrrolospongicin-2 (**12b**) in CD_3OD (600 MHz).

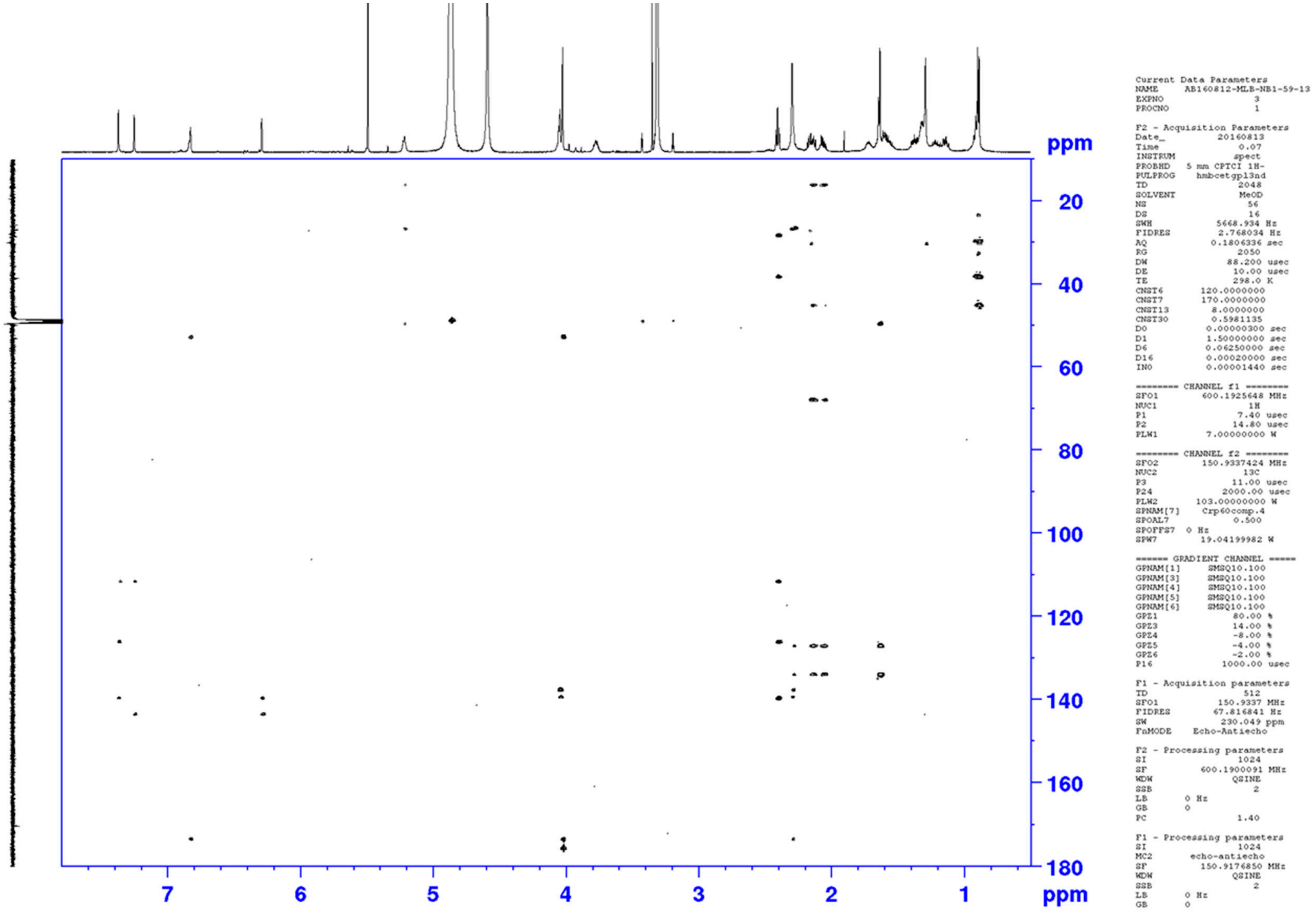

Figure S28. NOESY spectrum of pyrrolospongicin-2 (**12b**) in CD_3OD (600 MHz).

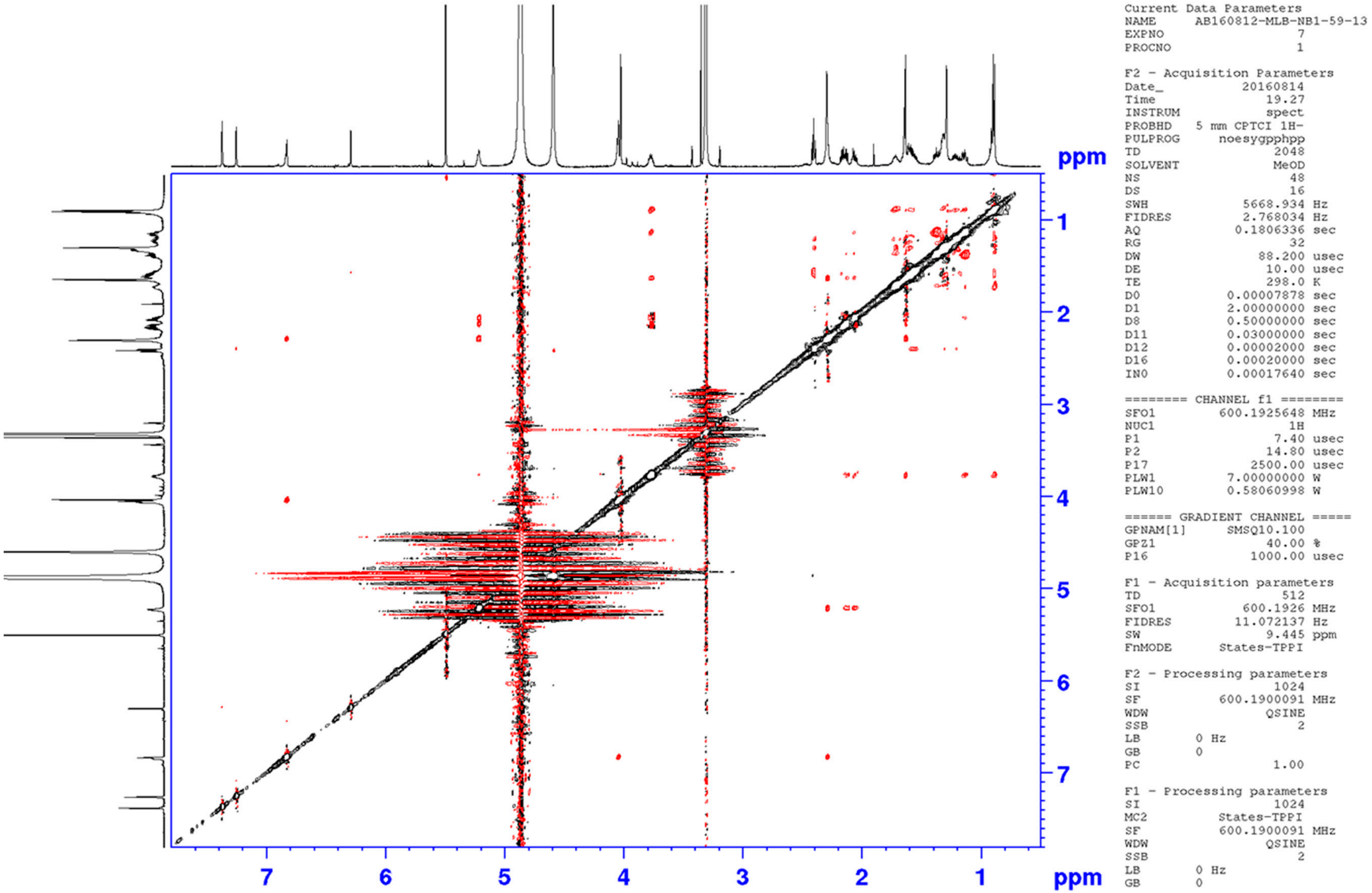

References

1. Fattorusso, E.; Minale, L.; Sodano, G.; Trivellone, E. Isolation and structure of nitenin and dihydronitenin; new furano terpenes from *Spongia nitens*. *Tetrahedron* **1971**, *27*, 3909-3917
2. Noyer, C.; Thomas, O.P.; Becerro, M.A. Patterns of chemical diversity in the Mediterranean sponge *Spongia lamella*. *PloS one* **2011**, *6*, e20844.
3. Kobayashi, M.; Chavakula, R.; Murata, O.; Sarma, N.S. Marine terpenes and terpenoids. XIV. Absolute configuration and acid-catalyzed transformation of (-)-12,13-didehydrofurospongin-1 isolated from an Arabian Sea. *Chem. Pharm. Bull.* **1992**, *40*, 599−601.
4. Manzo, E.; Ciavatta, M.L.; Villani, G.; Varcamonti, M.; Sayem, S.M.; van Soest, R.; Gavagnin, M. Bioactive terpenes from *Spongia officinalis*. *J. Nat. Prod.* **2011**, *74*, 1241−1247.
5. Shen, Y.-C.; Shih, P.-S.; Lin, Y.-S.; Lin, Y.-C.; Kuo, Y.-H.; Kuo, Y.-C.; T., K.A. Irciformonins E – K, C22-Trinorsesterterpenoids from the Sponge *Ircinia formosana*. *Helv. Chim. Acta* **2009**, *92*, 2101−2110.
6. Lumsdon, D.; Capon, R.J.; Thomas, S.G.; Beveridge, A.A. A new sesterterpene tetronic acid and a pentaprenylated p-quinol from an Australian marine sponge, *Spongia* sp. *Aust. J. Chem.* **1992**, *45*, 1321–1315.
7. Searle, P.A.; Molinski, T.F. Scalemic 12-hydroxyambliofuran and 12-acetoxyambliofuran, five tetracyclic furanoditerpenes and a furanosesterterpene from *Spongia* sp. *Tetrahedron* **1994**, *50*, 9893–9908.
8. Castillo, M.; Riu, J.; Ventura, F.; Boleda, R.; Scheding, R.; Schroder, H.F.; Nistor, C.; Emneus, J.; Eichhorn, P.; Knepper, T.P.; Jonkers, C.C.; de Voogt, P.; Gonzalez-Mazo, E.; Leon, V.M.; Barcelo, D. Inter-laboratory comparison of liquid chromatographic techniques and enzyme-linked immunosorbent assay for the determination of surfactants in wastewaters. *J. Chromatogr. A.* **2000**, *889*, 195−209.
9. Kazlauskas, R.; Murphy, P.T.; Quinn, R.J.; Wells, R.J. Two new unsymmetrically oxygenated C21 furanoterpenes from a sponge. *Tetrahedron Lett.* **1976**, *17*, 1333−1334.
10. Cimino, G.; De Stefano, S.; Minale, L.; Fattorusso, E. Minor C-21 furanoterpenes from the sponges *Spongia officinalis* and *Hippospongia communis Tetrahedron* **1972**, *28*, 267−273.
11. Cimino, G.; De Stefano, S.; Minale, L. Oxidized furanoterpenes from the sponge *Spongia officinalis*. *Experientia* **1974**, *30*, 18−20.
12. Cimino, G.; De Stefano, S.; Minale, L.; Fattorusso, E. Furospongin-1, a new C-21 furanoterpene from the sponges *Spongia officinalis* and *Hippospongia communis*. *Tetrahedron* **1971**, *27*, 4673−4679.
